# Supplementary material for: Short-term evolution and dispersal patterns of fluconazole-resistance in Candida auris clade III
Source: mBio. 2024 Dec 27;16(2):e03164-24. doi: 10.1128/mbio.03164-24 (PMC11796387; doi:10.1128/mbio.03164-24)
Supplement: Supplemental material — Figures S1-S4 and Tables S1-S4. [file mbio.03164-24-s0001.pdf]

SUPPLEMENTARY FIGURES

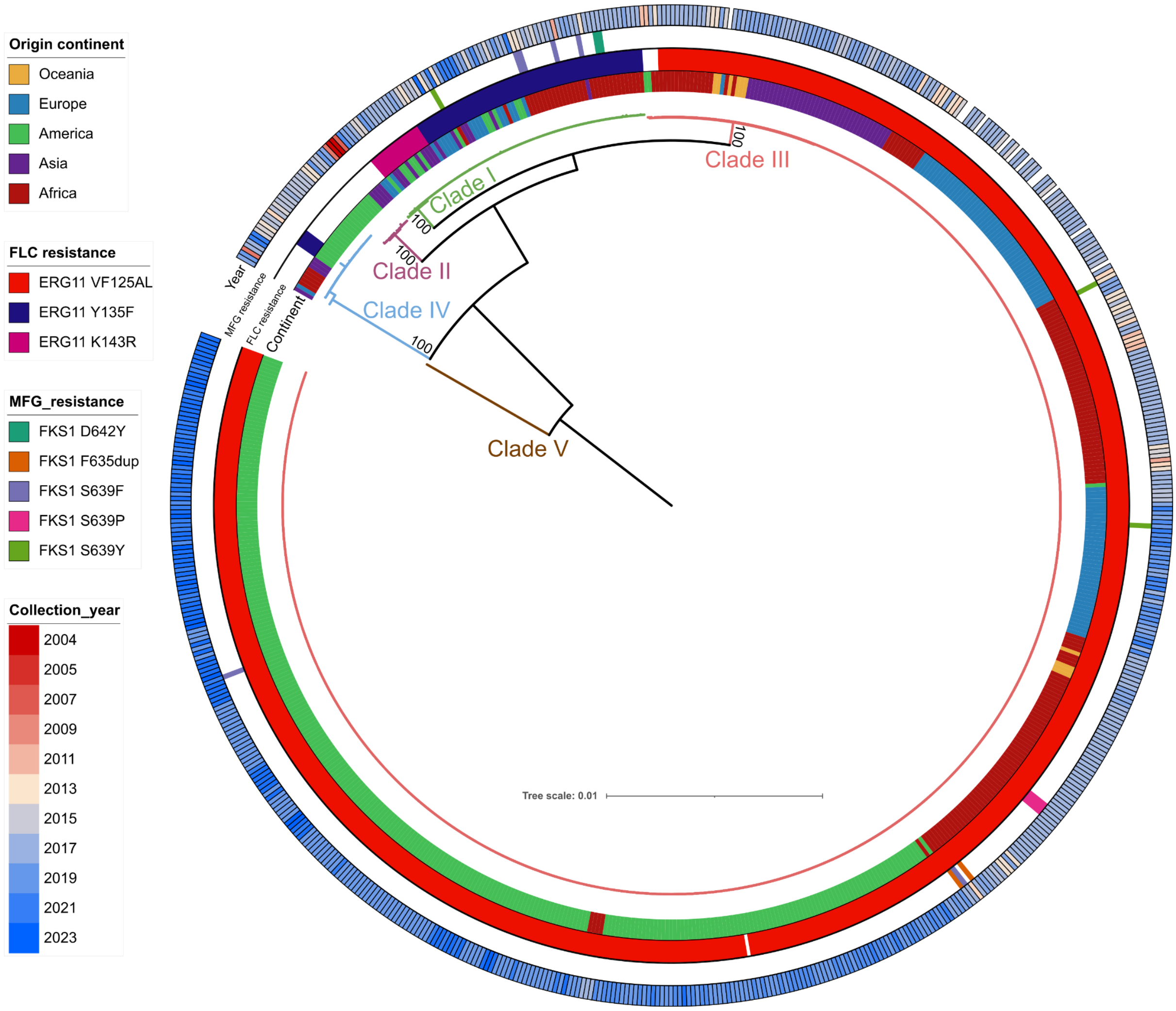

Supplementary Figure S1. Global phylogeny of all *Candida auris* isolates (n=689) obtained by maximum likelihood. The phylogeny was constructed from 194,389 SNPs. The color of each sample indicates the continent of origin. The first ring (from inner to outer) shows the sample origin, the second ring indicates mutations related to fluconazole resistance, the third ring shows mutations related to micafungin resistance, and the last ring shows the year of culture of each isolate. Blank spaces indicate that the isolates harbor the wildtype allele. Bootstrap support values of all *C.auris* clades are displayed.

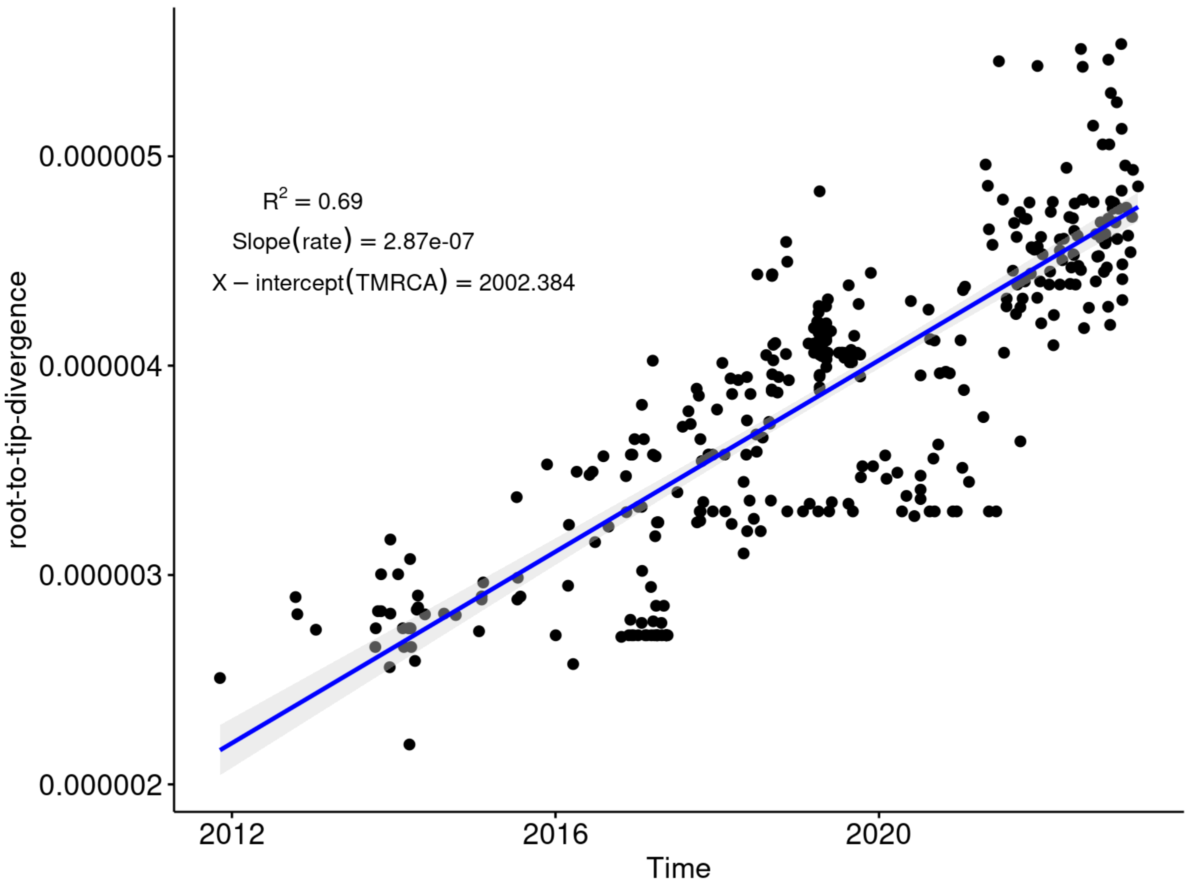

Supplementary Figure S2. Root-to-tip regression analysis of *Candida auris* clade III genomes analyzed. The slope represents the evolutionary rate obtained in Tempest. Each point represents a different genome. Shadow area around the regression line represents its 95% CI.

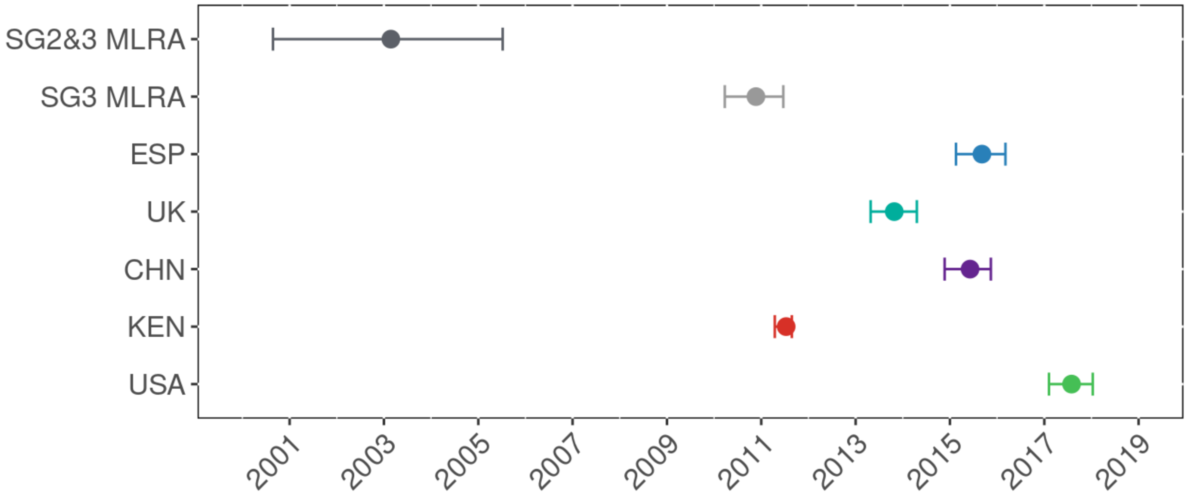

Supplementary Figure S3. Inferred dates of the MRCA of each outbreak/cluster analyzed and of those of SG2&3 and SG3, the fluconazole-resistant subgroup. Confidence intervals for each inferred point (95% CI) are shown in the graph.

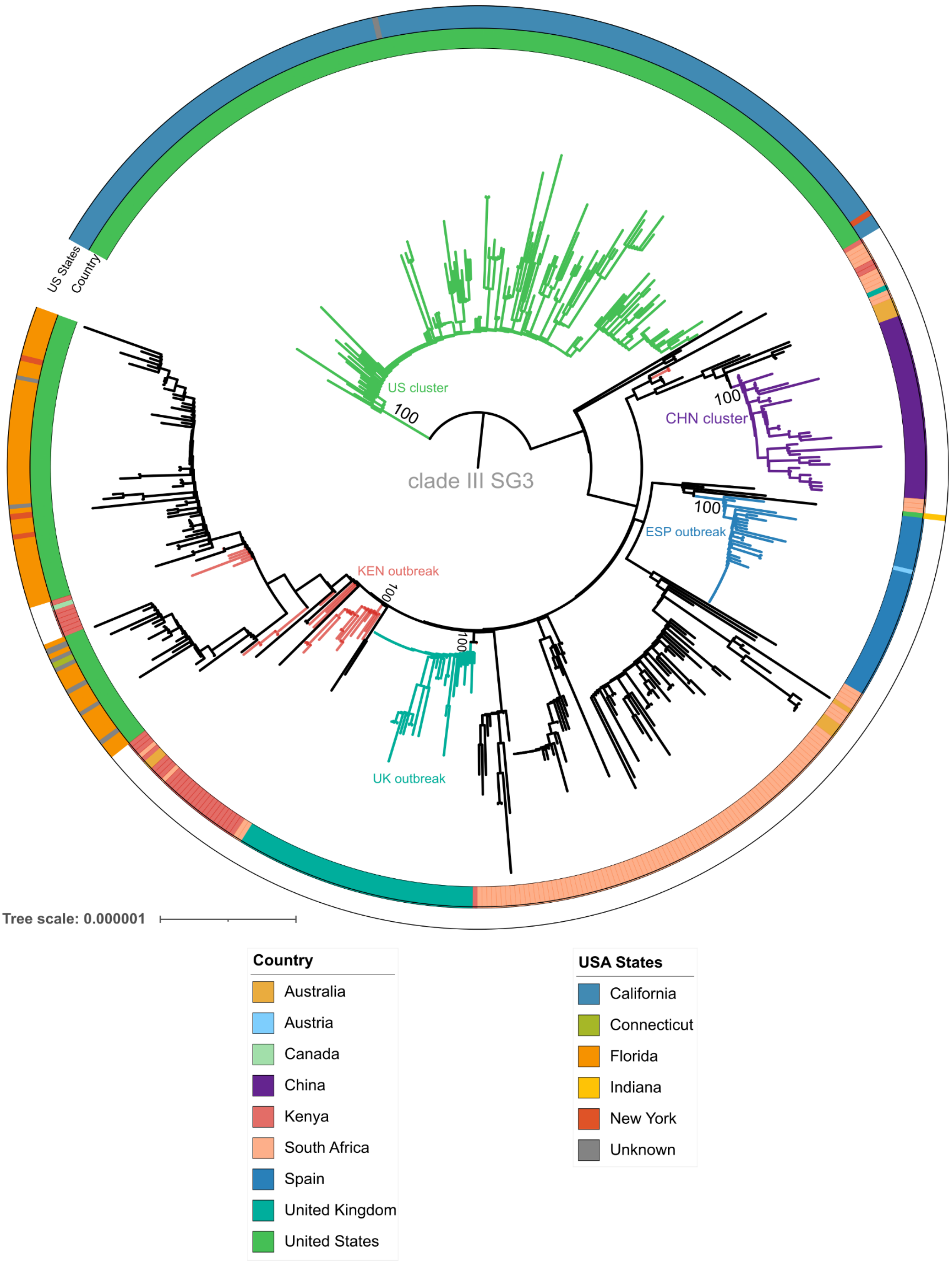

Supplementary Figure S4. Global phylogeny of all SG3 *Candida auris* isolates analyzed (n=562). The first ring (from inner to outer) indicates the origin country of each sample, the second ring shows the corresponding US state of the USA samples. Branch colors represent the different outbreaks analyzed as well as the US cluster defined in the study. Bootstrap support values of all outbreak/clusters are displayed.

Supplementary Table 1. Sequencing data of all *Candida auris* genomes analyzed in this study (n=689).

Sample ID	Phylogenetic clade	Country	Accession_number	total_reads	passed_filter_reads	reads mean length	q30 bases	q30 rate	pct duplication	gc content	median depth coverage	%horizontal coveage
3001524381	I	Canada	SRR10554762	5277788	5188856	246	1005390593	0.81	0.04	0.45	90	0.94
15B10	I	United Kingdom	ERR2299870	9311144	9201170	237	1940652466	0.90	0.69	0.45	146	0.94
16B22a	I	United Kingdom	ERR2299881	5930360	5867452	241	1226890356	0.88	0.62	0.45	92	0.94
16I34	I	United Kingdom	ERR2299892	10857192	10476226	124	1195658367	0.92	0.58	0.45	97	0.94
ACEGID-C1	I	Nigeria	SRR19214215	5758890	5697260	143	755747755	0.94	1.48	0.44	57	0.93
ACEGID-C4	I	Nigeria	SRR19214212	7520932	7362258	138	963388049	0.95	0.75	0.41	50	0.94
B11115	I	Pakistan	SRR3883429	5275382	5247698	229	1121536714	0.90	0.11	0.43	89	0.94
B11116	I	Pakistan	SRR3883430	5385520	5357536	242	1144892827	0.91	0.11	0.44	93	0.94
B11117	I	Pakistan	SRR3883431	5484030	5452702	242	1157836096	0.90	0.09	0.44	96	0.94
B11214	I	India	SRR3883445	5365210	5338608	229	1140428365	0.91	0.10	0.44	92	0.94
B11215	I	India	SRR3883446	6966864	6938526	243	1514286933	0.92	0.22	0.43	120	0.94
B11218	I	India	SRR3883450	7586682	7561266	243	1648281864	0.92	0.18	0.41	66	0.94
B13343	I	United States	SRR7909249	4221314	4169772	245	858441266	0.85	0.15	0.41	59	0.94
B13464	I	Canada	SRR10461158	2632826	2603336	243	516255115	0.84	0.02	0.46	48	0.90
B13520	I	United States	SRR7909394	4120054	4052424	244	794264929	0.83	0.04	0.43	54	0.94
B13696	I	United States	SRR7909346	4289050	4256252	247	931431713	0.89	0.06	0.44	76	0.94
B13916	I	United Arab Emirates	SRR10461157	3987618	3957074	246	886956801	0.91	0.06	0.45	74	0.93
B13917	I	United Arab Emirates	SRR10461156	4807086	4771620	247	1084575166	0.92	0.06	0.45	90	0.93
B14146	I	Saudi Arabia	SRR10461152	5009492	4924974	245	1039981756	0.87	0.08	0.46	64	0.94
B14147	I	Saudi Arabia	SRR10461151	5536746	5408866	245	1167556122	0.89	0.07	0.44	91	0.94
B14165	I	Saudi Arabia	SRR10461149	3645584	3587804	246	789964215	0.90	0.03	0.45	66	0.94
B14189	I	United Arab Emirates	SRR10461148	8323756	8301980	245	1775782787	0.88	0.18	0.44	147	0.94
B16401	I	Kenya	SRR10852068	7073500	7062028	245	1412317623	0.84	0.10	0.44	119	0.94
B16421	I	Kenya	SRR10461140	6458128	6439332	246	1156715654	0.77	0.04	0.44	101	0.94
B16422	I	Kenya	SRR10461139	6392728	6383006	248	1263637040	0.82	0.07	0.44	110	0.94
B16438	I	Kenya	SRR10461241	6221790	6215390	246	1365518970	0.90	0.15	0.44	111	0.94
B16444	I	Kenya	SRR10461237	4834638	4829296	246	1060140777	0.90	0.15	0.44	86	0.94
B16448	I	Kenya	SRR10461235	6544934	6535826	244	1380423592	0.88	0.09	0.44	116	0.94
B16472	I	Kenya	SRR10461226	6348116	6338756	243	1319288378	0.87	0.07	0.45	112	0.94
B16477	I	Kenya	SRR10461224	6679794	6672646	246	1436400058	0.88	0.17	0.43	115	0.94
B16479	I	Kenya	SRR10461223	6021070	6012116	245	1232092862	0.85	0.07	0.45	107	0.94
B16487	I	Kenya	SRR10461218	7161130	7148674	243	1436455509	0.85	0.05	0.45	126	0.94
B16490	I	Kenya	SRR10461217	6182658	6175526	245	1300047468	0.87	0.08	0.44	111	0.94
B16503	I	Kenya	SRR10461214	7616750	7606986	244	1566613176	0.86	0.09	0.45	133	0.94
B16510	I	Kenya	SRR10461210	7325356	7313868	241	1492464269	0.86	0.16	0.45	126	0.94
B16511	I	Kenya	SRR10461209	3594302	3570970	248	790003540	0.90	0.11	0.44	63	0.93
B16513	I	Kenya	SRR10461208	4272426	4242074	248	926644174	0.89	0.18	0.43	73	0.94
B17051	I	United States	SRR12073470	3735294	3618360	243	735207794	0.86	0.49	0.43	57	0.94
B17055	I	United States	SRR12073468	3802126	3694636	242	704854936	0.82	0.12	0.43	56	0.93
B17604	I	United States	SRR12073467	3931270	3804562	241	764546177	0.86	0.16	0.43	59	0.94
B17650	I	United States	SRR12073464	1850288	1821610	245	411656904	0.93	0.43	0.41	27	0.93
B18978	I	United States	SRR12787995	19632224	19375738	249	4264589545	0.89	1.12	0.45	336	0.94
B18979	I	United States	SRR12787994	17440140	17240516	248	3818981967	0.90	1.20	0.45	300	0.94
B19130	I	United States	SRR12784132	4041570	4021064	247	926063334	0.93	0.09	0.45	75	0.94
Cau1	I	Austria	SRR23080879	15952370	15070248	154	2181046074	0.94	7.70	0.47	155	0.93
Cau2	I	Austria	SRR23080878	17628050	16360022	144	2216098104	0.94	10.76	0.47	157	0.94
Cau3	I	Austria	SRR23080877	13233074	12581492	160	1879040676	0.94	12.75	0.47	132	0.94
Cau5	I	Austria	SRR23080875	1316762	1296528	218	271031199	0.96	0.64	0.44	20	0.90
CAU924-6920	I	Egypt	SRR13402267	34919602	34647078	150	4334525337	0.92	9.53	0.45	330	0.94
CNRMA15-337	I	France	SRR10723348	48215406	44418206	145	5813612178	0.91	8.52	0.46	383	0.94
CNRMA17-624	I	France	SRR10723347	39336706	36820708	141	4768337309	0.92	09.04	0.45	315	0.94
CNRMA21-252	I	France	SRR20823034	13551260	13080124	148	1721122897	0.88	3.50	0.46	138	0.93
CNRMA21-86	I	France	SRR20823037	12905632	12508912	148	1636890458	0.88	4.92	0.46	134	0.94
CNRMA21-87	I	France	SRR20823036	13990376	13594520	149	1787004304	0.88	5.67	0.46	146	0.94
CNRMA21-88	I	France	SRR20823035	14757950	14343578	149	1882717319	0.88	5.43	0.46	153	0.94
GCA 002759435.2	I	Pakistan	Reference assembly								83	0.93
MRL_128	I	South Africa	SRR14802847	5657790	5341452	208	871099603	0.80	0.73	0.44	74	0.94
MRL_3732	I	South Africa	SRR14802946	3496156	3329972	274	700866010	0.80	0.23	0.44	62	0.94

MRL_3775	I	South Africa	SRR14802945	4304442	4110996	272	818829896	0.77	0.18	0.44	75	0.94
MRL_4000	I	South Africa	SRR14802844	5060676	4918278	274	1089294906	0.83	0.41	0.44	94	0.94
MRL_4288	I	South Africa	SRR14802833	3683686	3609316	270	784447163	0.82	0.29	0.44	69	0.94
MRL_4645	I	South Africa	SRR14802930	5865740	5633698	269	1225893130	0.83	0.45	0.44	106	0.94
MRL_4934	I	South Africa	SRR14802903	7057252	6647266	188	1028866413	0.84	1.21	0.44	86	0.94
MRL_5116	I	South Africa	SRR14802898	4573258	4447408	284	1029175963	0.82	0.30	0.47	84	0.92
MRL_5173	I	South Africa	SRR14802896	7011368	6870922	249	1415893196	0.84	01.01	0.47	116	0.92
MRL_5233	I	South Africa	SRR14802894	6869630	6713390	271	1521959530	0.84	0.41	0.47	124	0.93
MRL_5425	I	South Africa	SRR14802919	2742296	2599898	266	499132985	0.76	0.23	0.44	45	0.93
MRL_5588	I	South Africa	SRR14802908	2960598	2907256	274	625060395	0.80	0.21	0.44	55	0.94
MRL_5778	I	South Africa	SRR14802851	3079524	2950330	268	576373785	0.76	0.20	0.44	52	0.93
MRL_6196	I	South Africa	SRR14802848	3086582	2949034	268	567926758	0.76	0.18	0.44	52	0.93
N18-01-797	I	Canada	SRR15192317	4087348	3818856	149	458056030	0.80	0.84	0.46	41	0.90
N18-01-802	I	Canada	SRR15192318	9806010	9114096	149	1106372446	0.81	2.73	0.45	85	0.93
N18-01-914	I	Canada	SRR13362217	6528182	6476386	292	1538745016	0.84	0.29	0.47	100	0.93
N18-02-487	I	Canada	SRR15192314	6812060	6757426	141	884544800	0.93	01.04	0.45	66	0.94
N18-02-621	I	Canada	SRR10554763	4745742	4337366	149	522982151	0.80	1.58	0.44	40	0.92
N19-02-669	I	Canada	SRR15192309	14412058	13929052	149	1808672461	0.87	28.58	0.45	68	0.94
N19-03-893	I	Canada	SRR15192315	4889274	4666164	150	632471763	0.90	31.84	0.45	21	0.90
NRZ-2015-214	I	Germany	SRR10292114	13916134	13176836	150	1737342284	0.89	1.12	0.44	144	0.94
NRZ-2017-288	I	Germany	SRR10292311	16005946	15373194	150	2064342130	0.90	1.27	0.44	170	0.94
NRZ-2017-367	I	Germany	SRR10292063	10099544	9680180	150	1293966090	0.90	1.20	0.44	105	0.94
NRZ-2017-394-1	I	Germany	SRR10277313	13704914	13068016	150	1748418556	0.90	1.31	0.44	142	0.94
RCPF-1821	I	Russia	SRR9201318	2110498	2085314	236	448587577	0.91	0.04	0.45	34	0.93
B11808	II	South Korea	SRR9645764	8317658	8234376	243	1863509072	0.94	0.53	0.43	137	0.92
B11809	II	South Korea	SRR9645762	6597258	6520550	243	1461770058	0.93	0.44	0.43	110	0.92
B12043	II	United States	SRR7909185	2988822	2955654	243	659937925	0.93	0.13	0.44	49	0.92
B12081	II	United States	SRR7909356	3743018	3709216	243	827188044	0.93	0.08	0.45	68	0.92
B13463	II	Canada	SRR10461159	4515924	4463166	242	873316710	0.83	0.05	0.43	69	0.92
B14308	II	United States	SRR10461147	5139670	5072338	245	1059416971	0.86	0.04	0.45	95	0.92
GCA 003013715.2	II	Japan	Reference assembly								82	0.92
N18-02-485	II	Canada	SRR15192319	5561904	5152536	149	631165283	0.82	11.19	0.45	28	0.90
N19-02-739	II	Canada	SRR15192310	17966640	17404600	149	2260862660	0.87	36.61	0.45	66	0.92
N19-03-592	II	Canada	SRR15192311	11649032	11255380	150	1453530449	0.86	40.55	0.44	32	0.91
AA-194	III	Spain	SRR10461267	3693206	3494316	134	421607954	0.90	1.28	0.45	35	0.92
AA-200	III	Spain	SRR10461266	4483478	4277024	141	537167425	0.89	1.10	0.45	45	0.92
AA-214	III	Spain	SRR10461265	4449694	4131440	133	494528584	0.90	1.18	0.45	41	0.92
B11222	III	South Africa	SRR3883454	5755636	5715066	242	1213413058	0.90	0.14	0.44	101	0.94
B11223	III	South Africa	SRR3883455	4843360	4812764	243	1037335201	0.91	0.24	0.44	85	0.94
B11224	III	South Africa	SRR3883456	5568522	5530204	242	1177373581	0.90	0.14	0.44	97	0.94
B11225	III	South Africa	SRR3883457	5867242	5832594	243	1273220736	0.92	0.17	0.44	104	0.94
B11226	III	South Africa	SRR3883458	4823366	4784930	242	1002832115	0.90	0.10	0.44	84	0.94
B11227	III	South Africa	SRR3883459	6486732	6442356	242	1360519056	0.90	0.11	0.44	112	0.94
B11228	III	South Africa	SRR3883461	7248118	7202832	242	1526392366	0.90	0.18	0.44	128	0.94
B11229	III	South Africa	SRR3883462	6696652	6651138	242	1393380156	0.90	0.09	0.44	116	0.94
B11230	III	South Africa	SRR3883463	5125488	5092816	227	1077805926	0.90	0.15	0.43	87	0.94
B12037	III	Canada	SRR10461253	4800968	4711638	241	1033570707	0.92	0.05	0.45	85	0.94
B12631	III	United States	SRR7909359	4627274	4529934	273	994544501	0.85	0.04	0.43	80	0.94
B16404	III	Kenya	SRR10461146	7497092	7487754	245	1543799016	0.86	0.10	0.44	134	0.94
B16406	III	Kenya	SRR10461145	7997230	7980634	245	1509968272	0.80	0.06	0.45	139	0.94
B16410	III	Kenya	SRR10461144	7113498	7105650	247	1471595401	0.86	0.09	0.45	129	0.94
B16415	III	Kenya	SRR10461143	6215684	6205738	244	1219081705	0.83	0.07	0.45	108	0.94
B16417	III	Kenya	SRR10461142	6244778	6235462	245	1303781491	0.87	0.11	0.45	112	0.94
B16419	III	Kenya	SRR10461141	7220680	7207878	243	1462163909	0.85	0.10	0.44	126	0.94
B16424	III	Kenya	SRR10461138	4955408	4926928	246	812846333	0.73	0.03	0.45	77	0.94
B16425	III	Kenya	SRR10461245	4086338	4000844	247	895852566	0.91	0.08	0.43	69	0.94
B16431	III	Kenya	SRR10461244	6683544	6673734	247	1278486342	0.80	0.07	0.44	113	0.94
B16432	III	Kenya	SRR10461211	5880352	5873960	246	1272589019	0.89	0.22	0.44	104	0.94
B16433	III	Kenya	SRR10461243	5463988	5458254	246	1193454811	0.89	0.19	0.44	97	0.94
B16436	III	Kenya	SRR10461242	5787730	5780220	245	1264558532	0.90	0.13	0.44	105	0.94

B16439	III	Kenya	SRR10461240	6226970	6220984	247	1366874917	0.90	0.15	0.45	116	0.94
B16440	III	Kenya	SRR10461239	6027098	6020764	246	1315257665	0.89	0.24	0.44	108	0.94
B16441	III	Kenya	SRR10461238	5908714	5902836	247	1304042780	0.90	0.17	0.44	108	0.94
B16445	III	Kenya	SRR10461236	6816476	6809100	246	1495407044	0.90	0.17	0.45	124	0.94
B16451	III	Kenya	SRR10461234	6388520	6380240	243	1342553192	0.88	0.10	0.45	113	0.94
B16454	III	Kenya	SRR10461233	5703180	5696034	244	1210006207	0.88	0.10	0.44	102	0.94
B16457	III	Kenya	SRR10461232	8095870	8084514	245	1749724984	0.89	0.14	0.45	149	0.94
B16459	III	Kenya	SRR10461231	7852006	7841936	245	1721284287	0.90	0.15	0.45	145	0.94
B16461	III	Kenya	SRR10461230	7879946	7867294	244	1719398142	0.90	0.11	0.45	147	0.94
B16466	III	Kenya	SRR10461229	5218430	5212186	246	1095294916	0.87	0.10	0.44	95	0.94
B16467	III	Kenya	SRR10461228	6191120	6183978	246	1299727265	0.87	0.10	0.45	113	0.94
B16469	III	Kenya	SRR10461227	4845584	4839340	245	998012277	0.86	0.09	0.44	86	0.94
B16473	III	Kenya	SRR10461225	6144790	6129758	245	1311583037	0.88	0.25	0.44	100	0.94
B16481	III	Kenya	SRR10461222	7096316	7087710	246	1537105519	0.89	0.14	0.45	131	0.94
B16482	III	Kenya	SRR10461221	7622062	7614634	246	1659533152	0.89	0.17	0.44	138	0.94
B16484	III	Kenya	SRR10461220	5958926	5950398	244	1240802460	0.87	0.10	0.45	107	0.94
B16485	III	Kenya	SRR10461219	6076162	6068112	244	1280149042	0.87	0.10	0.45	107	0.94
B16491	III	Kenya	SRR10461216	6478062	6469386	243	1344608955	0.87	0.14	0.44	114	0.94
B16496	III	Kenya	SRR10461215	6434564	6423584	244	1303198580	0.85	0.08	0.45	116	0.94
B16504	III	Kenya	SRR10461213	6049410	6039856	245	1176424186	0.82	0.07	0.45	106	0.94
B16507	III	Kenya	SRR10461212	9088848	9076220	245	1877840842	0.86	0.12	0.45	165	0.94
B16514	III	Kenya	SRR10461207	3913626	3867446	247	846006169	0.89	0.05	0.45	72	0.94
B16519	III	Kenya	SRR10461205	3804398	3773966	247	830244907	0.90	0.06	0.45	70	0.94
B16820	III	United States	SRR12073443	2538284	2495040	246	521578435	0.87	0.13	0.44	38	0.94
B17018	III	United States	SRR12073482	2617632	2594698	247	598092887	0.94	0.30	0.42	42	0.94
B17050	III	United States	SRR12073471	3658352	3542788	242	709262722	0.85	0.18	0.43	56	0.94
B17054	III	United States	SRR12073469	3238432	3135966	244	636349705	0.85	0.21	0.43	51	0.94
B17651	III	United States	SRR12073463	2657658	2633370	247	603938523	0.94	0.22	0.42	43	0.94
B17653	III	United States	SRR12073462	3348794	3308664	247	759239782	0.93	0.17	0.42	54	0.94
B17654	III	United States	SRR12073461	2517024	2492094	247	570228588	0.93	0.24	0.42	40	0.94
B17655	III	United States	SRR12073460	3251014	3205954	247	732659991	0.93	0.11	0.42	54	0.94
B17656	III	United States	SRR12073459	2279842	2257970	247	517011480	0.93	0.36	0.41	35	0.94
B17721	III	United States	SRR17577117	1881926	1797644	292	436654763	0.85	0.04	0.44	37	0.93
B17741	III	United States	SRR17577116	3652996	3548750	284	848948748	0.86	0.11	0.42	67	0.94
B17742	III	United States	SRR17577105	2873618	2773248	285	662822677	0.86	0.19	0.43	53	0.94
B17743	III	United States	SRR17577094	4294416	4171002	286	1007501127	0.86	0.10	0.42	79	0.94
B17746	III	United States	SRR17577083	3827916	3664278	287	875995863	0.85	0.16	0.42	68	0.94
B17798	III	United States	SRR12073458	3589056	3579296	246	777131062	0.89	0.16	0.44	65	0.94
B17806	III	United States	SRR17577060	2633400	2611568	247	562576763	0.88	0.11	0.43	43	0.94
B17833	III	United States	SRR17577049	2670248	2615850	247	563045751	0.88	0.11	0.46	47	0.94
B17834	III	United States	SRR17577038	4018120	3958168	247	868259658	0.90	0.26	0.45	70	0.94
B17835	III	United States	SRR14252432	2394904	2346834	247	508130581	0.89	0.08	0.46	42	0.92
B17853	III	United States	SRR17577071	3177254	3168128	248	714798596	0.92	0.13	0.45	60	0.94
B17854	III	United States	SRR17577070	6289558	6269752	248	1365806943	0.89	0.25	0.45	117	0.94
B17855	III	United States	SRR17577115	5443952	5424350	246	1183226323	0.90	0.27	0.45	100	0.94
B17856	III	United States	SRR17577114	5470020	5452600	248	1188061044	0.89	0.18	0.45	102	0.94
B17857	III	United States	SRR17577113	6343746	6321998	247	1399136289	0.91	0.28	0.45	119	0.94
B17858	III	United States	SRR17577112	6462122	6420290	247	1356848896	0.87	0.28	0.46	115	0.94
B17859	III	United States	SRR17577111	36948086	36859332	247	8159266785	0.91	0.65	0.45	674	0.94
B17860	III	United States	SRR17577110	3548080	3538772	248	799636433	0.92	0.12	0.45	67	0.94
B17861	III	United States	SRR17577109	6308810	6292720	248	1426988043	0.92	0.17	0.45	118	0.94
B17862	III	United States	SRR17577108	5573012	5558352	248	1232811467	0.90	0.18	0.45	105	0.94
B17863	III	United States	SRR17577107	7290538	7271316	247	1626215710	0.91	0.25	0.45	136	0.94
B17864	III	United States	SRR17577106	7957216	7938046	247	1766504177	0.91	0.19	0.45	151	0.94
B17865	III	United States	SRR17577104	6641324	6626102	247	1501123029	0.92	0.19	0.45	125	0.94
B17866	III	United States	SRR17577103	8381602	8359286	247	1861171178	0.91	0.20	0.45	159	0.94
B17867	III	United States	SRR17577102	5881800	5864632	248	1284449055	0.89	0.39	0.45	109	0.94
B17868	III	United States	SRR17577101	3925734	3915414	248	880832781	0.91	0.19	0.45	73	0.94
B17869	III	United States	SRR17577100	3151638	3142972	248	707816475	0.92	0.17	0.45	60	0.94
B17870	III	United States	SRR17577099	11013000	10984002	248	2387460961	0.89	0.25	0.45	204	0.94

B17871	III	United States	SRR17577098	6302060	6284210	247	1383499122	0.90	0.44	0.45	117	0.94
B17872	III	United States	SRR17577097	9073540	9045328	248	2005702664	0.90	0.46	0.45	169	0.94
B17873	III	United States	SRR17577096	9748322	9722588	248	2208124747	0.93	0.73	0.45	183	0.94
B17874	III	United States	SRR17577095	7700138	7673048	247	1688772305	0.90	0.47	0.45	143	0.94
B17875	III	United States	SRR17577093	7808782	7788252	247	1751966303	0.92	0.30	0.45	146	0.94
B17876	III	United States	SRR17577092	6915986	6897870	247	1553062149	0.92	0.23	0.45	129	0.94
B17877	III	United States	SRR17577091	6687908	6669688	247	1488697974	0.91	0.24	0.44	123	0.94
B17878	III	United States	SRR17577090	6593268	6575332	247	1484095733	0.92	0.36	0.45	124	0.94
B17904	III	United States	SRR12073457	15802650	15655708	246	2776202051	0.75	0.05	0.47	198	0.94
B17905	III	United States	SRR12073456	15315082	15262350	248	2856368531	0.78	0.05	0.45	249	0.94
B17908	III	United States	SRR12073453	17128758	16972210	247	2765785848	0.70	0.05	0.45	230	0.94
B17909	III	United States	SRR12073452	17148932	16959830	247	2718995131	0.70	0.05	0.47	227	0.94
B17910	III	United States	SRR12073451	13190766	12538378	247	1711393831	0.63	0.04	0.46	130	0.94
B17911	III	United States	SRR12073450	19829636	19649864	247	3191811670	0.70	0.06	0.46	266	0.94
B17912	III	United States	SRR12073449	18330264	18091710	247	2828805535	0.68	0.05	0.47	223	0.94
B17913	III	United States	SRR12073448	16271266	16114378	247	2595631320	0.70	0.05	0.46	218	0.94
B17914	III	United States	SRR12073447	15073378	14912050	247	2373078409	0.69	0.04	0.46	199	0.94
B17915	III	United States	SRR12073446	15122700	15032258	247	2599634499	0.74	0.05	0.45	224	0.94
B17916	III	United States	SRR17577089	12862392	12814756	247	2327662726	0.76	0.04	0.43	154	0.94
B17924	III	United States	SRR17577088	3472174	3376434	247	719440001	0.87	0.04	0.46	61	0.94
B17937	III	United States	SRR17577087	3708528	3681218	246	804929467	0.89	0.10	0.43	60	0.94
B17948	III	United States	SRR17577086	7003538	6995916	246	1457599600	0.86	0.22	0.43	117	0.94
B17949	III	United States	SRR17577085	6083422	6076622	246	1276955465	0.87	0.15	0.44	105	0.94
B17950	III	United States	SRR17577084	6806874	6799752	246	1420276074	0.86	0.12	0.44	118	0.94
B17979	III	United States	SRR12073445	14089710	13486112	249	2890487666	0.86	0.41	0.44	236	0.94
B17980	III	United States	SRR12073444	14446842	13932662	249	3007923183	0.87	0.48	0.44	242	0.94
B17981	III	United States	SRR12073442	15646558	14981432	249	3221954253	0.87	0.34	0.44	262	0.94
B18157	III	United States	SRR17577082	4466008	4293040	246	961865530	0.92	0.05	0.45	81	0.94
B18158	III	United States	SRR17577069	4392136	4268736	247	958055419	0.91	0.05	0.45	80	0.94
B18159	III	United States	SRR17577068	2950030	2931252	246	641779263	0.89	0.03	0.45	54	0.94
B18160	III	United States	SRR17577067	3668816	3609716	247	816884597	0.92	0.06	0.45	69	0.94
B18225	III	United States	SRR17577066	5877352	5771178	246	1247166718	0.88	0.08	0.44	101	0.94
B18226	III	United States	SRR17577065	3728786	3698202	249	804859618	0.88	0.14	0.42	58	0.94
B18227	III	United States	SRR17577064	4240126	4114018	246	894319726	0.89	0.08	0.44	71	0.94
B18249	III	United States	SRR17577063	3793466	3744836	246	805048759	0.88	0.07	0.44	62	0.94
B18263	III	United States	SRR17577062	2784374	2761838	247	597788379	0.88	0.08	0.43	45	0.94
B18278	III	United States	SRR17577061	1953780	1938504	247	423148996	0.89	0.09	0.42	31	0.93
B18289	III	United States	SRR17577059	6196208	6177292	246	1309985566	0.87	0.30	0.43	105	0.94
B18458	III	United States	SRR17577058	6580492	6573722	247	1418788583	0.88	0.16	0.43	116	0.94
B18460	III	United States	SRR17577057	5150408	5144728	247	1095854478	0.88	0.25	0.43	88	0.94
B18461	III	United States	SRR17577056	6377226	6370132	247	1373552003	0.88	0.15	0.44	113	0.94
B18526	III	United States	SRR12073441	5013690	5001320	240	1016138211	0.86	0.20	0.44	84	0.94
B18527	III	United States	SRR12073440	4927864	4921712	247	1036501842	0.87	0.13	0.44	86	0.94
B18528	III	United States	SRR12073439	4248120	4237126	243	826509878	0.83	0.16	0.44	70	0.94
B18529	III	United States	SRR12073438	4801180	4794620	246	992045866	0.86	0.19	0.44	83	0.94
B18530	III	United States	SRR12073437	4365506	4358952	245	886071651	0.85	0.14	0.44	75	0.94
B18531	III	United States	SRR12073436	5041322	5033362	243	1038512754	0.87	0.29	0.44	87	0.94
B18532	III	United States	SRR12073435	4942782	4933738	244	1021235440	0.86	0.15	0.44	85	0.94
B18533	III	United States	SRR12073484	5441454	5435342	246	1112242009	0.85	0.16	0.44	95	0.94
B18534	III	United States	SRR12073483	6196900	6191172	247	1291249073	0.86	0.12	0.44	105	0.94
B18540	III	United States	SRR17577055	1975940	1973086	247	393843725	0.83	0.09	0.43	33	0.93
B18657	III	United States	SRR17577054	2539958	2515640	248	536918099	0.87	0.05	0.43	43	0.94
B18665	III	United States	SRR12073481	3541600	3515052	247	784476908	0.91	0.11	0.43	60	0.94
B18666	III	United States	SRR12073480	3177494	3154882	247	702795478	0.91	0.12	0.43	55	0.94
B18667	III	United States	SRR12073479	4388646	4350332	246	965826513	0.90	0.05	0.44	76	0.94
B18669	III	United States	SRR12073477	2098948	2088512	246	466462567	0.91	0.10	0.45	39	0.93
B18683	III	Kenya	SRR14252431	5109150	5100824	247	1078985389	0.87	0.18	0.44	93	0.94
B18732	III	United States	SRR12073476	5777624	5769548	247	1222249812	0.87	0.16	0.44	103	0.94
B18733	III	United States	SRR12073475	5431074	5423432	246	1145591571	0.87	0.18	0.44	97	0.94
B18734	III	United States	SRR12073474	3763604	3733896	247	806839297	0.88	0.06	0.44	65	0.94

B18754	III	United States	SRR12073473	3380542	3374452	246	727372713	0.88	0.08	0.44	62	0.94
B18759	III	United States	SRR12073472	4572854	4566226	246	972240035	0.87	0.34	0.44	81	0.94
B18811	III	United States	SRR12526242	2626334	2605852	247	564321558	0.88	0.04	0.44	46	0.94
B18812	III	United States	SRR12784121	17730260	17496918	248	3854233327	0.89	01.03	0.45	310	0.94
B18830	III	United States	SRR12526239	2761986	2742964	245	608245221	0.91	0.07	0.44	49	0.94
B18832	III	United States	SRR12526251	4306642	4283502	247	989787918	0.94	0.09	0.44	76	0.94
B18833	III	United States	SRR12526241	3084544	3064624	247	681061351	0.90	0.09	0.44	55	0.94
B18891	III	United States	SRR12526237	1551354	1539808	247	343146467	0.91	0.12	0.44	27	0.93
B18906	III	United States	SRR12526236	1966106	1949490	247	438160748	0.91	0.09	0.44	34	0.93
B18908	III	United States	SRR12526238	1585926	1572374	247	353853128	0.92	0.08	0.44	28	0.93
B18919	III	United States	SRR12526240	2135102	2121530	246	470913527	0.90	0.04	0.44	38	0.94
B18925	III	United States	SRR12526234	1501494	1488546	246	333590161	0.92	0.07	0.44	26	0.93
B18926	III	United States	SRR12526252	1326160	1314880	247	295270647	0.91	0.09	0.44	23	0.92
B18933	III	United States	SRR12784123	13703110	13573880	248	3027550553	0.91	1.00	0.45	246	0.94
B18976	III	United States	SRR12526249	2930982	2918180	249	685431640	0.94	0.04	0.46	56	0.94
B18977	III	United States	SRR12526248	4302964	4279846	249	990171774	0.93	0.04	0.45	83	0.94
B18981	III	United States	SRR12526246	3101812	3081072	249	701452234	0.92	0.03	0.45	59	0.94
B18982	III	United States	SRR12526245	4613228	4583406	249	1032558251	0.91	0.04	0.45	88	0.94
B18995	III	United States	SRR12526244	4262834	4239918	249	974513873	0.92	0.04	0.45	81	0.94
B18996	III	United States	SRR12526243	4351686	4330842	249	1006321167	0.93	0.05	0.45	83	0.94
B18997	III	United States	SRR12784128	3564864	3518196	249	626159219	0.76	0.01	0.46	59	0.93
B19006	III	United States	SRR12784127	3993850	3930944	249	678409574	0.75	0.01	0.45	62	0.93
B19007	III	United States	SRR12784126	3247638	3182578	248	497602768	0.70	0.01	0.45	44	0.92
B19024	III	United States	SRR12784120	17112088	16902090	249	3735405900	0.90	01.03	0.45	303	0.94
B19025	III	United States	SRR12784119	3635664	3615106	249	814052855	0.91	0.03	0.45	70	0.94
B19026	III	United States	SRR12784140	3759596	3737946	250	844735766	0.91	0.03	0.45	72	0.94
B19029	III	United States	SRR12784139	2635638	2618550	249	576751947	0.89	0.02	0.45	50	0.94
B19030	III	United States	SRR12784138	3127842	3110920	249	705223257	0.91	0.03	0.46	60	0.94
B19055	III	United States	SRR12784137	21734804	21463158	249	4735344733	0.90	1.10	0.45	378	0.94
B19056	III	United States	SRR12784136	21624178	21388406	249	4743242721	0.90	1.30	0.45	381	0.94
B19062	III	United States	SRR12784135	18152762	17919214	248	3949767856	0.90	1.42	0.45	314	0.94
B19065	III	United States	SRR12784134	16583054	16361530	249	3610079141	0.89	0.92	0.45	291	0.94
B19066	III	United States	SRR12784133	15534592	15321680	249	3381757945	0.90	1.00	0.45	272	0.94
B19069	III	United States	SRR12784122	14843890	14639698	249	3224778358	0.89	1.10	0.46	260	0.94
B19131	III	United States	SRR12784131	4177042	4158364	246	962707116	0.94	0.10	0.45	77	0.94
B19132	III	United States	SRR12784129	3276342	3258050	247	741139038	0.92	0.09	0.45	60	0.94
B19285	III	United States	SRR14590379	17649242	17461022	243	3752415238	0.91	2.72	0.45	307	0.94
B19448	III	United States	SRR14590390	19630332	19392926	244	4193730727	0.90	2.17	0.45	343	0.94
B19584	III	United States	SRR14590383	3170992	3157960	239	706642671	0.94	0.03	0.45	57	0.94
B19913	III	United States	SRR14590389	34192798	33737994	246	6941507007	0.88	4.84	0.45	531	0.94
B19920	III	United States	SRR14590388	30653252	30222924	247	6200707303	0.88	5.37	0.45	472	0.94
B19944	III	United States	SRR14590384	3140754	3124924	242	671356991	0.90	0.05	0.45	57	0.94
B19983	III	United States	SRR14590386	3185168	3164580	243	712737437	0.93	0.05	0.45	58	0.94
B19985	III	United States	SRR14590385	2632844	2615990	243	585695611	0.93	0.06	0.45	48	0.94
C12-A109	III	China	SRR9316725	8663216	8662504	149	1203338027	0.93	0.05	0.44	89	0.94
C20955	III	United States	SRR17577053	14091802	13931956	247	2243806317	0.70	0.04	0.46	188	0.94
C21072	III	United States	SRR17577052	12862944	12818772	248	2342714624	0.77	0.04	0.46	206	0.94
C21086	III	United States	SRR17577051	13213342	13110200	246	2278429097	0.74	0.04	0.47	170	0.94
C21485	III	United States	SRR17577050	7522488	7494088	246	1672762236	0.92	0.30	0.43	126	0.94
C45587	III	United States	SRR17577048	3266052	3254852	246	738954301	0.93	0.18	0.41	38	0.94
C45616	III	United States	SRR17577047	6555726	6532116	246	1473301687	0.92	0.37	0.42	105	0.94
C45954	III	United States	SRR17577046	11470146	11423836	247	2078732264	0.76	0.04	0.45	183	0.94
C45960	III	United States	SRR17577045	14002802	13943122	247	2520897329	0.76	0.04	0.46	220	0.94
C45964	III	United States	SRR17577044	12490454	12002086	247	1647799907	0.63	0.04	0.46	127	0.94
C45965	III	United States	SRR17577043	15437868	15192992	248	2297953490	0.66	0.04	0.44	178	0.94
C45969	III	United States	SRR17577042	12642232	12441504	247	1886934586	0.67	0.04	0.45	155	0.94
C45991	III	United States	SRR17577041	13060286	12852010	247	1946347249	0.67	0.04	0.46	157	0.94
C46002	III	United States	SRR17577040	12322028	12066852	247	1775717938	0.65	0.04	0.47	140	0.94
C46008	III	United States	SRR17577039	15186442	14970312	248	2287235446	0.67	0.05	0.45	187	0.94
C46010	III	United States	SRR17577081	15080428	14808038	247	2215377824	0.66	0.04	0.46	174	0.94

C46013	III	United States	SRR17577080	18388214	18334234	248	3550585499	0.80	0.07	0.45	311	0.94
C46014	III	United States	SRR17577079	16756940	16718568	248	3323218831	0.82	0.07	0.45	291	0.94
C46015	III	United States	SRR17577078	16116460	16061850	247	3129036977	0.81	0.06	0.47	267	0.94
C46020	III	United States	SRR17577077	15795980	15702458	248	2811551128	0.76	0.05	0.46	242	0.94
C46022	III	United States	SRR17577076	13323826	13293886	247	2673817410	0.83	0.06	0.45	233	0.94
C46027	III	United States	SRR17577075	17403572	17358538	247	3408263357	0.81	0.06	0.46	294	0.94
C46062	III	United States	SRR17577074	16929732	16891302	247	3379955157	0.83	0.06	0.46	295	0.94
C46107	III	United States	SRR17577073	14565950	14530548	247	2888887450	0.82	0.06	0.46	252	0.94
C46121	III	United States	SRR17577072	9256272	8719722	230	1176967094	0.63	0.04	0.46	62	0.93
CA_S97	III	Australia	SRR7657927	17915438	15106504	144	1559378386	0.72	0.29	0.46	124	0.91
CA-OCPHL-CAU-00001	III	United States	SRR24806737	3054522	3022486	239	638557957	0.89	0.38	0.44	51	0.94
CA-OCPHL-CAU-00002	III	United States	SRR24806704	2685868	2654968	238	538746295	0.86	0.36	0.44	41	0.93
CA-OCPHL-CAU-00003	III	United States	SRR24806760	3700580	3657124	239	733676174	0.85	0.30	0.44	60	0.93
CA-OCPHL-CAU-00004	III	United States	SRR24806759	2742622	2711578	237	579069811	0.91	0.40	0.44	45	0.94
CA-OCPHL-CAU-00005	III	United States	SRR24806693	2475118	2452266	241	519223803	0.88	0.29	0.44	41	0.94
CA-OCPHL-CAU-00007	III	United States	SRR24806763	4680444	4635172	238	1005993624	0.92	0.79	0.44	75	0.94
CA-OCPHL-CAU-00008	III	United States	SRR24806764	2371078	2342846	237	502229657	0.91	0.37	0.44	38	0.94
CA-OCPHL-CAU-00009	III	United States	SRR24806761	4340936	4286380	236	894947662	0.89	0.62	0.44	66	0.94
CA-OCPHL-CAU-00010	III	United States	SRR24806767	4001418	3963046	241	844021404	0.89	0.42	0.44	64	0.94
CA-OCPHL-CAU-00011	III	United States	SRR24806715	2889666	2859922	240	629675351	0.92	0.46	0.44	46	0.94
CA-OCPHL-CAU-00012	III	United States	SRR24806766	2677484	2650994	240	570992587	0.90	0.36	0.44	43	0.94
CA-OCPHL-CAU-00013	III	United States	SRR24806748	2756738	2731976	240	605845973	0.93	0.47	0.44	43	0.94
CA-OCPHL-CAU-00014	III	United States	SRR24806783	2548316	2527002	240	548216833	0.91	0.81	0.43	39	0.94
CA-OCPHL-CAU-00015	III	United States	SRR24806682	6282774	6226996	239	1278708981	0.88	1.40	0.43	95	0.94
CA-OCPHL-CAU-00017	III	United States	SRR24806765	3280992	3238436	232	670927922	0.91	1.82	0.41	45	0.94
CA-OCPHL-CAU-00018	III	United States	SRR24806784	2153386	2133578	236	466911289	0.94	1.40	0.42	31	0.94
CA-OCPHL-CAU-00019	III	United States	SRR24806726	2950024	2923512	235	621649648	0.93	2.70	0.40	40	0.94
CA-OCPHL-CAU-00020	III	United States	SRR24806762	3094192	3065060	239	659049550	0.91	0.88	0.43	48	0.94
CA-OCPHL-CAU-00021	III	United States	SRR24806755	2467044	2431744	233	511989375	0.91	0.46	0.44	39	0.94
CA-OCPHL-CAU-00022	III	United States	SRR24806757	3160854	3134644	238	683730223	0.92	0.47	0.44	52	0.94
CA-OCPHL-CAU-00023	III	United States	SRR24806758	2538476	2517274	240	543382740	0.91	0.36	0.44	41	0.94
CA-OCPHL-CAU-00024	III	United States	SRR24806754	3246222	3221748	237	710629760	0.94	0.58	0.44	54	0.94
CA-OCPHL-CAU-00025	III	United States	SRR24806752	2440116	2422186	239	533645749	0.93	0.46	0.44	40	0.94
CA-OCPHL-CAU-00027	III	United States	SRR24806753	10303486	10232304	238	2207264735	0.92	1.38	0.44	159	0.94
CA-OCPHL-CAU-00028	III	United States	SRR24806751	7263804	7210660	235	1535692029	0.93	1.95	0.43	110	0.94
CA-OCPHL-CAU-00030	III	United States	SRR24806750	3159660	3130584	241	662000620	0.88	0.38	0.44	49	0.94
CA-OCPHL-CAU-00031	III	United States	SRR24806749	2736592	2710212	242	565008527	0.87	0.29	0.44	43	0.94
CA-OCPHL-CAU-00032	III	United States	SRR24806747	3883768	3846910	242	802906278	0.87	0.35	0.44	60	0.94
CA-OCPHL-CAU-00033	III	United States	SRR24806741	3384502	3349052	242	662168660	0.83	0.23	0.44	52	0.94
CA-OCPHL-CAU-00034	III	United States	SRR24806731	3490154	3455132	239	726298033	0.89	0.41	0.44	55	0.94
CA-OCPHL-CAU-00035	III	United States	SRR24806738	3146132	3116650	240	657192817	0.88	0.31	0.44	51	0.94
CA-OCPHL-CAU-00036	III	United States	SRR24806743	4561904	4522502	241	969203588	0.90	0.56	0.44	71	0.94
CA-OCPHL-CAU-00037	III	United States	SRR24806740	3326616	3288786	239	698867993	0.90	0.56	0.44	51	0.94
CA-OCPHL-CAU-00038	III	United States	SRR24806742	3266224	3236480	242	686176259	0.89	0.54	0.44	51	0.94
CA-OCPHL-CAU-00039	III	United States	SRR24806736	2275502	2255344	242	484126514	0.89	0.37	0.44	36	0.93
CA-OCPHL-CAU-00040	III	United States	SRR24806735	2859984	2834882	242	607907569	0.90	0.99	0.44	45	0.94
CA-OCPHL-CAU-00041	III	United States	SRR24806744	3145776	3116404	242	667937168	0.89	0.43	0.44	51	0.94
CA-OCPHL-CAU-00042	III	United States	SRR24806728	2090050	2071662	242	449751357	0.90	0.38	0.44	34	0.94
CA-OCPHL-CAU-00043	III	United States	SRR24806745	3713170	3677664	242	782961361	0.89	0.52	0.44	59	0.94
CA-OCPHL-CAU-00044	III	United States	SRR24806734	2168626	2148386	241	470828812	0.91	0.49	0.44	34	0.94
CA-OCPHL-CAU-00045	III	United States	SRR24806727	3820422	3789366	243	834968064	0.92	0.68	0.44	61	0.94
CA-OCPHL-CAU-00046	III	United States	SRR24806732	4522112	4479242	242	953366261	0.89	0.64	0.44	71	0.94
CA-OCPHL-CAU-00047	III	United States	SRR24806730	3742820	3700430	239	768885479	0.88	0.45	0.44	59	0.94
CA-OCPHL-CAU-00048	III	United States	SRR24806729	3965858	3920882	239	832096802	0.90	0.55	0.44	62	0.94
CA-OCPHL-CAU-00049	III	United States	SRR24806739	4426692	4389820	242	963444766	0.91	0.67	0.44	71	0.94
CA-OCPHL-CAU-00050	III	United States	SRR24806746	3014408	2985924	242	636798133	0.89	0.40	0.44	49	0.94
CA-OCPHL-CAU-00051	III	United States	SRR24806733	4000530	3967426	242	879096726	0.92	0.69	0.44	63	0.94
CA-OCPHL-CAU-00052	III	United States	SRR24806725	4987974	4941640	238	1060029554	0.91	0.95	0.43	78	0.94
CA-OCPHL-CAU-00053	III	United States	SRR24806724	3332004	3296072	236	686059161	0.89	0.55	0.43	52	0.93
CA-OCPHL-CAU-00054	III	United States	SRR24806723	4552630	4510342	239	935289417	0.88	0.94	0.43	67	0.94

CA-OCPHL-CAU-00055	III	United States	SRR24806722	4096442	4059516	238	894594675	0.94	01.01	0.43	64	0.94
CA-OCPHL-CAU-00056	III	United States	SRR24806721	2614590	2591368	240	559501908	0.91	0.44	0.43	42	0.94
CA-OCPHL-CAU-00057	III	United States	SRR24806720	4567064	4522394	237	972842834	0.92	1.10	0.43	71	0.94
CA-OCPHL-CAU-00059	III	United States	SRR24806718	3109560	3081792	239	662312140	0.91	0.40	0.44	50	0.94
CA-OCPHL-CAU-00060	III	United States	SRR24806717	4131600	4094860	235	874930938	0.93	1.92	0.42	61	0.94
CA-OCPHL-CAU-00061	III	United States	SRR24806716	3664922	3631826	238	758656013	0.90	2.34	0.41	51	0.94
CA-OCPHL-CAU-00062	III	United States	SRR24806714	3121354	3096160	241	655240859	0.90	1.86	0.41	44	0.94
CA-OCPHL-CAU-00063	III	United States	SRR24806713	3128714	3104826	240	691073083	0.94	1.24	0.43	49	0.94
CA-OCPHL-CAU-00064	III	United States	SRR24806712	2577184	2554464	239	548760528	0.92	1.36	0.42	38	0.93
CA-OCPHL-CAU-00066	III	United States	SRR24806710	2544004	2522298	239	542312478	0.91	1.11	0.42	39	0.93
CA-OCPHL-CAU-00067	III	United States	SRR24806708	1775504	1751718	229	363031634	0.92	1.41	0.41	24	0.93
CA-OCPHL-CAU-00068	III	United States	SRR24806707	4057502	4014536	234	847373146	0.91	1.15	0.43	65	0.94
CA-OCPHL-CAU-00069	III	United States	SRR24806706	7151044	7101210	235	1499320064	0.92	02.05	0.43	111	0.94
CA-OCPHL-CAU-00070	III	United States	SRR24806705	4129644	4098584	239	867735496	0.91	2.16	0.42	62	0.94
CA-OCPHL-CAU-00071	III	United States	SRR24806703	6023936	5980432	233	1235435266	0.92	3.82	0.42	87	0.94
CA-OCPHL-CAU-00072	III	United States	SRR24806702	5299452	5258336	236	1101085240	0.92	3.75	0.42	75	0.94
CA-OCPHL-CAU-00073	III	United States	SRR24806701	4874490	4837088	238	1021646628	0.92	3.60	0.41	70	0.94
CA-OCPHL-CAU-00074	III	United States	SRR24806700	1989880	1971006	238	417218666	0.90	0.60	0.43	31	0.93
CA-OCPHL-CAU-00075	III	United States	SRR24806699	3764430	3737158	234	775793125	0.94	5.69	0.40	48	0.94
CA-OCPHL-CAU-00076	III	United States	SRR24806698	5030618	4977736	237	1061161038	0.92	1.60	0.43	77	0.94
CA-OCPHL-CAU-00077	III	United States	SRR24806697	4248518	4204168	240	915784990	0.91	0.55	0.44	72	0.94
CA-OCPHL-CAU-00078	III	United States	SRR24806696	4478844	4432904	241	954885807	0.90	0.55	0.44	75	0.94
CA-OCPHL-CAU-00079	III	United States	SRR24806695	4273486	4227936	240	909639620	0.91	0.64	0.44	71	0.94
CA-OCPHL-CAU-00080	III	United States	SRR24806709	3957916	3910334	240	791723840	0.86	0.96	0.42	59	0.94
CA-OCPHL-CAU-00081	III	United States	SRR24806694	2700302	2664440	235	563668912	0.91	1.44	0.42	39	0.94
CA-OCPHL-CAU-00082	III	United States	SRR24806692	2643188	2612462	238	561077379	0.91	0.61	0.43	42	0.94
CA-OCPHL-CAU-00083	III	United States	SRR24806691	3822318	3780648	238	821761408	0.93	1.24	0.43	60	0.94
CA-OCPHL-CAU-00084	III	United States	SRR24806690	3856400	3800760	233	803740770	0.92	1.58	0.43	57	0.94
CA-OCPHL-CAU-00085	III	United States	SRR24806689	6329882	6279860	237	1304561312	0.93	5.71	0.41	84	0.94
CA-OCPHL-CAU-00086	III	United States	SRR24806688	3586218	3545428	239	757748107	0.91	1.16	0.43	55	0.94
CA-OCPHL-CAU-00087	III	United States	SRR24806687	3167974	3128704	238	669121810	0.91	1.19	0.43	49	0.93
CA-OCPHL-CAU-00088	III	United States	SRR24806686	2659186	2629142	237	565268500	0.92	1.43	0.42	40	0.94
CA-OCPHL-CAU-00089	III	United States	SRR24806685	1714248	1690612	233	356074453	0.92	1.36	0.41	24	0.93
CA-OCPHL-CAU-00090	III	United States	SRR24806684	2452628	2413892	234	499129499	0.90	1.23	0.42	35	0.94
CA-OCPHL-CAU-00091	III	United States	SRR24806683	4145926	4102970	239	901775329	0.93	1.45	0.43	63	0.94
CA-OCPHL-CAU-00092	III	United States	SRR24806681	4044180	3999334	238	853597346	0.93	03.02	0.41	54	0.94
CA-OCPHL-CAU-00093	III	United States	SRR24806680	4555346	4520784	240	940105012	0.94	7.23	0.39	52	0.94
CA-OCPHL-CAU-00094	III	United States	SRR24806679	2314542	2284572	234	474451816	0.90	1.41	0.41	30	0.93
CA-OCPHL-CAU-00095	III	United States	SRR24806678	4082414	4022782	237	856128412	0.90	0.56	0.44	67	0.94
CA-OCPHL-CAU-00096	III	United States	SRR24806677	5009908	4960270	240	1097442288	0.93	0.64	0.44	85	0.94
CA-OCPHL-CAU-00097	III	United States	SRR24806676	4707592	4655332	240	999110015	0.90	0.54	0.44	79	0.94
CA-OCPHL-CAU-00098	III	United States	SRR24806675	3684698	3640956	238	796259543	0.92	0.55	0.44	61	0.94
CA-OCPHL-CAU-00099	III	United States	SRR24806674	4058454	4008036	239	846575490	0.89	0.49	0.44	66	0.94
CA-OCPHL-CAU-00100	III	United States	SRR24806673	4988230	4937380	239	1089342761	0.93	0.72	0.44	84	0.94
CA-OCPHL-CAU-00101	III	United States	SRR24806672	3441664	3406340	240	740320107	0.91	0.50	0.44	57	0.94
CA-OCPHL-CAU-00103	III	United States	SRR24806781	2184204	2162624	240	473360172	0.92	0.35	0.44	37	0.93
CA-OCPHL-CAU-00104	III	United States	SRR24806780	3888932	3841650	238	813660316	0.90	0.53	0.44	59	0.94
CA-OCPHL-CAU-00105	III	United States	SRR24806779	4372792	4324452	241	886929858	0.87	0.71	0.43	65	0.94
CA-OCPHL-CAU-00106	III	United States	SRR24806778	3691742	3648192	240	747228172	0.86	0.44	0.44	57	0.94
CA-OCPHL-CAU-00108	III	United States	SRR24806773	3726728	3695610	241	811166798	0.92	1.22	0.43	57	0.94
CA-OCPHL-CAU-00109	III	United States	SRR24806776	2561156	2536764	240	555383607	0.92	0.47	0.44	41	0.94
CA-OCPHL-CAU-00111	III	United States	SRR24806775	3412944	3377072	241	711765031	0.88	0.47	0.44	54	0.94
CA-OCPHL-CAU-00113	III	United States	SRR24806770	2862722	2839198	241	620954991	0.91	0.39	0.44	47	0.94
CA-OCPHL-CAU-00114	III	United States	SRR24806768	2313404	2291764	242	486781591	0.88	0.27	0.44	37	0.94
CA-OCPHL-CAU-00115	III	United States	SRR24806769	8894024	8831360	225	1813218057	0.93	1.51	0.44	128	0.94
Cau4	III	Austria	SRR23080876	1376362	1368138	239	306844183	0.94	0.39	0.45	23	0.92
CT-WCPHL-CAU-224150	III	United States	SRR24621173	14289808	13784110	145	1730293730	0.89	2.58	0.44	140	0.94
DN474116W-A1	III	United Kingdom	ERR2300769	12236970	11916772	124	1367512962	0.93	0.63	0.45	115	0.94
DN474116W-A6	III	United Kingdom	ERR2300809	13758548	13337438	124	1518411558	0.92	0.69	0.44	124	0.94
DN474116W-B1	III	United Kingdom	ERR2300770	11754398	11379674	124	1296388125	0.92	0.57	0.45	111	0.94

DN474116W-B6	III	United Kingdom	ERR2300810	13080518	12677878	124	1445475258	0.92	0.65	0.44	120	0.94
DN474116W-C1	III	United Kingdom	ERR2300771	12147742	11781658	124	1337895469	0.92	0.57	0.44	113	0.94
DN474116W-D1	III	United Kingdom	ERR2300772	12621520	12250538	124	1396734369	0.92	0.62	0.44	116	0.94
DN474116W-D4	III	United Kingdom	ERR2300796	12228422	11909392	124	1366960164	0.93	0.70	0.44	111	0.94
DN474116W-D5	III	United Kingdom	ERR2300804	11273102	10944482	124	1251130765	0.92	0.64	0.44	102	0.94
DN474116W-E3	III	United Kingdom	ERR2300789	11247734	10892912	124	1239192904	0.92	0.57	0.45	105	0.94
DN474116W-E4	III	United Kingdom	ERR2300797	11372248	11050218	124	1259285818	0.92	0.71	0.44	101	0.94
DN474116W-F4	III	United Kingdom	ERR2300798	11840088	11406552	124	1298240011	0.92	0.68	0.44	106	0.94
DN474116W-G2	III	United Kingdom	ERR2300783	5122322	4986818	124	572439030	0.93	0.62	0.44	46	0.94
GCF 002775015.1	III	South Africa	Reference assembly								85	0.95
Hoist	III	United Kingdom	SRR7976583	4450634	4414550	146	564114733	0.87	0.42	0.46	49	0.93
MRL_2491	III	South Africa	SRR14802846	7663778	7480790	250	1603027131	0.87	0.86	0.44	95	0.94
MRL_2526	III	South Africa	SRR14802845	7392338	7014674	244	1305073078	0.79	0.30	0.46	112	0.94
MRL_2546	III	South Africa	SRR14802843	5374628	5130056	250	1019509019	0.81	0.25	0.47	95	0.93
MRL_2547	III	South Africa	SRR14802842	6030010	5787124	258	1213980943	0.82	0.30	0.47	114	0.93
MRL_2566	III	South Africa	SRR14802841	5639246	5486768	267	1235211249	0.85	0.66	0.44	66	0.94
MRL_2609	III	South Africa	SRR14802840	7035042	6816054	257	1480716643	0.86	0.29	0.47	134	0.94
MRL_2634	III	South Africa	SRR14802839	6714804	6606736	274	1509072410	0.85	0.38	0.47	126	0.93
MRL_2678	III	South Africa	SRR14802838	11174912	10862128	257	2366289033	0.86	0.40	0.47	213	0.94
MRL_2736	III	South Africa	SRR14802836	3931976	3794814	273	859443004	0.84	0.49	0.43	42	0.93
MRL_2859	III	South Africa	SRR14802835	6739870	6619172	270	1511115837	0.86	0.58	0.47	117	0.93
MRL_2980	III	South Africa	SRR14802834	6216050	6027492	257	1233685290	0.81	0.22	0.47	111	0.93
MRL_3225	III	South Africa	SRR14802832	6750036	6644258	275	1543238995	0.86	0.50	0.47	123	0.92
MRL_3269	III	South Africa	SRR14802940	5456346	5284412	274	1150750857	0.81	0.24	0.46	96	0.93
MRL_3289	III	South Africa	SRR14802938	4979244	4816118	259	1063999034	0.86	0.70	0.44	59	0.94
MRL_3338	III	South Africa	SRR14802937	4822152	4696324	262	1045937019	0.86	0.57	0.44	61	0.93
MRL_3345	III	South Africa	SRR14802936	6697790	6558144	270	1504561257	0.86	0.72	0.46	128	0.93
MRL_3402	III	South Africa	SRR14802935	6878652	6639482	277	1521504209	0.84	0.23	0.47	123	0.93
MRL_3405	III	South Africa	SRR14802934	6252060	6101750	279	1453069499	0.87	0.44	0.46	110	0.93
MRL_3406	III	South Africa	SRR14802933	5381596	5222666	270	1119154388	0.80	0.28	0.47	92	0.92
MRL_3411	III	South Africa	SRR14802932	5495048	5341144	272	1213545672	0.85	0.52	0.44	68	0.94
MRL_3511	III	South Africa	SRR14802931	4618190	4438828	256	947169202	0.84	0.56	0.44	54	0.94
MRL_3560	III	South Africa	SRR14802928	4645894	4525462	282	1026944335	0.81	0.24	0.47	87	0.92
MRL_3561	III	South Africa	SRR14802927	5724476	5553834	258	1186993510	0.83	0.29	0.47	108	0.93
MRL_3562	III	South Africa	SRR14802926	3820820	3701212	270	843335102	0.87	0.43	0.42	52	0.94
MRL_3589	III	South Africa	SRR14802925	4610394	4466008	277	971080922	0.80	0.23	0.47	87	0.92
MRL_3706	III	South Africa	SRR14802924	3986634	3835112	258	757483391	0.78	0.21	0.44	49	0.93
MRL_3758	III	South Africa	SRR14802923	4318678	4083972	274	872763927	0.80	0.28	0.43	44	0.94
MRL_3770	III	South Africa	SRR14802922	5654876	5524608	267	1185806974	0.81	0.40	0.47	105	0.93
MRL_3788	III	South Africa	SRR14802921	3039478	2922670	243	568663114	0.86	0.22	0.45	33	0.93
MRL_4152	III	South Africa	SRR14802920	5212046	5006988	258	1022698756	0.80	0.18	0.47	98	0.93
MRL_4414	III	South Africa	SRR14802918	5766556	5541262	244	1092906817	0.82	0.31	0.47	98	0.94
MRL_4603	III	South Africa	SRR14802916	6273140	5884684	254	1139844553	0.79	0.25	0.47	106	0.94
MRL_4642	III	South Africa	SRR14802915	3458822	3260014	271	644769653	0.77	0.06	0.47	58	0.91
MRL_4742	III	South Africa	SRR14802914	3022904	2889890	260	583503342	0.79	0.27	0.44	37	0.93
MRL_4836	III	South Africa	SRR14802913	5324000	5093820	269	1135852095	0.85	0.20	0.47	103	0.93
MRL_4845	III	South Africa	SRR14802912	4298606	4171232	287	991945662	0.84	0.13	0.47	76	0.92
MRL_4888	III	South Africa	SRR14802911	8291084	7940118	229	1486231072	0.83	0.55	0.48	121	0.94
MRL_4895	III	South Africa	SRR14802910	6416084	6121008	235	1172085196	0.83	0.51	0.47	104	0.94
MRL_4925	III	South Africa	SRR14802907	4781174	4642686	245	887227511	0.80	0.29	0.45	74	0.94
MRL_4926	III	South Africa	SRR14802906	4375190	4198310	286	930752033	0.80	0.11	0.46	81	0.93
MRL_4928	III	South Africa	SRR14802905	5988056	5715754	261	1173561393	0.81	0.22	0.47	108	0.93
MRL_4930	III	South Africa	SRR14802904	5622858	5434778	279	1229596720	0.82	0.24	0.47	103	0.93
MRL_4943	III	South Africa	SRR14802902	4839400	4732968	265	1005439912	0.81	0.37	0.47	86	0.92
MRL_4944	III	South Africa	SRR14802901	6797382	6515948	236	1247049112	0.82	0.43	0.48	102	0.94
MRL_5006	III	South Africa	SRR14802900	6052484	5790038	231	1052257974	0.81	0.51	0.44	90	0.94
MRL_5109	III	South Africa	SRR14802899	6513622	6392716	249	1309593176	0.83	0.74	0.47	113	0.93
MRL_5222	III	South Africa	SRR14802895	2933794	2806758	287	617727792	0.79	0.09	0.46	53	0.91
MRL_5268	III	South Africa	SRR14802893	3842800	3703292	269	815621054	0.85	0.15	0.47	74	0.92
MRL_5305	III	South Africa	SRR14802892	4482102	4275632	274	882438580	0.79	0.09	0.46	79	0.92

MRL_5386	III	South Africa	SRR14802891	3822814	3711634	275	810039963	0.81	0.15	0.46	69	0.92
MRL_5405	III	South Africa	SRR14802890	3972020	3771144	224	661252030	0.80	0.53	0.45	57	0.94
MRL_5483	III	South Africa	SRR14802889	5727614	5396952	248	1025411743	0.79	0.27	0.47	95	0.93
MRL_5543	III	South Africa	SRR14802888	2145688	2027700	264	413744627	0.79	0.12	0.47	39	0.91
MRL_5547	III	South Africa	SRR14802887	3615600	3439076	276	712242788	0.78	0.07	0.47	62	0.91
MRL_5561	III	South Africa	SRR14802885	6849134	6575150	255	1351865042	0.82	0.32	0.47	122	0.93
MRL_5574	III	South Africa	SRR14802884	4079244	3880748	245	750716791	0.80	0.28	0.47	66	0.93
MRL_5580	III	South Africa	SRR14802883	4809588	4664178	286	1108221170	0.84	0.19	0.46	89	0.93
MRL_5585	III	South Africa	SRR14802882	3402762	3267526	276	681736087	0.79	0.07	0.47	59	0.91
MRL_5590	III	South Africa	SRR14802897	3241404	3098568	269	597845370	0.75	0.18	0.44	58	0.93
MRL_5591	III	South Africa	SRR14802886	4718702	4555876	274	1004145199	0.83	0.26	0.44	91	0.93
MRL_5593	III	South Africa	SRR14802881	4111724	3932824	291	925523782	0.83	0.11	0.47	73	0.92
MRL_5624	III	South Africa	SRR14802880	5752240	5550392	275	1203259430	0.81	0.10	0.47	111	0.93
MRL_5625	III	South Africa	SRR14802879	7797034	7538822	263	1635214171	0.84	0.24	0.47	148	0.94
MRL_5704	III	South Africa	SRR14802878	5968524	5635478	263	1185619213	0.82	0.15	0.47	109	0.94
MRL_5714	III	South Africa	SRR14802875	5579542	5361342	246	1014521878	0.80	0.42	0.45	93	0.94
MRL_5715	III	South Africa	SRR14802944	1989906	1862758	264	341930452	0.74	0.22	0.44	33	0.92
MRL_5716	III	South Africa	SRR14802858	3135498	3047620	270	595887416	0.75	0.12	0.44	58	0.93
MRL_5734	III	South Africa	SRR14802877	3461110	3310638	283	777264062	0.85	0.37	0.46	59	0.93
MRL_5762	III	South Africa	SRR14802852	4086388	3946920	269	858587739	0.83	0.31	0.45	78	0.94
MRL_5771	III	South Africa	SRR14802876	3917376	3753450	252	729464053	0.79	0.26	0.45	54	0.94
MRL_5946	III	South Africa	SRR14802874	3463934	3303130	260	649089509	0.78	0.21	0.44	45	0.93
MRL_5948	III	South Africa	SRR14802850	3998554	3920254	271	845742553	0.81	0.22	0.44	77	0.94
MRL_5953	III	South Africa	SRR14802873	4644260	4455078	248	851725133	0.79	0.30	0.45	68	0.94
MRL_6057	III	South Africa	SRR14802849	4405056	4229654	262	800540594	0.76	0.17	0.44	76	0.94
MRL_6241	III	South Africa	SRR14802872	5299628	5032832	219	878809540	0.81	0.61	0.44	75	0.94
MRL_6277b	III	South Africa	SRR14802871	6187254	5933934	245	1167364441	0.82	0.34	0.47	104	0.93
MRL_B159-2	III	South Africa	SRR14802870	2665340	2536684	281	542713595	0.78	0.05	0.46	49	0.90
MRL_B195-1	III	South Africa	SRR14802869	3969200	3807556	275	827182995	0.81	0.11	0.47	72	0.92
MRL_B235-1	III	South Africa	SRR14802867	9816440	9546858	260	2085546914	0.85	0.37	0.47	185	0.94
MRL_B296-3	III	South Africa	SRR14802942	8007020	7793514	256	1671022632	0.85	0.32	0.47	151	0.93
MRL_S158-1	III	South Africa	SRR14802939	6248462	6061028	260	1282074331	0.83	0.21	0.47	117	0.93
MRL_S16	III	South Africa	SRR14802943	3641636	3467988	286	784715895	0.80	0.10	0.47	53	0.90
MRL_S19	III	South Africa	SRR14802868	5144122	4883316	261	1046276884	0.83	0.23	0.47	97	0.93
MRL_S2	III	South Africa	SRR14802866	3826602	3654774	264	753529116	0.80	0.12	0.47	71	0.93
MRL_S200-1	III	South Africa	SRR14802865	4219298	4108456	270	890728811	0.82	0.19	0.46	78	0.92
MRL_S26	III	South Africa	SRR14802864	5180164	4952912	267	1065030135	0.81	0.17	0.47	101	0.93
MRL_S27	III	South Africa	SRR14802863	3764700	3605982	287	830119126	0.81	0.16	0.46	53	0.91
MRL_S29	III	South Africa	SRR14802862	3792372	3596072	285	832564302	0.82	0.16	0.46	53	0.91
MRL_S31	III	South Africa	SRR14802861	2696342	2634982	292	638993390	0.86	0.10	0.46	45	0.90
MRL_S316-2	III	South Africa	SRR14802860	4689680	4529810	278	1019080250	0.83	0.18	0.47	80	0.92
MRL_S33	III	South Africa	SRR14802859	4184868	4128618	275	982351849	0.89	0.25	0.47	73	0.91
MRL_S353-2	III	South Africa	SRR14802857	3405424	3256224	280	692200356	0.78	0.06	0.46	63	0.91
MRL_S360-1	III	South Africa	SRR14802856	6166766	5898884	255	1203020212	0.82	0.22	0.47	112	0.93
MRL_S4	III	South Africa	SRR14802855	4926756	4752288	260	1002202885	0.83	0.18	0.47	88	0.93
MRL_S6	III	South Africa	SRR14802854	5054444	4840072	279	1093691669	0.82	0.18	0.47	75	0.92
MRL_W-KNH046	III	South Africa	SRR14802853	6864616	6654218	265	1458765124	0.84	0.24	0.47	132	0.93
N18-02-486	III	Canada	SRR15192316	11575084	10601206	149	1249406743	0.79	05.07	0.45	70	0.93
N19-01-047	III	Canada	SRR15192306	8422490	8108054	150	1054851992	0.87	14.18	0.44	63	0.94
NICU10-A58	III	China	SRR9316776	66870826	66209164	149	9135636096	0.92	10.45	0.44	585	0.94
NICU11-A77	III	China	SRR9316803	5092864	5092440	149	708735892	0.93	0.03	0.45	56	0.94
NICU12-A79	III	China	SRR9316800	7326886	7326002	149	1043856814	0.95	0.04	0.45	79	0.94
NICU13-A93	III	China	SRR9316812	54567104	54345764	149	7599900878	0.93	10.41	0.45	531	0.94
NICU14-A96	III	China	SRR9316734	48535296	48296686	149	6903995854	0.95	17.05	0.45	427	0.94
NICU15-A100	III	China	SRR9316790	42455724	42163752	149	5913117617	0.94	11.34	0.45	400	0.94
NICU16-A101	III	China	SRR9316791	41495616	41254448	149	5830078876	0.94	8.82	0.45	408	0.94
NICU2-A13	III	China	SRR9316739	7236398	7235626	149	1031901632	0.95	0.04	0.45	79	0.94
NICU3-A15	III	China	SRR9316741	50017136	49756888	149	7025302356	0.94	10.96	0.45	484	0.94
NICU4-A19	III	China	SRR9316745	65394404	65138016	149	9348367033	0.96	13.46	0.45	622	0.94
NICU5-A36	III	China	SRR9316756	47093506	46920476	149	6741422614	0.96	12.98	0.45	453	0.94

NICU6-A41	III	China	SRR9316762	7986986	7985996	149	1135862810	0.95	0.05	0.45	86	0.94
NICU7-A42	III	China	SRR9316761	49686766	49416792	149	6970145755	0.94	11.50	0.45	471	0.94
NICU8-A43	III	China	SRR9316760	63244992	62886662	149	8755410685	0.93	9.55	0.45	611	0.94
NICU9-A50	III	China	SRR9316770	50124720	49859102	149	7086640005	0.95	12.80	0.45	468	0.94
NSICU1-A24	III	China	SRR9316751	7438002	7437206	149	1061214504	0.95	0.04	0.45	82	0.94
NY-WCPHL-CAU-006553-0	III	United States	SRR24422757	13606552	13103054	146	1641104951	0.88	2.36	0.44	133	0.94
NY-WCPHL-CAU-052664	III	United States	SRR24621174	12665722	12224970	144	1543361767	0.89	1.75	0.44	128	0.94
NY-WCPHL-CAU-055098	III	United States	SRR24422751	14674824	14106570	146	1775912341	0.88	2.10	0.44	146	0.94
NY-WCPHL-CAU-244146	III	United States	SRR24621161	12968484	12536054	144	1601663568	0.90	2.28	0.44	127	0.94
Patient-001	III	United Kingdom	SRR7976549	5048992	5011700	145	621574784	0.85	0.34	0.46	56	0.93
Patient-002	III	United Kingdom	SRR7976579	4212246	4169966	145	528835807	0.87	0.37	0.45	47	0.93
Patient-003	III	United Kingdom	SRR7976616	5251580	5236014	149	685899971	0.88	0.40	0.45	61	0.94
Patient-004	III	United Kingdom	SRR7976582	4821338	4780436	145	609178978	0.88	0.43	0.46	54	0.93
Patient-005	III	United Kingdom	SRR7976614	4042730	4029106	149	516763724	0.86	0.34	0.45	45	0.93
Patient-006-01	III	United Kingdom	SRR7976557	4933476	4879120	145	623541661	0.88	0.43	0.46	54	0.93
Patient-007	III	United Kingdom	SRR7976542	2314318	2308292	149	298371623	0.87	0.20	0.45	26	0.92
Patient-008-01	III	United Kingdom	SRR7976584	5530766	5466358	144	694812816	0.88	0.43	0.46	61	0.93
Patient-009-01	III	United Kingdom	SRR7976597	5104848	5031048	143	621159792	0.86	0.39	0.46	54	0.93
Patient-010-01	III	United Kingdom	SRR7976591	5073510	4943838	139	586987535	0.85	0.49	0.46	52	0.93
Patient-011-01	III	United Kingdom	SRR7976577	5918578	5832022	142	704687336	0.85	0.33	0.45	64	0.94
Patient-012-01	III	United Kingdom	SRR7976604	5300534	5210118	142	613970217	0.83	0.31	0.46	56	0.93
Patient-013-01	III	United Kingdom	SRR7976564	3913634	3855204	143	478850837	0.87	0.34	0.46	42	0.93
Patient-015-01	III	United Kingdom	SRR7976599	3856626	3818610	145	463138062	0.83	0.27	0.46	42	0.93
Patient-016	III	United Kingdom	SRR7976578	4578032	4373868	144	550355827	0.87	0.37	0.45	48	0.93
Patient-017	III	United Kingdom	SRR7976540	4546888	4353536	145	553457873	0.88	0.36	0.46	49	0.93
Patient-019-01	III	United Kingdom	SRR7976552	4177048	4137578	145	529531273	0.88	0.39	0.46	46	0.93
Patient-021-01	III	United Kingdom	SRR7976548	4392572	4348400	145	546882616	0.87	0.44	0.46	48	0.93
Patient-022-01	III	United Kingdom	SRR7976572	3305388	3156022	142	390560167	0.87	0.31	0.46	34	0.93
Patient-023	III	United Kingdom	SRR7976553	4085676	4039718	144	494877936	0.85	0.29	0.46	45	0.93
Patient-024-01	III	United Kingdom	SRR7976592	3694968	3657334	144	453506148	0.86	0.36	0.46	40	0.93
Patient-025-01	III	United Kingdom	SRR7976565	4793220	4752564	146	588516344	0.85	0.36	0.46	53	0.93
Patient-026	III	United Kingdom	SRR7976600	4051122	3995702	144	499188192	0.87	0.36	0.46	44	0.93
Patient-027-01	III	United Kingdom	SRR7976615	4248334	4189174	144	521052910	0.86	0.35	0.46	46	0.93
Patient-028-01	III	United Kingdom	SRR7976585	3634274	3566100	139	427235239	0.86	0.35	0.46	38	0.93
Patient-029-01	III	United Kingdom	SRR7976558	4042852	4012486	145	498296479	0.85	0.36	0.46	44	0.93
Patient-030	III	United Kingdom	SRR7976598	4083500	4019326	143	499847990	0.87	0.39	0.46	44	0.93
Patient-031	III	United Kingdom	SRR7976607	5315254	5273532	145	680118311	0.89	0.53	0.46	59	0.94
Patient-032	III	United Kingdom	SRR7976580	5230320	5175498	144	656490167	0.88	0.49	0.46	57	0.94
Patient-033-01	III	United Kingdom	SRR7976541	5185868	5138346	145	652447558	0.88	0.58	0.46	56	0.93
Patient-034	III	United Kingdom	SRR7976556	4743022	4704792	145	575478959	0.84	0.36	0.46	52	0.93
Patient-037	III	United Kingdom	SRR7976603	4850014	4808456	145	619317125	0.89	0.48	0.46	54	0.93
Patient-038	III	United Kingdom	SRR7976545	3436700	3401858	145	416624506	0.84	0.27	0.46	38	0.93
RI-A112	III	China	SRR9316720	65152332	64796182	149	9059402876	0.93	11.02	0.45	619	0.94
RICU1-A1	III	China	SRR9316788	7783360	7782962	149	1050089824	0.90	0.04	0.44	81	0.94
RICU10-A57	III	China	SRR9316775	9178314	9176858	149	1307009139	0.95	0.05	0.45	97	0.94
RICU11-A62	III	China	SRR9316784	57857550	57201048	149	7868167557	0.92	10.39	0.44	511	0.94
RICU12-A80	III	China	SRR9316798	6813946	6813154	149	942639171	0.92	0.04	0.45	72	0.94
RICU13-A83	III	China	SRR9316810	7614868	7614062	149	1085604369	0.95	0.04	0.45	84	0.94
RICU14-A95	III	China	SRR9316816	52212604	51970826	149	7175927020	0.92	8.86	0.45	506	0.94
RICU15-A105	III	China	SRR9316795	51860840	51587330	149	7306335413	0.94	13.19	0.45	487	0.94
RICU16-A106	III	China	SRR9316796	61173290	60833504	149	8625873528	0.95	12.48	0.45	579	0.94
RICU17-A108	III	China	SRR9316724	9194900	9193802	149	1303175585	0.95	0.05	0.45	98	0.94
RICU18-A113	III	China	SRR9316721	104991484	104535600	149	1,4852E+10	0.95	11.43	0.45	1022	0.94
RICU19-A115	III	China	SRR9316723	61242170	60965602	149	8686838144	0.95	12.29	0.45	582	0.94
RICU2-A2	III	China	SRR9316744	8409762	8409030	149	1163694448	0.92	0.05	0.44	88	0.94
RICU3-A3	III	China	SRR9316754	51127352	50851830	149	7214183199	0.95	12.37	0.45	481	0.94
RICU4-A7	III	China	SRR9316779	7634616	7633956	149	1052849287	0.92	0.04	0.44	78	0.94
RICU5-A12	III	China	SRR9316729	52662564	52374066	149	7386100553	0.94	11.02	0.45	505	0.94
RICU6-A14	III	China	SRR9316738	44158568	43927866	149	6214865645	0.94	11.48	0.45	424	0.94
RICU7-A22	III	China	SRR9316746	46979472	46734174	149	6616053212	0.94	11.75	0.45	449	0.94

RICU8-A38	III	China	SRR9316765	49620806	49273416	149	6816245067	0.92	9.00	0.45	467	0.94
RICU9-A52	III	China	SRR9316772	6265302	6264492	149	863987836	0.92	0.04	0.45	67	0.94
S1	III	Spain	ERR12321104	23393782	22381986	144	2833307537	0.92	05.09	0.45	222	0.94
S103	III	Spain	ERR12321086	22829290	21933850	146	2819782255	0.91	3.56	0.45	223	0.94
S117	III	Spain	ERR12321098	23159802	22203048	147	2860077035	0.91	3.97	0.44	227	0.93
S127	III	Spain	ERR12321077	24736248	23994100	147	3089518012	0.92	4.79	0.45	246	0.94
S129	III	Spain	ERR12321100	24403114	23706620	147	3063557453	0.92	4.62	0.45	245	0.94
S131	III	Spain	ERR12321108	23765226	22792450	146	2904425476	0.91	4.72	0.45	229	0.94
S132	III	Spain	ERR12321088	23106094	22093032	146	2840878527	0.92	4.29	0.44	224	0.94
S134	III	Spain	ERR12321091	22638990	21769480	146	2794495397	0.91	3.88	0.44	225	0.94
S135	III	Spain	ERR12321105	23798582	22961910	146	2933633234	0.92	05.03	0.44	234	0.94
S138	III	Spain	ERR12321082	23231044	22525628	148	2894456883	0.91	4.92	0.45	228	0.94
S14	III	Spain	ERR12321101	23288564	22450796	147	2878676667	0.91	4.40	0.44	228	0.93
S140	III	Spain	ERR12321094	22621214	21722616	146	2787974568	0.91	3.72	0.45	223	0.94
S141	III	Spain	ERR12321107	29775732	28247786	138	3393469499	0.92	5.67	0.43	242	0.94
S145	III	Spain	ERR12321089	23825210	22945048	146	2957891722	0.92	4.51	0.44	236	0.94
S147	III	Spain	ERR12321093	22601474	21632826	146	2768354598	0.91	3.95	0.44	223	0.94
S150	III	Spain	ERR12321079	23434734	22463960	145	2869648222	0.91	3.72	0.45	230	0.94
S151	III	Spain	ERR12321099	22386682	21538990	146	2765110850	0.91	3.76	0.45	220	0.94
S2	III	Spain	ERR12321090	24927964	23985374	146	3039334055	0.92	5.42	0.44	233	0.93
S28	III	Spain	ERR12321092	22986686	22285854	148	2868047156	0.91	04.04	0.44	229	0.94
S33	III	Spain	ERR12321075	27338016	26408462	146	3412977137	0.92	3.46	0.45	271	0.94
S35	III	Spain	ERR12321102	23787118	23034660	147	2988640234	0.92	3.89	0.45	240	0.93
S4	III	Spain	ERR12321096	23576554	22621856	146	2882839336	0.92	05.09	0.45	227	0.94
S44	III	Spain	ERR12321097	23418960	22649668	147	2904502787	0.91	4.90	0.45	231	0.94
S56	III	Spain	ERR12321081	21926670	20942790	146	2675804555	0.91	3.79	0.44	213	0.94
S62	III	Spain	ERR12321078	21885406	20894752	146	2659535041	0.91	4.32	0.45	211	0.94
S67	III	Spain	ERR12321076	24892528	24167724	148	3112163628	0.91	4.68	0.45	247	0.94
S72	III	Spain	ERR12321103	26314692	25263768	146	3238828096	0.91	3.95	0.44	256	0.94
S75	III	Spain	ERR12321087	21626470	20838396	146	2691284181	0.91	3.39	0.44	214	0.94
S77	III	Spain	ERR12321106	21153566	20394266	147	2605606545	0.91	05.07	0.45	207	0.94
S8	III	Spain	ERR12321085	23191400	22303824	146	2854882559	0.91	4.30	0.45	227	0.94
S81	III	Spain	ERR12321095	24121642	23226498	147	2979177390	0.91	4.35	0.45	236	0.94
S82	III	Spain	ERR12321084	23507262	22562144	145	2840717468	0.92	5.23	0.43	207	0.94
S86	III	Spain	ERR12321080	24193910	23473426	148	2999716248	0.91	5.12	0.45	235	0.94
S94	III	Spain	ERR12321074	21717672	20940200	147	2704453176	0.91	3.89	0.44	214	0.94
S99	III	Spain	ERR12321104	21740712	20948492	147	2704116698	0.91	3.48	0.44	216	0.94
SICU1-A66	III	China	SRR9316780	6074794	6074336	149	850300574	0.93	0.04	0.45	68	0.94
SICU2-A89	III	China	SRR9316815	8069166	8068726	149	1073316535	0.89	0.05	0.44	83	0.94
TempProbe1	III	United Kingdom	SRR7976593	4711416	4671060	146	595579656	0.87	0.49	0.46	52	0.93
TempProbe2	III	United Kingdom	SRR7976601	2975616	2871678	136	339432047	0.87	0.59	0.46	29	0.92
TempProbe3	III	United Kingdom	SRR7976602	4762642	4722654	146	602059968	0.87	0.47	0.46	53	0.93
TempProbe4	III	United Kingdom	SRR7976571	5328642	5275290	146	669323940	0.87	0.50	0.46	59	0.93
TempProbe5	III	United Kingdom	SRR7976560	3994730	3956742	146	499158623	0.86	0.46	0.46	44	0.93
UCLA-A1	III	United States	SRR12916694	6221628	6152704	205	1208371012	0.95	1.30	0.45	94	0.94
UCLA-A2	III	United States	SRR12916693	3591948	3565040	231	791156523	0.96	0.63	0.45	63	0.94
UCLA-C1	III	United States	SRR12916692	2444958	2428330	236	547854948	0.96	0.49	0.45	43	0.94
UCLA-D1	III	United States	SRR12916691	2472510	2453800	235	552696737	0.96	0.47	0.45	43	0.94
WM-18-176	III	Australia	SRR11485318	8317892	7751286	131	894614897	0.89	0.54	0.45	74	0.94
WM-18-177	III	South Africa	SRR11485317	6361744	6043140	142	701193049	0.82	0.63	0.45	63	0.93
WM-18-178	III	South Africa	SRR11485332	8668912	8081792	129	919896183	0.90	0.69	0.45	76	0.94
WM-18-179	III	South Africa	SRR11485331	8134268	7565112	130	866076761	0.89	0.55	0.45	69	0.94
WM-18-180	III	Australia	SRR11485320	10129930	9390944	129	1062902246	0.89	0.60	0.45	86	0.94
WM-18-181	III	Australia	SRR11485334	8477836	7862120	131	900770319	0.89	0.54	0.45	73	0.94
WM-18-182	III	Australia	SRR11485333	7850380	7302744	131	833020869	0.88	0.45	0.45	64	0.94
WM-18-187	III	Australia	SRR11485324	9638072	8948870	130	1021143731	0.89	0.60	0.45	82	0.94
WM-18-188	III	Australia	SRR11485323	7233368	6740344	133	778097557	0.89	0.44	0.45	60	0.94
WM-18-189	III	Australia	SRR11485322	7129758	6644248	131	760178758	0.88	0.36	0.45	60	0.94
WM-18-190	III	Australia	SRR11485321	9167256	8572078	132	988616369	0.89	0.58	0.45	75	0.94
WM-18-197	III	Australia	SRR11485319	8761846	8135790	129	927640701	0.89	0.55	0.45	75	0.93

ACEGID-C2	IV	Nigeria	SRR19214214	7705334	7553106	139	988548891	0.95	0.96	0.45	77	0.93
ACEGID-C3	IV	Nigeria	SRR19214213	9804272	9620928	140	1258115609	0.94	01.09	0.45	99	0.93
B11892	IV	Israel	SRR10461259	3502988	3427736	242	758875163	0.93	0.06	0.45	59	0.93
B11896	IV	Israel	SRR10461255	4770054	4728548	244	1058299227	0.93	0.06	0.45	86	0.93
B11897	IV	Israel	SRR10461254	3437498	3419366	244	762693833	0.92	0.13	0.44	59	0.93
B12094	IV	Panama	SRR10461252	5052962	5004954	243	1107027100	0.93	0.71	0.45	61	0.93
B12101	IV	Panama	SRR10461183	4529188	4475042	243	979086995	0.92	0.45	0.46	52	0.93
B12106	IV	Panama	SRR10461179	5619656	5542942	241	1217796444	0.93	0.36	0.45	80	0.93
B12186	IV	Venezuela	SRR10461195	4719298	4684174	243	1034174421	0.92	0.50	0.47	31	0.93
B12187	IV	Venezuela	SRR10461194	6737768	6689722	243	1484312308	0.93	0.32	0.45	98	0.93
B12188	IV	Venezuela	SRR10461193	4896210	4856840	243	1068822626	0.92	0.53	0.47	47	0.93
B12244	IV	Colombia	SRR10461192	7133694	7086562	238	1530374610	0.92	0.22	0.44	116	0.93
B12279	IV	Colombia	SRR10461189	7311872	7261758	243	1571588554	0.91	0.43	0.43	119	0.93
B12284	IV	Colombia	SRR10461188	6119420	6073528	242	1304925683	0.90	0.29	0.44	102	0.93
B12388	IV	United States	SRR7909221	3067686	3055002	244	690005451	0.93	0.11	0.44	54	0.93
B12406	IV	United States	SRR7909405	3473412	3442718	243	705206090	0.88	0.07	0.43	55	0.93
B12847	IV	United States	SRR12784142	5511764	5479198	242	1158441250	0.90	0.04	0.45	99	0.93
B12847	IV	United States	SRR7909414	5511764	5479198	242	1158440917	0.90	0.04	0.45	99	0.93
B12938	IV	United States	SRR7909357	3596234	3510896	234	723043502	0.83	0.02	0.43	57	0.93
B13699	IV	United States	SRR12784141	4812766	4778366	246	1057772819	0.90	0.04	0.45	87	0.93
B13700	IV	United States	SRR12784130	4389698	4362868	246	965048594	0.90	0.05	0.44	77	0.93
B13701	IV	United States	SRR12784125	4742700	4705604	246	1040951020	0.90	0.05	0.44	83	0.93
B13702	IV	United States	SRR12784124	3468654	3446044	246	755094822	0.89	0.06	0.44	60	0.93
B13703	IV	United States	SRR12073466	3900968	3873124	246	838954426	0.89	0.04	0.44	69	0.93
B16329	IV	United States	SRR12073454	11926350	11903816	244	2562015668	0.89	0.23	0.44	206	0.93
GCA 003014415.1	IV	Venezuela	Reference assembly								79	0.93
MRL_2723	IV	South Africa	SRR14802837	5602388	5403920	267	1202665603	0.85	0.23	0.47	105	0.92
MRL_353	IV	South Africa	SRR14802929	5027614	4770856	281	997652140	0.78	0.07	0.46	75	0.91
MRL_4916	IV	South Africa	SRR14802909	8767114	8548406	283	2032201763	0.85	0.32	0.47	156	0.93
N19-00-135	IV	Canada	SRR15192303	10001624	9209232	146	1114055579	0.83	09.03	0.45	65	0.93
NG-20724Am-01	IV	Netherlands	SRR9993948	4846302	4573386	150	570806907	0.83	5.27	0.44	23	0.91
IFRC2087	V	Iran	SRR9007776	5765944	5552270	149	708971982	0.85	04.03	0.44	27	0.92

Supplementary Table 2. Metadata (sample origin, phenotypic and genotypic characteristics of resistance-related mutations) of all *Candida auris* analyzed in this study (n=689).

Sample ID	Country	Phylogenetic clade	Collection date	Collection year	Fluconazole (FLC)	Amphotericin B (AMB)	Micafungin (MFG)	Specimen source	Specimen origin	SRA run	ERG11 (FLC)	FKS1 (MFG)
3001524381	Canada	I	2018	2018	Resistant	Resistant	Susceptible	Axilla/Groin swab	Clinical	SRR10554762	K143R	WT
15B10	United Kingdom	I	2015-12-28	2015	Resistant	Resistant	Susceptible	Groin swab	Clinical	ERR2299870	Y132F	WT
16B22a	United Kingdom	I	2016-02-27	2016	Resistant	Susceptible	Susceptible	Axilla swab	Clinical	ERR2299881	Y132F	WT
16I34	United Kingdom	I	2016-10-23	2016	Resistant	Susceptible	Susceptible	Axilla swab	Clinical	ERR2299892	Y132F	WT
ACEGID-C1	Nigeria	I	2021-02	2021	Unknown	Unknown	Unknown	blood	Clinical	SRR19214215	Y132F	WT
ACEGID-C4	Nigeria	I	2021-04	2021	Unknown	Unknown	Unknown	blood	Clinical	SRR19214212	Y132F	WT
B11115	Pakistan	I	2015-03-23	2015	Resistant	Resistant	Susceptible	Urine	Clinical	SRR3883429	K143R	WT
B11116	Pakistan	I	2015-03-23	2015	Resistant	Resistant	Susceptible	Urine	Clinical	SRR3883430	K143R	WT
B11117	Pakistan	I	2015-03-27	2015	Resistant	Resistant	Susceptible	Blood	Clinical	SRR3883431	Y132F	WT
B11214	India	I	2014-07-15	2014	Resistant	Susceptible	Susceptible	Blood	Clinical	SRR3883445	Y132F	WT
B11215	India	I	2014-07-15	2014	Resistant	Susceptible	Susceptible	Blood	Clinical	SRR3883446	Y132F	WT
B11218	India	I	2014-04-15	2014	Resistant	Susceptible	Resistant	Blood	Clinical	SRR3883450	Y132F	WT
B13343	United States	I	2017-06-22	2017	Resistant	Resistant	Susceptible	Wound	Clinical	SRR7909249	Y132F	WT
B13464	Canada	I	2017-05-15	2017	Resistant	Susceptible	Susceptible	Ear fluid	Clinical	SRR10461158	K143R	WT
B13520	United States	I	2017-07-15	2017	Resistant	Susceptible	Susceptible	Fluid Drainage	Clinical	SRR7909394	K143R	WT
B13696	United States	I	2017-08-13	2017	Resistant	Susceptible	Resistant	Urine	Clinical	SRR7909346	Y132F	S639Y
B13916	United Arab Emirates	I	2017-09-11	2017	Resistant	Resistant	Resistant	Blood	Clinical	SRR10461157	Y132F	S639F
B13917	United Arab Emirates	I	2017-09-15	2017	Resistant	Susceptible	Susceptible	Blood	Clinical	SRR10461156	K143R	WT
B14146	Saudi Arabia	I	2017-12-10	2017	Resistant	Susceptible	Susceptible	Blood	Clinical	SRR10461152	K143R	WT
B14147	Saudi Arabia	I	2017-12-25	2017	Resistant	Susceptible	Susceptible	Blood	Clinical	SRR10461151	K143R	WT
B14165	Saudi Arabia	I	2018-01-10	2018	Resistant	Susceptible	Susceptible	Urine	Clinical	SRR10461149	K143R	WT
B14189	United Arab Emirates	I	2018-02-04	2018	Resistant	Susceptible	Susceptible	Unknown	Unknown	SRR10461148	Y132F	WT
B16401	Kenya	I	2011-11-08	2011	Resistant	Susceptible	Susceptible	Blood	Clinical	SRR10852068	Y132F	WT
B16421	Kenya	I	2018-01-25	2018	Unknown	Unknown	Unknown	blood	Clinical	SRR10461140	Y132F	WT
B16422	Kenya	I	2018-04-20	2018	Resistant	Resistant	Susceptible	P/swab	Clinical	SRR10461139	Y132F	WT
B16438	Kenya	I	2014-02-05	2014	Resistant	Resistant	Susceptible	CVC	Clinical	SRR10461241	Y132F	WT
B16444	Kenya	I	2014-02-25	2014	Resistant	Resistant	Resistant	urine	Clinical	SRR10461237	Y132F	S639F
B16448	Kenya	I	2014-04-02	2014	Resistant	Resistant	Resistant	urine	Clinical	SRR10461235	Y132F	S639F
B16472	Kenya	I	2015-07-04	2015	Resistant	Resistant	Susceptible	urine	Clinical	SRR10461226	Y132F	WT
B16477	Kenya	I	2015-11-15	2015	Resistant	Resistant	Susceptible	blood	Clinical	SRR10461224	Y132F	WT
B16479	Kenya	I	2016-02-08	2016	Resistant	Resistant	Susceptible	blood	Clinical	SRR10461223	Y132F	WT
B16487	Kenya	I	2016-10-11	2016	Resistant	Resistant	Susceptible	Ascitic Fluid	Clinical	SRR10461218	Y132F	WT
B16490	Kenya	I	2017-02-02	2017	Resistant	Resistant	Susceptible	blood	Clinical	SRR10461217	Y132F	WT
B16503	Kenya	I	2017-05-21	2017	Resistant	Resistant	Susceptible	Swab	Clinical	SRR10461214	Y132F	WT
B16510	Kenya	I	2017-04-21	2017	Resistant	Resistant	Resistant	Urine	Clinical	SRR10461210	Y132F	S639F
B16511	Kenya	I	2017-05-28	2017	Resistant	Resistant	Susceptible	blood	Clinical	SRR10461209	Y132F	WT
B16513	Kenya	I	2017-06-16	2017	Resistant	Resistant	Susceptible	CSF	Clinical	SRR10461208	Y132F	WT
B17051	United States	I	2019	2019	Unknown	Unknown	Unknown	urine	Clinical	SRR12073470	K143R	WT
B17055	United States	I	2019	2019	Unknown	Unknown	Unknown	urine	Clinical	SRR12073468	K143R	WT
B17604	United States	I	2019	2019	Unknown	Unknown	Unknown	blood	Clinical	SRR12073467	K143R	WT
B17650	United States	I	2019	2019	Unknown	Unknown	Unknown	urine	Clinical	SRR12073464	K143R	WT
B18978	United States	I	2020	2020	Unknown	Unknown	Unknown	blood	Clinical	SRR12787995	Y132F	WT
B18979	United States	I	2020	2020	Unknown	Unknown	Unknown	blood	Clinical	SRR12787994	Y132F	WT
B19130	United States	I	2020	2020	Unknown	Unknown	Unknown	urine	Clinical	SRR12784132	Y132F	WT
Cau1	Austria	I	2018-01	2018	Susceptible	Resistant	Susceptible	Auditory canal	Clinical	SRR23080879	WT	WT
Cau2	Austria	I	2020-02	2020	Susceptible	Resistant	Susceptible	Auditory canal	Clinical	SRR23080878	WT	WT
Cau3	Austria	I	2020-05	2020	Resistant	Resistant	Susceptible	Urinary tract	Clinical	SRR23080877	Y132F	WT
Cau5	Austria	I	2022-04	2022	Resistant	Resistant	Susceptible	Urinary tract	Clinical	SRR23080875	Y132F	WT
CAU924-6920	Egypt	I	2017-01	2017	Unknown	Unknown	Unknown	blood	Clinical	SRR13402267	Y132F	WT
CNRMA15-337	France	I	2015-05-15	2015	Resistant	Susceptible	Susceptible	Blood	Clinical	SRR10723348	Y132F	WT
CNRMA17-624	France	I	2017-08-15	2017	Resistant	Susceptible	Susceptible	Throat	Clinical	SRR10723347	Y132F	WT
CNRMA21-252	France	I	2021-04-03	2021	Susceptible	Susceptible	Susceptible	Burn wound	Clinical	SRR20823034	Y132F	WT
CNRMA21-86	France	I	2021-02-06	2021	Susceptible	Susceptible	Susceptible	BAL	Clinical	SRR20823037	Y132F	WT
CNRMA21-87	France	I	2021-02-07	2021	Susceptible	Susceptible	Susceptible	Catheter	Clinical	SRR20823036	Y132F	WT
CNRMA21-88	France	I	2021-02-02	2021	Susceptible	Susceptible	Susceptible	Burn wound	Clinical	SRR20823035	Y132F	WT
GCA 002759435.2	Pakistan	I	2008-11-05	2008	Susceptible	Susceptible	Susceptible	Blood	Clinical	Reference assembly	WT	WT
MRL 128	South Africa	I	2018	2018	Unknown	Unknown	Unknown	Urine	Clinical	SRR14802847	Y132F	WT
MRL 3732	South Africa	I	2017	2017	Unknown	Unknown	Unknown	Blood	Clinical	SRR14802946	Y132F	WT
MRL 3775	South Africa	I	2017	2017	Unknown	Unknown	Unknown	Blood	Clinical	SRR14802945	Y132F	D642Y
MRL 4000	South Africa	I	2017	2017	Unknown	Unknown	Unknown	Blood	Clinical	SRR14802844	Y132F	WT
MRL 4288	South Africa	I	2017	2017	Unknown	Unknown	Unknown	Blood	Clinical	SRR14802833	Y132F	WT
MRL 4645	South Africa	I	2017	2017	Unknown	Unknown	Unknown	Blood	Clinical	SRR14802930	Y132F	WT
MRL 4934	South Africa	I	2017	2017	Unknown	Unknown	Unknown	Blood	Clinical	SRR14802903	Y132F	WT
MRL 5116	South Africa	I	2017	2017	Unknown	Unknown	Unknown	Blood	Clinical	SRR14802898	Y132F	WT
MRL 5173	South Africa	I	2017	2017	Unknown	Unknown	Unknown	Blood	Clinical	SRR14802896	Y132F	WT
MRL 5233	South Africa	I	2017	2017	Unknown	Unknown	Unknown	Blood	Clinical	SRR14802894	Y132F	D642Y
MRL 5425	South Africa	I	2017	2017	Unknown	Unknown	Unknown	Blood	Clinical	SRR14802919	Y132F	WT
MRL 5588	South Africa	I	2017	2017	Unknown	Unknown	Unknown	Blood	Clinical	SRR14802908	Y132F	WT
MRL 5778	South Africa	I	2017	2017	Unknown	Unknown	Unknown	Blood	Clinical	SRR14802851	Y132F	WT
MRL 6196	South Africa	I	2017	2017	Unknown	Unknown	Unknown	Blood	Clinical	SRR14802848	Y132F	WT
N18-01-797	Canada	I	2018	2018	Resistant	Resistant	Susceptible	Axilla/Groin swab	Clinical	SRR15192317	Y132F	WT
N18-01-802	Canada	I	2018	2018	Resistant	Resistant	Susceptible	Drainage	Clinical	SRR15192318	Y132F	WT
N18-01-914	Canada	I	2018	2018	Resistant	Resistant	Susceptible	Axilla/Groin swab	Clinical	SRR13362217	Y132F	WT
N18-02-487	Canada	I	2017	2017	Resistant	Resistant	Susceptible	Ear	Clinical	SRR15192314	K143R	WT
N18-02-621	Canada	I	2018	2018	Resistant	Resistant	Susceptible	Axilla/Groin swab	Clinical	SRR10554763	K143R	WT
N19-02-669	Canada	I	2019	2019	Resistant	Resistant	Susceptible	Nares	Clinical	SRR15192309	K143R	WT
N19-03-893	Canada	I	2019	2019	Resistant	Resistant	Susceptible	Toe	Clinical	SRR15192315	Y132F	WT

NRZ-2015-214	Germany	I	2015-10-11	2015	Resistant	Unknown	Unknown	blood	Clinical	SRR10292114	Y132F	WT
NRZ-2017-288	Germany	I	2017-07-25	2017	Resistant	Unknown	Unknown	Urine	Clinical	SRR10292311	Y132F	WT
NRZ-2017-367	Germany	I	2017-09-04	2017	Resistant	Unknown	Unknown	Tissue	Clinical	SRR10292063	K143R	WT
NRZ-2017-394-1	Germany	I	2017-09-11	2017	Resistant	Unknown	Unknown	Urine	Clinical	SRR10277313	Y132F	WT
RCPF-1821	Russia	I	2017-10-27	2017	Unknown	Unknown	Unknown	blood	Clinical	SRR9201318	K143R	WT
B11808	South Korea	II	2004	2004	Susceptible	Susceptible	Susceptible	Auditory canal	Clinical	SRR9645764	WT	WT
B11809	South Korea	II	2004	2004	Resistant	Susceptible	Susceptible	Auditory canal	Clinical	SRR9645762	WT	WT
B12043	United States	II	2016-08-03	2016	Susceptible	Susceptible	Susceptible	Ear	Clinical	SRR7909185	WT	WT
B12081	United States	II	2016-10-19	2016	Susceptible	Susceptible	Susceptible	Ear	Clinical	SRR7909356	WT	WT
B13463	Canada	II	2014-04-07	2014	Susceptible	Susceptible	Susceptible	Ear fluid	Clinical	SRR10461159	WT	WT
B14308	United States	II	2018-02-14	2018	Susceptible	Susceptible	Susceptible	Wound	Clinical	SRR10461147	WT	WT
GCA 003013715.2	Japan	II	2009	2009	Susceptible	Susceptible	Unknown	Auditory canal	Clinical	Reference assembly	WT	WT
N18-02-485	Canada	II	2014	2014	Susceptible	Susceptible	Susceptible	Ear	Clinical	SRR15192319	WT	WT
N19-02-739	Canada	II	2019	2019	Susceptible	Susceptible	Susceptible	Ear	Clinical	SRR15192310	WT	WT
N19-03-592	Canada	II	2019	2019	Susceptible	Susceptible	Susceptible	Ear	Clinical	SRR15192311	WT	WT
AA-194	Spain	III	2016-06-28	2016	Resistant	Susceptible	Susceptible	Blood	Clinical	SRR10461267	VF125AL	WT
AA-200	Spain	III	2016-08-28	2016	Resistant	Susceptible	Susceptible	Blood	Clinical	SRR10461266	VF125AL	WT
AA-214	Spain	III	2016-11-15	2016	Resistant	Susceptible	Susceptible	Blood	Clinical	SRR10461265	VF125AL	WT
B11222	South Africa	III	2012-10-15	2012	Resistant	Susceptible	Susceptible	Blood	Clinical	SRR3883454	VF125AL	WT
B11223	South Africa	III	2013-01-15	2013	Resistant	Susceptible	Susceptible	Blood	Clinical	SRR3883455	VF125AL	WT
B11224	South Africa	III	2013-10-12	2013	Resistant	Susceptible	Susceptible	Blood	Clinical	SRR3883456	VF125AL	WT
B11225	South Africa	III	2014-04-20	2014	Resistant	Susceptible	Susceptible	Urine	Clinical	SRR3883457	VF125AL	WT
B11226	South Africa	III	2014-03-09	2014	Resistant	Susceptible	Susceptible	Right leg	Clinical	SRR3883458	VF125AL	WT
B11227	South Africa	III	2014-03-16	2014	Resistant	Susceptible	Susceptible	Blood	Clinical	SRR3883459	VF125AL	WT
B11228	South Africa	III	2014-03-18	2014	Resistant	Susceptible	Susceptible	Hip tissue	Clinical	SRR3883461	VF125AL	WT
B11229	South Africa	III	2014-04-21	2014	Resistant	Susceptible	Susceptible	Urine (catheter)	Clinical	SRR3883462	VF125AL	WT
B11230	South Africa	III	2014-03-13	2014	Resistant	Susceptible	Susceptible	Urine	Clinical	SRR3883463	WT	WT
B12037	Canada	III	2012-10-18	2012	Susceptible	Susceptible	Susceptible	Ear fluid	Clinical	SRR10461253	WT	WT
B12631	United States	III	2017-03-16	2017	Resistant	Susceptible	Susceptible	Wound	Clinical	SRR7909359	VF125AL	WT
B16404	Kenya	III	2017-10-16	2017	Resistant	Susceptible	Resistant	Urine	Clinical	SRR10461146	VF125AL	F635dup
B16406	Kenya	III	2017-10-30	2017	Resistant	Susceptible	Resistant	Urine	Clinical	SRR10461145	VF125AL	S639F
B16410	Kenya	III	2018-06-15	2018	Resistant	Susceptible	Susceptible	Blood	Clinical	SRR10461144	VF125AL	WT
B16415	Kenya	III	2018-07-16	2018	Resistant	Susceptible	Susceptible	Blood	Clinical	SRR10461143	VF125AL	WT
B16417	Kenya	III	2018-06-26	2018	Resistant	Susceptible	Susceptible	Blood	Clinical	SRR10461142	VF125AL	WT
B16419	Kenya	III	2018-04-30	2018	Resistant	Susceptible	Susceptible	Blood	Clinical	SRR10461141	VF125AL	WT
B16424	Kenya	III	2018-05-16	2018	Resistant	Susceptible	Susceptible	Blood	Clinical	SRR10461138	VF125AL	WT
B16425	Kenya	III	2011-11-08	2011	Unknown	Unknown	Unknown	blood	Clinical	SRR10461245	VF125AL	WT
B16431	Kenya	III	2013-10-10	2013	Resistant	Susceptible	Susceptible	Blood	Clinical	SRR10461244	VF125AL	WT
B16432	Kenya	III	2013-12-17	2013	Resistant	Susceptible	Susceptible	Blood	Clinical	SRR10461211	VF125AL	WT
B16433	Kenya	III	2013-12-17	2013	Resistant	Susceptible	Susceptible	Blood	Clinical	SRR10461243	VF125AL	WT
B16436	Kenya	III	2014-01-21	2014	Resistant	Susceptible	Susceptible	Blood	Clinical	SRR10461242	VF125AL	WT
B16439	Kenya	III	2014-02-11	2014	Resistant	Susceptible	Susceptible	Blood	Clinical	SRR10461240	VF125AL	WT
B16440	Kenya	III	2014-02-16	2014	Resistant	Susceptible	Resistant	Urine	Clinical	SRR10461239	VF125AL	S639Y
B16441	Kenya	III	2013-10-22	2013	Resistant	Susceptible	Susceptible	Pleural	Clinical	SRR10461238	VF125AL	WT
B16445	Kenya	III	2014-03-21	2014	Resistant	Susceptible	Susceptible	Blood	Clinical	SRR10461236	VF125AL	WT
B16451	Kenya	III	2014-04-07	2014	Resistant	Susceptible	Susceptible	Blood	Clinical	SRR10461234	VF125AL	WT
B16454	Kenya	III	2014-04-15	2014	Resistant	Susceptible	Susceptible	Vascular tip	Clinical	SRR10461233	VF125AL	WT
B16457	Kenya	III	2014-05-22	2014	Resistant	Susceptible	Susceptible	Blood	Clinical	SRR10461232	VF125AL	WT
B16459	Kenya	III	2014-08-16	2014	Resistant	Susceptible	Susceptible	Blood	Clinical	SRR10461231	VF125AL	WT
B16461	Kenya	III	2014-10-08	2014	Resistant	Susceptible	Susceptible	Blood	Clinical	SRR10461230	VF125AL	WT
B16466	Kenya	III	2015-01-21	2015	Resistant	Susceptible	Susceptible	Blood	Clinical	SRR10461229	VF125AL	WT
B16467	Kenya	III	2015-02-02	2015	Resistant	Susceptible	Susceptible	Blood	Clinical	SRR10461228	VF125AL	WT
B16469	Kenya	III	2015-02-09	2015	Resistant	Susceptible	Susceptible	Blood	Clinical	SRR10461227	VF125AL	WT
B16473	Kenya	III	2015-07-27	2015	Resistant	Susceptible	Susceptible	Pleural	Clinical	SRR10461225	VF125AL	WT
B16481	Kenya	III	2016-02-27	2016	Resistant	Susceptible	Susceptible	Blood	Clinical	SRR10461222	VF125AL	WT
B16482	Kenya	III	2016-03-02	2016	Resistant	Susceptible	Susceptible	Blood	Clinical	SRR10461221	VF125AL	WT
B16484	Kenya	III	2016-03-21	2016	Resistant	Susceptible	Resistant	Urine	Clinical	SRR10461220	VF125AL	WT
B16485	Kenya	III	2013-11-05	2013	Resistant	Susceptible	Susceptible	Blood	Clinical	SRR10461219	VF125AL	WT
B16491	Kenya	III	2017-10-02	2017	Resistant	Susceptible	Susceptible	Urine	Clinical	SRR10461216	VF125AL	F635dup
B16496	Kenya	III	2013-11-05	2013	Resistant	Susceptible	Susceptible	Blood	Clinical	SRR10461215	VF125AL	WT
B16504	Kenya	III	2017-10-23	2017	Resistant	Susceptible	Susceptible	Urine	Clinical	SRR10461213	VF125AL	WT
B16507	Kenya	III	2013-12-14	2013	Resistant	Susceptible	Susceptible	Blood	Clinical	SRR10461212	VF125AL	WT
B16514	Kenya	III	2017-07-05	2017	Resistant	Susceptible	Susceptible	Blood	Clinical	SRR10461207	VF125AL	WT
B16519	Kenya	III	2018-03-07	2018	Resistant	Susceptible	Susceptible	Blood	Clinical	SRR10461205	VF125AL	WT
B16820	United States	III	2018	2018	Unknown	Unknown	Unknown	urine	Clinical	SRR12073443	VF125AL	WT
B17018	United States	III	2019	2019	Unknown	Unknown	Unknown	blood	Clinical	SRR12073482	VF125AL	WT
B17050	United States	III	2018	2018	Unknown	Unknown	Unknown	urine	Clinical	SRR12073471	VF125AL	WT
B17054	United States	III	2019	2019	Unknown	Unknown	Unknown	urine	Clinical	SRR12073469	VF125AL	WT
B17651	United States	III	2019	2019	Unknown	Unknown	Unknown	blood	Clinical	SRR12073463	VF125AL	WT
B17653	United States	III	2019	2019	Unknown	Unknown	Unknown	axilla and groin	Clinical	SRR12073462	VF125AL	WT
B17654	United States	III	2019	2019	Unknown	Unknown	Unknown	axilla and groin	Clinical	SRR12073461	VF125AL	WT
B17655	United States	III	2019	2019	Unknown	Unknown	Unknown	axilla and groin	Clinical	SRR12073460	VF125AL	WT
B17656	United States	III	2019	2019	Unknown	Unknown	Unknown	axilla and groin	Clinical	SRR12073459	VF125AL	WT
B17721	United States	III	2019-02-17	2019	Unknown	Unknown	Unknown	urine	Clinical	SRR17577117	VF125AL	WT
B17741	United States	III	2019-03-14	2019	Unknown	Unknown	Unknown	axilla and groin	Clinical	SRR17577116	VF125AL	WT
B17742	United States	III	2019-03-14	2019	Unknown	Unknown	Unknown	axilla and groin	Clinical	SRR17577105	VF125AL	WT
B17743	United States	III	2019-03-14	2019	Unknown	Unknown	Unknown	axilla and groin	Clinical	SRR17577094	VF125AL	WT
B17746	United States	III	2019-03-14	2019	Unknown	Unknown	Unknown	axilla and groin	Clinical	SRR17577083	VF125AL	WT
B17798	United States	III	2019	2019	Unknown	Unknown	Unknown	blood	Clinical	SRR12073458	VF125AL	WT

B17806	United States	III	2019-03-28	2019	Unknown	Unknown	Unknown	Unknown	Unknown	SRR17577060	VF125AL	WT
B17833	United States	III	2019-04-22	2019	Unknown	Unknown	Unknown	nose	Clinical	SRR17577049	VF125AL	WT
B17834	United States	III	2019-04-22	2019	Unknown	Unknown	Unknown	nose	Clinical	SRR17577038	VF125AL	WT
B17835	United States	III	2019-04-22	2019	Unknown	Unknown	Unknown	nose	Clinical	SRR14252432	VF125AL	WT
B17853	United States	III	2019-04-03	2019	Unknown	Unknown	Unknown	axilla and groin	Clinical	SRR17577071	VF125AL	WT
B17854	United States	III	2019-04-03	2019	Unknown	Unknown	Unknown	axilla and groin	Clinical	SRR17577070	VF125AL	WT
B17855	United States	III	2019-04-03	2019	Unknown	Unknown	Unknown	axilla and groin	Clinical	SRR17577115	VF125AL	WT
B17856	United States	III	2019-04-04	2019	Unknown	Unknown	Unknown	axilla and groin	Clinical	SRR17577114	VF125AL	WT
B17857	United States	III	2019-04-08	2019	Unknown	Unknown	Unknown	nose	Clinical	SRR17577113	VF125AL	WT
B17858	United States	III	2019-04-08	2019	Unknown	Unknown	Unknown	axilla and groin	Clinical	SRR17577112	VF125AL	WT
B17859	United States	III	2019-04-08	2019	Unknown	Unknown	Unknown	axilla and groin	Clinical	SRR17577111	VF125AL	WT
B17860	United States	III	2019-04-08	2019	Unknown	Unknown	Unknown	axilla and groin	Clinical	SRR17577110	VF125AL	WT
B17861	United States	III	2019-04-08	2019	Unknown	Unknown	Unknown	axilla and groin	Clinical	SRR17577109	VF125AL	WT
B17862	United States	III	2019-04-08	2019	Unknown	Unknown	Unknown	axilla	Clinical	SRR17577108	VF125AL	WT
B17863	United States	III	2019-04-08	2019	Unknown	Unknown	Unknown	nose	Clinical	SRR17577107	VF125AL	WT
B17864	United States	III	2019-04-08	2019	Unknown	Unknown	Unknown	nose	Clinical	SRR17577106	VF125AL	WT
B17865	United States	III	2019-04-08	2019	Unknown	Unknown	Unknown	axilla and groin	Clinical	SRR17577104	VF125AL	WT
B17866	United States	III	2019-04-08	2019	Unknown	Unknown	Unknown	axilla and groin	Clinical	SRR17577103	VF125AL	WT
B17867	United States	III	2019-04-08	2019	Unknown	Unknown	Unknown	axilla	Clinical	SRR17577102	VF125AL	WT
B17868	United States	III	2019-04-08	2019	Unknown	Unknown	Unknown	axilla and groin	Clinical	SRR17577101	VF125AL	WT
B17869	United States	III	2019-04-08	2019	Unknown	Unknown	Unknown	axilla and groin	Clinical	SRR17577100	VF125AL	WT
B17870	United States	III	2019-04-08	2019	Unknown	Unknown	Unknown	axilla and groin	Clinical	SRR17577099	VF125AL	WT
B17871	United States	III	2019-04-11	2019	Unknown	Unknown	Unknown	axilla and groin	Clinical	SRR17577098	VF125AL	WT
B17872	United States	III	2019-04-11	2019	Unknown	Unknown	Unknown	nose	Clinical	SRR17577097	VF125AL	WT
B17873	United States	III	2019-04-03	2019	Unknown	Unknown	Unknown	nose	Clinical	SRR17577096	VF125AL	WT
B17874	United States	III	2019-04-03	2019	Unknown	Unknown	Unknown	nose	Clinical	SRR17577095	VF125AL	WT
B17875	United States	III	2019-05-07	2019	Unknown	Unknown	Unknown	nose	Clinical	SRR17577093	VF125AL	WT
B17876	United States	III	2019-05-07	2019	Unknown	Unknown	Unknown	nose	Clinical	SRR17577092	VF125AL	WT
B17877	United States	III	2019-05-09	2019	Unknown	Unknown	Unknown	nose	Clinical	SRR17577091	VF125AL	WT
B17878	United States	III	2019-05-09	2019	Unknown	Unknown	Unknown	nose	Clinical	SRR17577090	VF125AL	WT
B17904	United States	III	2019	2019	Unknown	Unknown	Unknown	axilla and groin	Clinical	SRR12073457	VF125AL	WT
B17905	United States	III	2019	2019	Unknown	Unknown	Unknown	blood	Clinical	SRR12073456	VF125AL	WT
B17908	United States	III	2019	2019	Unknown	Unknown	Unknown	blood	Clinical	SRR12073453	VF125AL	WT
B17909	United States	III	2019	2019	Unknown	Unknown	Unknown	blood	Clinical	SRR12073452	VF125AL	WT
B17910	United States	III	2019	2019	Unknown	Unknown	Unknown	blood	Clinical	SRR12073451	VF125AL	WT
B17911	United States	III	2019	2019	Unknown	Unknown	Unknown	urine	Clinical	SRR12073450	VF125AL	WT
B17912	United States	III	2019	2019	Unknown	Unknown	Unknown	Unknown	Unknown	SRR12073449	VF125AL	WT
B17913	United States	III	2019	2019	Unknown	Unknown	Unknown	wound	Clinical	SRR12073448	VF125AL	WT
B17914	United States	III	2019	2019	Unknown	Unknown	Unknown	bronchial washings	Clinical	SRR12073447	VF125AL	WT
B17915	United States	III	2019	2019	Unknown	Unknown	Unknown	blood	Clinical	SRR12073446	VF125AL	WT
B17916	United States	III	2019-05-07	2019	Unknown	Unknown	Unknown	bronchial washings	Clinical	SRR17577089	VF125AL	WT
B17924	United States	III	2019-05-04	2019	Unknown	Unknown	Unknown	blood	Clinical	SRR17577088	VF125AL	WT
B17937	United States	III	2019-05-29	2019	Unknown	Unknown	Unknown	axilla and groin	Clinical	SRR17577087	VF125AL	WT
B17948	United States	III	2019-05-15	2019	Unknown	Unknown	Unknown	axilla and groin	Clinical	SRR17577086	VF125AL	WT
B17949	United States	III	2019-05-15	2019	Unknown	Unknown	Unknown	axilla and groin	Clinical	SRR17577085	VF125AL	WT
B17950	United States	III	2019-05-15	2019	Unknown	Unknown	Unknown	axilla and groin	Clinical	SRR17577084	VF125AL	WT
B17979	United States	III	2019	2019	Unknown	Unknown	Unknown	blood	Clinical	SRR12073445	VF125AL	WT
B17980	United States	III	2019	2019	Unknown	Unknown	Unknown	blood	Clinical	SRR12073444	VF125AL	WT
B17981	United States	III	2019	2019	Unknown	Unknown	Unknown	blood	Clinical	SRR12073442	VF125AL	WT
B18157	United States	III	2019-07-04	2019	Unknown	Unknown	Unknown	urine	Clinical	SRR17577082	VF125AL	WT
B18158	United States	III	2019-07-07	2019	Unknown	Unknown	Unknown	foreign object (body)	Clinical	SRR17577069	VF125AL	WT
B18159	United States	III	2019-07-17	2019	Unknown	Unknown	Unknown	axilla and groin	Clinical	SRR17577068	VF125AL	WT
B18160	United States	III	2019-07-15	2019	Unknown	Unknown	Unknown	blood	Clinical	SRR17577067	VF125AL	WT
B18225	United States	III	2019-07-17	2019	Unknown	Unknown	Unknown	urine	Clinical	SRR17577066	VF125AL	WT
B18226	United States	III	2019-07-29	2019	Unknown	Unknown	Unknown	sputum	Clinical	SRR17577065	VF125AL	WT
B18227	United States	III	2019-07-24	2019	Unknown	Unknown	Unknown	urine	Clinical	SRR17577064	VF125AL	WT
B18249	United States	III	2019-08-13	2019	Unknown	Unknown	Unknown	axilla and groin	Clinical	SRR17577063	VF125AL	WT
B18263	United States	III	2019-08-28	2019	Unknown	Unknown	Unknown	axilla and groin	Clinical	SRR17577062	VF125AL	WT
B18278	United States	III	2019-09-11	2019	Unknown	Unknown	Unknown	axilla and groin	Clinical	SRR17577061	VF125AL	WT
B18289	United States	III	2019-08-22	2019	Unknown	Unknown	Unknown	blood	Clinical	SRR17577059	VF125AL	WT
B18458	United States	III	2019-09-25	2019	Unknown	Unknown	Unknown	axilla and groin	Clinical	SRR17577058	VF125AL	WT
B18460	United States	III	2019-09-01	2019	Unknown	Unknown	Unknown	blood	Clinical	SRR17577057	VF125AL	WT
B18461	United States	III	2019-08-17	2019	Unknown	Unknown	Unknown	ear	Clinical	SRR17577056	VF125AL	WT
B18526	United States	III	2019	2019	Unknown	Unknown	Unknown	blood	Clinical	SRR12073441	VF125AL	WT
B18527	United States	III	2019	2019	Unknown	Unknown	Unknown	sputum	Clinical	SRR12073440	VF125AL	WT
B18528	United States	III	2019	2019	Unknown	Unknown	Unknown	blood	Clinical	SRR12073439	VF125AL	WT
B18529	United States	III	2019	2019	Unknown	Unknown	Unknown	blood	Clinical	SRR12073438	VF125AL	WT
B18530	United States	III	2019	2019	Unknown	Unknown	Unknown	aspirate	Clinical	SRR12073437	VF125AL	WT
B18531	United States	III	2019	2019	Unknown	Unknown	Unknown	sputum	Clinical	SRR12073436	VF125AL	WT
B18532	United States	III	2020-07-07	2020	Unknown	Unknown	Unknown	Sputum	Clinical	SRR12073435	VF125AL	WT
B18533	United States	III	2020-07-07	2020	Unknown	Unknown	Unknown	Blood	Clinical	SRR12073484	VF125AL	WT
B18534	United States	III	2019	2019	Unknown	Unknown	Unknown	ulcer	Clinical	SRR12073483	VF125AL	WT
B18540	United States	III	2019-09-15	2019	Unknown	Unknown	Unknown	blood	Clinical	SRR17577055	VF125AL	WT
B18657	United States	III	2019-10-01	2019	Unknown	Unknown	Unknown	axilla and groin	Clinical	SRR17577054	VF125AL	WT
B18665	United States	III	2020-07-07	2020	Unknown	Unknown	Unknown	Axilla/Groin swab	Clinical	SRR12073481	VF125AL	WT
B18666	United States	III	2019	2019	Unknown	Unknown	Unknown	axilla and groin	Clinical	SRR12073480	VF125AL	WT
B18667	United States	III	2019	2019	Unknown	Unknown	Unknown	axilla and groin	Clinical	SRR12073479	VF125AL	WT
B18669	United States	III	2019	2019	Unknown	Unknown	Unknown	axilla and groin	Clinical	SRR12073477	VF125AL	WT
B18683	Kenya	III	2019-11-25	2019	Unknown	Unknown	Unknown	skin swab	Clinical	SRR14252431	VF125AL	WT

B18732	United States	III	2019	2019	Unknown	Unknown	Unknown	axilla and groin	Clinical	SRR12073476	VF125AL	WT
B18733	United States	III	2019	2019	Unknown	Unknown	Unknown	axilla and groin	Clinical	SRR12073475	VF125AL	WT
B18734	United States	III	2019	2019	Unknown	Unknown	Unknown	axilla and groin	Clinical	SRR12073474	VF125AL	WT
B18754	United States	III	2019	2019	Unknown	Unknown	Unknown	fluid body	Clinical	SRR12073473	VF125AL	WT
B18759	United States	III	2019	2019	Unknown	Unknown	Unknown	blood	Clinical	SRR12073472	VF125AL	WT
B18811	United States	III	2020	2020	Unknown	Unknown	Unknown	urine	Clinical	SRR12526242	VF125AL	WT
B18812	United States	III	2020	2020	Unknown	Unknown	Unknown	aspirate	Clinical	SRR12784121	VF125AL	WT
B18830	United States	III	2020	2020	Unknown	Unknown	Unknown	urine	Clinical	SRR12526239	VF125AL	WT
B18832	United States	III	2020	2020	Unknown	Unknown	Unknown	bronchial washings	Clinical	SRR12526251	VF125AL	WT
B18833	United States	III	2020	2020	Unknown	Unknown	Unknown	wound	Clinical	SRR12526241	VF125AL	WT
B18891	United States	III	2020	2020	Unknown	Unknown	Unknown	blood	Clinical	SRR12526237	VF125AL	WT
B18906	United States	III	2019	2019	Unknown	Unknown	Unknown	blood	Clinical	SRR12526236	VF125AL	WT
B18908	United States	III	2019	2019	Unknown	Unknown	Unknown	blood	Clinical	SRR12526238	VF125AL	WT
B18919	United States	III	2020	2020	Unknown	Unknown	Unknown	sputum	Clinical	SRR12526240	VF125AL	WT
B18925	United States	III	2020	2020	Unknown	Unknown	Unknown	blood	Clinical	SRR12526234	VF125AL	WT
B18926	United States	III	2020	2020	Unknown	Unknown	Unknown	blood	Clinical	SRR12526252	VF125AL	WT
B18933	United States	III	2020	2020	Unknown	Unknown	Unknown	blood	Clinical	SRR12784123	VF125AL	WT
B18976	United States	III	2020	2020	Unknown	Unknown	Unknown	urine	Clinical	SRR12526249	VF125AL	WT
B18977	United States	III	2020	2020	Unknown	Unknown	Unknown	blood	Clinical	SRR12526248	VF125AL	WT
B18981	United States	III	2020	2020	Unknown	Unknown	Unknown	bronchial washings	Clinical	SRR12526246	VF125AL	WT
B18982	United States	III	2020	2020	Unknown	Unknown	Unknown	blood	Clinical	SRR12526245	VF125AL	WT
B18995	United States	III	2020	2020	Unknown	Unknown	Unknown	wound	Clinical	SRR12526244	VF125AL	WT
B18996	United States	III	2020	2020	Unknown	Unknown	Unknown	blood	Clinical	SRR12526243	VF125AL	WT
B18997	United States	III	2020	2020	Unknown	Unknown	Unknown	urine	Clinical	SRR12784128	VF125AL	WT
B19006	United States	III	2020	2020	Unknown	Unknown	Unknown	blood	Clinical	SRR12784127	WT	WT
B19007	United States	III	2020	2020	Unknown	Unknown	Unknown	urine	Clinical	SRR12784126	VF125AL	WT
B19024	United States	III	2020	2020	Unknown	Unknown	Unknown	blood	Clinical	SRR12784120	VF125AL	WT
B19025	United States	III	2020	2020	Unknown	Unknown	Unknown	urine	Clinical	SRR12784119	VF125AL	WT
B19026	United States	III	2020	2020	Unknown	Unknown	Unknown	sputum	Clinical	SRR12784140	VF125AL	WT
B19029	United States	III	2020	2020	Unknown	Unknown	Unknown	urine	Clinical	SRR12784139	VF125AL	WT
B19030	United States	III	2020	2020	Unknown	Unknown	Unknown	urine	Clinical	SRR12784138	VF125AL	WT
B19055	United States	III	2020	2020	Unknown	Unknown	Unknown	blood	Clinical	SRR12784137	VF125AL	WT
B19056	United States	III	2020	2020	Unknown	Unknown	Unknown	urine	Clinical	SRR12784136	VF125AL	WT
B19062	United States	III	2020	2020	Unknown	Unknown	Unknown	wound	Clinical	SRR12784135	VF125AL	WT
B19065	United States	III	2020	2020	Unknown	Unknown	Unknown	blood	Clinical	SRR12784134	VF125AL	WT
B19066	United States	III	2020	2020	Unknown	Unknown	Unknown	aspirate	Clinical	SRR12784133	VF125AL	WT
B19069	United States	III	2020	2020	Unknown	Unknown	Unknown	sputum	Clinical	SRR12784122	VF125AL	WT
B19131	United States	III	2020	2020	Unknown	Unknown	Unknown	blood	Clinical	SRR12784131	VF125AL	WT
B19132	United States	III	2020	2020	Unknown	Unknown	Unknown	trachea aspirate	Clinical	SRR12784129	VF125AL	WT
B19285	United States	III	2020-09-03	2020	Unknown	Unknown	Unknown	urine	Clinical	SRR14590379	VF125AL	WT
B19448	United States	III	2020-10-02	2020	Unknown	Unknown	Unknown	leg	Clinical	SRR14590390	VF125AL	WT
B19584	United States	III	2020-10-29	2020	Unknown	Unknown	Unknown	Unknown	Unknown	SRR14590383	VF125AL	WT
B19913	United States	III	2020-11-16	2020	Unknown	Unknown	Unknown	blood	Clinical	SRR14590389	VF125AL	WT
B19920	United States	III	2021-01-04	2021	Unknown	Unknown	Unknown	urine	Clinical	SRR14590388	VF125AL	WT
B19944	United States	III	2021-01-24	2021	Unknown	Unknown	Unknown	sputum	Clinical	SRR14590384	VF125AL	WT
B19983	United States	III	2021-01-19	2021	Unknown	Unknown	Unknown	wound	Clinical	SRR14590386	VF125AL	WT
B19985	United States	III	2021-01-15	2021	Unknown	Unknown	Unknown	wound	Clinical	SRR14590385	VF125AL	WT
C12-A109	China	III	2018-03-09	2018	Resistant	Susceptible	Susceptible	Environmental	Environmental	SRR9316725	VF125AL	WT
C20955	United States	III	2019-04-18	2019	Unknown	Unknown	Unknown	axilla and groin	Clinical	SRR17577053	VF125AL	WT
C21072	United States	III	2019-04-22	2019	Unknown	Unknown	Unknown	axilla and groin	Clinical	SRR17577052	VF125AL	WT
C21086	United States	III	2019-04-22	2019	Unknown	Unknown	Unknown	axilla and groin	Clinical	SRR17577051	VF125AL	WT
C21485	United States	III	2019-04-25	2019	Unknown	Unknown	Unknown	axilla and groin	Clinical	SRR17577050	VF125AL	WT
C45587	United States	III	2019-05-07	2019	Unknown	Unknown	Unknown	axilla and groin	Clinical	SRR17577048	VF125AL	WT
C45616	United States	III	2019-05-07	2019	Unknown	Unknown	Unknown	axilla and groin	Clinical	SRR17577047	VF125AL	WT
C45954	United States	III	2019-05-08	2019	Unknown	Unknown	Unknown	axilla and groin	Clinical	SRR17577046	VF125AL	WT
C45960	United States	III	2019-05-08	2019	Unknown	Unknown	Unknown	axilla and groin	Clinical	SRR17577045	VF125AL	WT
C45964	United States	III	2019-05-08	2019	Unknown	Unknown	Unknown	axilla and groin	Clinical	SRR17577044	VF125AL	WT
C45965	United States	III	2019-05-08	2019	Unknown	Unknown	Unknown	axilla and groin	Clinical	SRR17577043	VF125AL	WT
C45969	United States	III	2019-05-08	2019	Unknown	Unknown	Unknown	axilla and groin	Clinical	SRR17577042	VF125AL	WT
C45991	United States	III	2019-05-08	2019	Unknown	Unknown	Unknown	axilla and groin	Clinical	SRR17577041	VF125AL	WT
C46002	United States	III	2019-05-08	2019	Unknown	Unknown	Unknown	axilla and groin	Clinical	SRR17577040	VF125AL	WT
C46008	United States	III	2019-05-08	2019	Unknown	Unknown	Unknown	axilla and groin	Clinical	SRR17577039	VF125AL	WT
C46010	United States	III	2019-05-08	2019	Unknown	Unknown	Unknown	axilla and groin	Clinical	SRR17577081	VF125AL	WT
C46013	United States	III	2019-05-08	2019	Unknown	Unknown	Unknown	axilla and groin	Clinical	SRR17577080	VF125AL	WT
C46014	United States	III	2019-05-08	2019	Unknown	Unknown	Unknown	axilla and groin	Clinical	SRR17577079	VF125AL	WT
C46015	United States	III	2019-05-08	2019	Unknown	Unknown	Unknown	axilla and groin	Clinical	SRR17577078	VF125AL	WT
C46020	United States	III	2019-05-08	2019	Unknown	Unknown	Unknown	axilla and groin	Clinical	SRR17577077	VF125AL	WT
C46022	United States	III	2019-05-08	2019	Unknown	Unknown	Unknown	axilla and groin	Clinical	SRR17577076	VF125AL	WT
C46027	United States	III	2019-05-08	2019	Unknown	Unknown	Unknown	axilla and groin	Clinical	SRR17577075	VF125AL	WT
C46062	United States	III	2019-05-09	2019	Unknown	Unknown	Unknown	axilla and groin	Clinical	SRR17577074	VF125AL	WT
C46107	United States	III	2019-05-09	2019	Unknown	Unknown	Unknown	axilla and groin	Clinical	SRR17577073	VF125AL	WT
C46121	United States	III	2019-05-09	2019	Unknown	Unknown	Unknown	axilla and groin	Clinical	SRR17577072	VF125AL	WT
CA S97	Australia	III	2015-07-15	2015	Resistant	Susceptible	Susceptible	Bone	Clinical	SRR7657927	VF125AL	WT
CA-OCPHL-CAU-00001	United States	III	2019-07-29	2019	Unknown	Unknown	Unknown	sputum	Clinical	SRR24806737	VF125AL	WT
CA-OCPHL-CAU-00002	United States	III	2021-04-27	2021	Unknown	Unknown	Unknown	blood	Clinical	SRR24806704	VF125AL	WT
CA-OCPHL-CAU-00003	United States	III	2021-05-07	2021	Unknown	Unknown	Unknown	urine	Clinical	SRR24806760	VF125AL	S639F
CA-OCPHL-CAU-00004	United States	III	2021-05-12	2021	Unknown	Unknown	Unknown	urine	Clinical	SRR24806759	VF125AL	WT
CA-OCPHL-CAU-00005	United States	III	2021-05-28	2021	Unknown	Unknown	Unknown	body fluid	Clinical	SRR24806693	VF125AL	WT
CA-OCPHL-CAU-00007	United States	III	2021-07-30	2021	Unknown	Unknown	Unknown	urine	Clinical	SRR24806763	VF125AL	WT

CA-OCPHL-CAU-00008	United States	III	2021-09-03	2021	Unknown	Unknown	Unknown	wound	Clinical	SRR24806764	VF125AL	WT
CA-OCPHL-CAU-00009	United States	III	2021-09-28	2021	Unknown	Unknown	Unknown	urine	Clinical	SRR24806761	VF125AL	WT
CA-OCPHL-CAU-00010	United States	III	2021-10-20	2021	Unknown	Unknown	Unknown	blood	Clinical	SRR24806767	VF125AL	WT
CA-OCPHL-CAU-00011	United States	III	2021-11-11	2021	Unknown	Unknown	Unknown	Tissue	Clinical	SRR24806715	VF125AL	WT
CA-OCPHL-CAU-00012	United States	III	2021-11-20	2021	Unknown	Unknown	Unknown	urine	Clinical	SRR24806766	VF125AL	WT
CA-OCPHL-CAU-00013	United States	III	2021-12-16	2021	Unknown	Unknown	Unknown	urine	Clinical	SRR24806748	VF125AL	WT
CA-OCPHL-CAU-00014	United States	III	2022-01-02	2022	Unknown	Unknown	Unknown	blood	Clinical	SRR24806783	VF125AL	WT
CA-OCPHL-CAU-00015	United States	III	2022-01-09	2022	Unknown	Unknown	Unknown	blood	Clinical	SRR24806682	VF125AL	WT
CA-OCPHL-CAU-00017	United States	III	2022-07-01	2022	Unknown	Unknown	Unknown	urine	Clinical	SRR24806765	VF125AL	WT
CA-OCPHL-CAU-00018	United States	III	2022-08-06	2022	Unknown	Unknown	Unknown	blood	Clinical	SRR24806784	VF125AL	WT
CA-OCPHL-CAU-00019	United States	III	2022-10-13	2022	Unknown	Unknown	Unknown	Tissue	Clinical	SRR24806726	VF125AL	WT
CA-OCPHL-CAU-00020	United States	III	2022-11-10	2022	Unknown	Unknown	Unknown	wound	Clinical	SRR24806762	VF125AL	WT
CA-OCPHL-CAU-00021	United States	III	2022-01-03	2022	Unknown	Unknown	Unknown	wound	Clinical	SRR24806755	VF125AL	WT
CA-OCPHL-CAU-00022	United States	III	2022-05-10	2022	Unknown	Unknown	Unknown	BAL	Clinical	SRR24806757	VF125AL	WT
CA-OCPHL-CAU-00023	United States	III	2022-06-16	2022	Unknown	Unknown	Unknown	sputum	Clinical	SRR24806758	VF125AL	WT
CA-OCPHL-CAU-00024	United States	III	2022-07-09	2022	Unknown	Unknown	Unknown	body fluid	Clinical	SRR24806754	VF125AL	WT
CA-OCPHL-CAU-00025	United States	III	2022-08-25	2022	Unknown	Unknown	Unknown	urine	Clinical	SRR24806752	VF125AL	WT
CA-OCPHL-CAU-00027	United States	III	2022-11-06	2022	Unknown	Unknown	Unknown	Tissue	Clinical	SRR24806753	VF125AL	WT
CA-OCPHL-CAU-00028	United States	III	2021-07-14	2021	Unknown	Unknown	Unknown	Axilla/Groin	Clinical	SRR24806751	VF125AL	WT
CA-OCPHL-CAU-00030	United States	III	2022-06-07	2022	Unknown	Unknown	Unknown	wound	Clinical	SRR24806750	VF125AL	WT
CA-OCPHL-CAU-00031	United States	III	2022-10-07	2022	Unknown	Unknown	Unknown	blood	Clinical	SRR24806749	VF125AL	WT
CA-OCPHL-CAU-00032	United States	III	2022-06-03	2022	Unknown	Unknown	Unknown	wound	Clinical	SRR24806747	VF125AL	WT
CA-OCPHL-CAU-00033	United States	III	2022-12-18	2022	Unknown	Unknown	Unknown	Axilla/Groin	Clinical	SRR24806741	VF125AL	WT
CA-OCPHL-CAU-00034	United States	III	2022-12-18	2022	Unknown	Unknown	Unknown	Axilla/Groin	Clinical	SRR24806731	VF125AL	WT
CA-OCPHL-CAU-00035	United States	III	2022-12-04	2022	Unknown	Unknown	Unknown	Axilla/Groin	Clinical	SRR24806738	VF125AL	WT
CA-OCPHL-CAU-00036	United States	III	2022-11-02	2022	Unknown	Unknown	Unknown	Axilla/Groin	Clinical	SRR24806743	VF125AL	WT
CA-OCPHL-CAU-00037	United States	III	2022-11-01	2022	Unknown	Unknown	Unknown	Axilla/Groin	Clinical	SRR24806740	VF125AL	WT
CA-OCPHL-CAU-00038	United States	III	2022-10-19	2022	Unknown	Unknown	Unknown	Axilla/Groin	Clinical	SRR24806742	VF125AL	WT
CA-OCPHL-CAU-00039	United States	III	2022-10-19	2022	Unknown	Unknown	Unknown	Axilla/Groin	Clinical	SRR24806736	VF125AL	WT
CA-OCPHL-CAU-00040	United States	III	2022-09-13	2022	Unknown	Unknown	Unknown	Axilla/Groin	Clinical	SRR24806735	VF125AL	WT
CA-OCPHL-CAU-00041	United States	III	2022-02-15	2022	Unknown	Unknown	Unknown	Axilla/Groin	Clinical	SRR24806744	VF125AL	WT
CA-OCPHL-CAU-00042	United States	III	2022-10-26	2022	Unknown	Unknown	Unknown	Axilla/Groin	Clinical	SRR24806728	VF125AL	WT
CA-OCPHL-CAU-00043	United States	III	2023-01-04	2023	Unknown	Unknown	Unknown	Axilla/Groin	Clinical	SRR24806745	VF125AL	WT
CA-OCPHL-CAU-00044	United States	III	2023-01-04	2023	Unknown	Unknown	Unknown	Axilla/Groin	Clinical	SRR24806734	VF125AL	WT
CA-OCPHL-CAU-00045	United States	III	2022-09-06	2022	Unknown	Unknown	Unknown	Axilla/Groin	Clinical	SRR24806727	VF125AL	WT
CA-OCPHL-CAU-00046	United States	III	2022-06-20	2022	Unknown	Unknown	Unknown	Axilla/Groin	Clinical	SRR24806732	VF125AL	WT
CA-OCPHL-CAU-00047	United States	III	2022-05-19	2022	Unknown	Unknown	Unknown	Axilla/Groin	Clinical	SRR24806730	VF125AL	WT
CA-OCPHL-CAU-00048	United States	III	2022-03-28	2022	Unknown	Unknown	Unknown	Axilla/Groin	Clinical	SRR24806729	VF125AL	WT
CA-OCPHL-CAU-00049	United States	III	2022-03-28	2022	Unknown	Unknown	Unknown	Axilla/Groin	Clinical	SRR24806739	VF125AL	WT
CA-OCPHL-CAU-00050	United States	III	2022-03-28	2022	Unknown	Unknown	Unknown	Axilla/Groin	Clinical	SRR24806746	VF125AL	WT
CA-OCPHL-CAU-00051	United States	III	2023-01-17	2023	Unknown	Unknown	Unknown	Axilla/Groin	Clinical	SRR24806733	VF125AL	WT
CA-OCPHL-CAU-00052	United States	III	2022-04-08	2022	Unknown	Unknown	Unknown	wound	Clinical	SRR24806725	VF125AL	WT
CA-OCPHL-CAU-00053	United States	III	2022-09-28	2022	Unknown	Unknown	Unknown	Peg site	Clinical	SRR24806724	VF125AL	WT
CA-OCPHL-CAU-00054	United States	III	2023-01-04	2023	Unknown	Unknown	Unknown	Tissue	Clinical	SRR24806723	VF125AL	WT
CA-OCPHL-CAU-00055	United States	III	2021-12-18	2021	Unknown	Unknown	Unknown	wound	Clinical	SRR24806722	VF125AL	WT
CA-OCPHL-CAU-00056	United States	III	2022-02-27	2022	Unknown	Unknown	Unknown	urine	Clinical	SRR24806721	VF125AL	WT
CA-OCPHL-CAU-00057	United States	III	2022-05-28	2022	Unknown	Unknown	Unknown	urine	Clinical	SRR24806720	VF125AL	WT
CA-OCPHL-CAU-00059	United States	III	2022-07-15	2022	Unknown	Unknown	Unknown	urine	Clinical	SRR24806718	VF125AL	WT
CA-OCPHL-CAU-00060	United States	III	2022-10-19	2022	Unknown	Unknown	Unknown	wound	Clinical	SRR24806717	VF125AL	WT
CA-OCPHL-CAU-00061	United States	III	2022-11-13	2022	Unknown	Unknown	Unknown	Axilla/Groin	Clinical	SRR24806716	VF125AL	WT
CA-OCPHL-CAU-00062	United States	III	2022-11-14	2022	Unknown	Unknown	Unknown	Axilla/Groin	Clinical	SRR24806714	VF125AL	WT
CA-OCPHL-CAU-00063	United States	III	2023-01-01	2023	Unknown	Unknown	Unknown	Tissue	Clinical	SRR24806713	VF125AL	WT
CA-OCPHL-CAU-00064	United States	III	2022-10-23	2022	Unknown	Unknown	Unknown	urine	Clinical	SRR24806712	VF125AL	WT
CA-OCPHL-CAU-00066	United States	III	2022-09-20	2022	Unknown	Unknown	Unknown	urine	Clinical	SRR24806710	VF125AL	WT
CA-OCPHL-CAU-00067	United States	III	2021-06-26	2021	Unknown	Unknown	Unknown	tracheal aspirate	Clinical	SRR24806708	VF125AL	WT
CA-OCPHL-CAU-00068	United States	III	2021-07-19	2021	Unknown	Unknown	Unknown	Resp Processed	Clinical	SRR24806707	VF125AL	WT
CA-OCPHL-CAU-00069	United States	III	2021-07-31	2021	Unknown	Unknown	Unknown	wound	Clinical	SRR24806706	VF125AL	WT
CA-OCPHL-CAU-00070	United States	III	2021-09-11	2021	Unknown	Unknown	Unknown	tracheal aspirate	Clinical	SRR24806705	VF125AL	WT
CA-OCPHL-CAU-00071	United States	III	2021-10-11	2021	Unknown	Unknown	Unknown	urine	Clinical	SRR24806703	VF125AL	WT
CA-OCPHL-CAU-00072	United States	III	2021-10-21	2021	Unknown	Unknown	Unknown	urine	Clinical	SRR24806702	VF125AL	WT
CA-OCPHL-CAU-00073	United States	III	2021-10-29	2021	Unknown	Unknown	Unknown	urine	Clinical	SRR24806701	VF125AL	WT
CA-OCPHL-CAU-00074	United States	III	2021-12-03	2021	Unknown	Unknown	Unknown	blood	Clinical	SRR24806700	VF125AL	WT
CA-OCPHL-CAU-00075	United States	III	2021-12-17	2021	Unknown	Unknown	Unknown	blood	Clinical	SRR24806699	VF125AL	WT
CA-OCPHL-CAU-00076	United States	III	2021-12-29	2021	Unknown	Unknown	Unknown	Catheter	Clinical	SRR24806698	VF125AL	WT
CA-OCPHL-CAU-00077	United States	III	2022-02-24	2022	Unknown	Unknown	Unknown	urine	Clinical	SRR24806697	VF125AL	WT
CA-OCPHL-CAU-00078	United States	III	2022-04-15	2022	Unknown	Unknown	Unknown	blood	Clinical	SRR24806696	VF125AL	WT
CA-OCPHL-CAU-00079	United States	III	2022-04-27	2022	Unknown	Unknown	Unknown	blood	Clinical	SRR24806695	VF125AL	WT
CA-OCPHL-CAU-00080	United States	III	2022-07-11	2022	Unknown	Unknown	Unknown	Respiratory Process	Clinical	SRR24806709	VF125AL	WT
CA-OCPHL-CAU-00081	United States	III	2022-09-30	2022	Unknown	Unknown	Unknown	blood	Clinical	SRR24806694	VF125AL	WT
CA-OCPHL-CAU-00082	United States	III	2021-08-28	2021	Unknown	Unknown	Unknown	Axilla/Groin	Clinical	SRR24806692	VF125AL	WT
CA-OCPHL-CAU-00083	United States	III	2021-09-17	2021	Unknown	Unknown	Unknown	Axilla/Groin	Clinical	SRR24806691	VF125AL	WT
CA-OCPHL-CAU-00084	United States	III	2021-10-01	2021	Unknown	Unknown	Unknown	wound	Clinical	SRR24806690	VF125AL	WT
CA-OCPHL-CAU-00085	United States	III	2021-11-17	2021	Unknown	Unknown	Unknown	urine	Clinical	SRR24806689	VF125AL	WT
CA-OCPHL-CAU-00086	United States	III	2021-11-22	2021	Unknown	Unknown	Unknown	Axilla/Groin	Clinical	SRR24806688	VF125AL	WT
CA-OCPHL-CAU-00087	United States	III	2022-02-08	2022	Unknown	Unknown	Unknown	urine	Clinical	SRR24806687	VF125AL	WT
CA-OCPHL-CAU-00088	United States	III	2022-02-12	2022	Unknown	Unknown	Unknown	blood	Clinical	SRR24806686	VF125AL	WT
CA-OCPHL-CAU-00089	United States	III	2022-03-01	2022	Unknown	Unknown	Unknown	urine	Clinical	SRR24806685	VF125AL	WT
CA-OCPHL-CAU-00090	United States	III	2022-05-13	2022	Unknown	Unknown	Unknown	wound	Clinical	SRR24806684	VF125AL	WT
CA-OCPHL-CAU-00091	United States	III	2022-05-26	2022	Unknown	Unknown	Unknown	Axilla/Groin	Clinical	SRR24806683	VF125AL	WT

CA-OCPHL-CAU-00092	United States	III	2022-05-30	2022	Unknown	Unknown	Unknown	Tissue	Clinical	SRR24806681	VF125AL	WT
CA-OCPHL-CAU-00093	United States	III	2022-06-01	2022	Unknown	Unknown	Unknown	Bronch Wash	Clinical	SRR24806680	VF125AL	WT
CA-OCPHL-CAU-00094	United States	III	2022-07-01	2022	Unknown	Unknown	Unknown	Axilla/Groin	Clinical	SRR24806679	VF125AL	WT
CA-OCPHL-CAU-00095	United States	III	2022-07-08	2022	Unknown	Unknown	Unknown	urine	Clinical	SRR24806678	VF125AL	WT
CA-OCPHL-CAU-00096	United States	III	2022-08-27	2022	Unknown	Unknown	Unknown	Axilla/Groin	Clinical	SRR24806677	VF125AL	WT
CA-OCPHL-CAU-00097	United States	III	2022-09-07	2022	Unknown	Unknown	Unknown	sputum	Clinical	SRR24806676	VF125AL	WT
CA-OCPHL-CAU-00098	United States	III	2022-12-30	2022	Unknown	Unknown	Unknown	urine	Clinical	SRR24806675	VF125AL	WT
CA-OCPHL-CAU-00099	United States	III	2022-12-10	2022	Unknown	Unknown	Unknown	blood	Clinical	SRR24806674	VF125AL	WT
CA-OCPHL-CAU-00100	United States	III	2021-09-14	2021	Unknown	Unknown	Unknown	Axilla/Groin	Clinical	SRR24806673	VF125AL	WT
CA-OCPHL-CAU-00101	United States	III	2023-01-30	2023	Unknown	Unknown	Unknown	urine	Clinical	SRR24806672	VF125AL	WT
CA-OCPHL-CAU-00103	United States	III	2022-11-21	2022	Unknown	Unknown	Unknown	urine	Clinical	SRR24806781	VF125AL	WT
CA-OCPHL-CAU-00104	United States	III	2023-02-17	2023	Unknown	Unknown	Unknown	urine	Clinical	SRR24806780	VF125AL	WT
CA-OCPHL-CAU-00105	United States	III	2023-02-10	2023	Unknown	Unknown	Unknown	wound	Clinical	SRR24806779	VF125AL	WT
CA-OCPHL-CAU-00106	United States	III	2023-01-22	2023	Unknown	Unknown	Unknown	urine	Clinical	SRR24806778	VF125AL	WT
CA-OCPHL-CAU-00108	United States	III	2022-11-28	2022	Unknown	Unknown	Unknown	sputum	Clinical	SRR24806773	VF125AL	WT
CA-OCPHL-CAU-00109	United States	III	2022-12-12	2022	Unknown	Unknown	Unknown	blood	Clinical	SRR24806776	VF125AL	WT
CA-OCPHL-CAU-00111	United States	III	2023-03-16	2023	Unknown	Unknown	Unknown	wound	Clinical	SRR24806775	VF125AL	WT
CA-OCPHL-CAU-00113	United States	III	2022-11-02	2022	Unknown	Unknown	Unknown	urine	Clinical	SRR24806770	VF125AL	WT
CA-OCPHL-CAU-00114	United States	III	2020-05-24	2020	Unknown	Unknown	Unknown	urine	Clinical	SRR24806768	VF125AL	WT
CA-OCPHL-CAU-00115	United States	III	2023-02-21	2023	Unknown	Unknown	Unknown	urine	Clinical	SRR24806769	VF125AL	WT
Cau4	Austria	III	2021-10	2021	Resistant	Susceptible	Susceptible	Throat	Clinical	SRR23080876	VF125AL	WT
CT-WCPHL-CAU-224150	United States	III	2020-09-08	2020	Unknown	Unknown	Unknown	Unknown	Unknown	SRR24621173	VF125AL	WT
DN474116W-A1	United Kingdom	III		2016-2018	Unknown	Unknown	Unknown	clinical	Clinical	ERR2300769	VF125AL	WT
DN474116W-A6	United Kingdom	III		2016-2018	Unknown	Unknown	Unknown	clinical	Clinical	ERR2300809	VF125AL	WT
DN474116W-B1	United Kingdom	III		2016-2018	Unknown	Unknown	Unknown	clinical	Clinical	ERR2300770	VF125AL	WT
DN474116W-B6	United Kingdom	III		2016-2018	Unknown	Unknown	Unknown	clinical	Clinical	ERR2300810	VF125AL	WT
DN474116W-C1	United Kingdom	III		2016-2018	Unknown	Unknown	Unknown	clinical	Clinical	ERR2300771	VF125AL	WT
DN474116W-D1	United Kingdom	III		2016-2018	Unknown	Unknown	Unknown	clinical	Clinical	ERR2300772	VF125AL	WT
DN474116W-D4	United Kingdom	III		2016-2018	Unknown	Unknown	Unknown	clinical	Clinical	ERR2300796	VF125AL	WT
DN474116W-D5	United Kingdom	III		2016-2018	Unknown	Unknown	Unknown	clinical	Clinical	ERR2300804	VF125AL	WT
DN474116W-E3	United Kingdom	III		2016-2018	Unknown	Unknown	Unknown	clinical	Clinical	ERR2300789	VF125AL	WT
DN474116W-E4	United Kingdom	III		2016-2018	Unknown	Unknown	Unknown	clinical	Clinical	ERR2300797	VF125AL	WT
DN474116W-F4	United Kingdom	III		2016-2018	Unknown	Unknown	Unknown	clinical	Clinical	ERR2300798	VF125AL	WT
DN474116W-G2	United Kingdom	III		2016-2018	Unknown	Unknown	Unknown	clinical	Clinical	ERR2300783	VF125AL	WT
GCF 002775015.1	South Africa	III	2012-10-23	2012	Resistant	Susceptible	Susceptible	Blood	Clinical	Reference assembly	VF125AL	WT
Hoist	United Kingdom	III	2017-04-04	2017	Unknown	Unknown	Unknown	Environmental	Environmental	SRR7976583	VF125AL	WT
MRL 2491	South Africa	III	2017	2017	Unknown	Unknown	Unknown	Blood	Clinical	SRR14802846	VF125AL	WT
MRL 2526	South Africa	III	2017	2017	Unknown	Unknown	Unknown	Blood	Clinical	SRR14802845	VF125AL	WT
MRL 2546	South Africa	III	2017	2017	Unknown	Unknown	Unknown	Blood	Clinical	SRR14802843	VF125AL	WT
MRL 2547	South Africa	III	2017	2017	Unknown	Unknown	Unknown	Blood	Clinical	SRR14802842	VF125AL	WT
MRL 2566	South Africa	III	2017	2017	Unknown	Unknown	Unknown	Blood	Clinical	SRR14802841	VF125AL	WT
MRL 2609	South Africa	III	2017	2017	Unknown	Unknown	Unknown	Blood	Clinical	SRR14802840	VF125AL	WT
MRL 2634	South Africa	III	2017	2017	Unknown	Unknown	Unknown	Blood	Clinical	SRR14802839	VF125AL	WT
MRL 2678	South Africa	III	2017	2017	Unknown	Unknown	Unknown	Blood	Clinical	SRR14802838	VF125AL	WT
MRL 2736	South Africa	III	2017	2017	Unknown	Unknown	Unknown	Blood	Clinical	SRR14802836	VF125AL	WT
MRL 2859	South Africa	III	2017	2017	Unknown	Unknown	Unknown	Blood	Clinical	SRR14802835	VF125AL	WT
MRL 2980	South Africa	III	2017	2017	Unknown	Unknown	Unknown	Blood	Clinical	SRR14802834	VF125AL	WT
MRL 3225	South Africa	III	2017	2017	Unknown	Unknown	Unknown	Blood	Clinical	SRR14802832	VF125AL	WT
MRL 3269	South Africa	III	2017	2017	Unknown	Unknown	Unknown	Blood	Clinical	SRR14802940	VF125AL	WT
MRL 3289	South Africa	III	2017	2017	Unknown	Unknown	Unknown	Blood	Clinical	SRR14802938	VF125AL	WT
MRL 3338	South Africa	III	2017	2017	Unknown	Unknown	Unknown	Blood	Clinical	SRR14802937	VF125AL	WT
MRL 3345	South Africa	III	2017	2017	Unknown	Unknown	Unknown	Blood	Clinical	SRR14802936	VF125AL	WT
MRL 3402	South Africa	III	2017	2017	Unknown	Unknown	Unknown	Blood	Clinical	SRR14802935	VF125AL	WT
MRL 3405	South Africa	III	2017	2017	Unknown	Unknown	Unknown	Blood	Clinical	SRR14802934	VF125AL	WT
MRL 3406	South Africa	III	2017	2017	Unknown	Unknown	Unknown	Blood	Clinical	SRR14802933	VF125AL	WT
MRL 3411	South Africa	III	2017	2017	Unknown	Unknown	Unknown	Blood	Clinical	SRR14802932	VF125AL	WT
MRL 3511	South Africa	III	2017	2017	Unknown	Unknown	Unknown	Blood	Clinical	SRR14802931	VF125AL	WT
MRL 3560	South Africa	III	2017	2017	Unknown	Unknown	Unknown	Blood	Clinical	SRR14802928	VF125AL	S639P
MRL 3561	South Africa	III	2017	2017	Unknown	Unknown	Unknown	Blood	Clinical	SRR14802927	VF125AL	S639P
MRL 3562	South Africa	III	2017	2017	Unknown	Unknown	Unknown	Blood	Clinical	SRR14802926	VF125AL	S639P
MRL 3589	South Africa	III	2017	2017	Unknown	Unknown	Unknown	Blood	Clinical	SRR14802925	VF125AL	WT
MRL 3706	South Africa	III	2017	2017	Unknown	Unknown	Unknown	Blood	Clinical	SRR14802924	VF125AL	WT
MRL 3758	South Africa	III	2017	2017	Unknown	Unknown	Unknown	Blood	Clinical	SRR14802923	WT	WT
MRL 3770	South Africa	III	2017	2017	Unknown	Unknown	Unknown	Blood	Clinical	SRR14802922	VF125AL	WT
MRL 3788	South Africa	III	2017	2017	Unknown	Unknown	Unknown	Blood	Clinical	SRR14802921	VF125AL	WT
MRL 4152	South Africa	III	2017	2017	Unknown	Unknown	Unknown	Blood	Clinical	SRR14802920	VF125AL	WT
MRL 4414	South Africa	III	2017	2017	Unknown	Unknown	Unknown	Blood	Clinical	SRR14802918	VF125AL	WT
MRL 4603	South Africa	III	2017	2017	Unknown	Unknown	Unknown	Blood	Clinical	SRR14802916	VF125AL	WT
MRL 4642	South Africa	III	2017	2017	Unknown	Unknown	Unknown	Blood	Clinical	SRR14802915	VF125AL	WT
MRL 4742	South Africa	III	2017	2017	Unknown	Unknown	Unknown	Blood	Clinical	SRR14802914	VF125AL	WT
MRL 4836	South Africa	III	2017	2017	Unknown	Unknown	Unknown	Blood	Clinical	SRR14802913	VF125AL	WT
MRL 4845	South Africa	III	2017	2017	Unknown	Unknown	Unknown	Blood	Clinical	SRR14802912	VF125AL	WT
MRL 4888	South Africa	III	2017	2017	Unknown	Unknown	Unknown	Blood	Clinical	SRR14802911	VF125AL	WT
MRL 4895	South Africa	III	2017	2017	Unknown	Unknown	Unknown	Blood	Clinical	SRR14802910	VF125AL	WT
MRL 4925	South Africa	III	2017	2017	Unknown	Unknown	Unknown	Blood	Clinical	SRR14802907	VF125AL	WT
MRL 4926	South Africa	III	2017	2017	Unknown	Unknown	Unknown	Blood	Clinical	SRR14802906	VF125AL	WT
MRL 4928	South Africa	III	2017	2017	Unknown	Unknown	Unknown	Blood	Clinical	SRR14802905	VF125AL	WT
MRL 4930	South Africa	III	2017	2017	Unknown	Unknown	Unknown	Blood	Clinical	SRR14802904	VF125AL	WT
MRL 4943	South Africa	III	2017	2017	Unknown	Unknown	Unknown	Blood	Clinical	SRR14802902	VF125AL	WT

MRL 4944	South Africa	III	2017	2017	Unknown	Unknown	Unknown	Blood	Clinical	SRR14802901	VF125AL	WT
MRL 5006	South Africa	III	2017	2017	Unknown	Unknown	Unknown	Blood	Clinical	SRR14802900	VF125AL	WT
MRL 5109	South Africa	III	2017	2017	Unknown	Unknown	Unknown	Blood	Clinical	SRR14802899	VF125AL	WT
MRL 5222	South Africa	III	2017	2017	Unknown	Unknown	Unknown	Blood	Clinical	SRR14802895	VF125AL	WT
MRL 5268	South Africa	III	2017	2017	Unknown	Unknown	Unknown	Blood	Clinical	SRR14802893	VF125AL	WT
MRL 5305	South Africa	III	2017	2017	Unknown	Unknown	Unknown	Blood	Clinical	SRR14802892	VF125AL	WT
MRL 5386	South Africa	III	2017	2017	Unknown	Unknown	Unknown	Blood	Clinical	SRR14802891	VF125AL	WT
MRL 5405	South Africa	III	2017	2017	Unknown	Unknown	Unknown	Blood	Clinical	SRR14802890	VF125AL	WT
MRL 5483	South Africa	III	2017	2017	Unknown	Unknown	Unknown	Blood	Clinical	SRR14802889	VF125AL	WT
MRL 5543	South Africa	III	2017	2017	Unknown	Unknown	Unknown	Blood	Clinical	SRR14802888	VF125AL	WT
MRL 5547	South Africa	III	2017	2017	Unknown	Unknown	Unknown	Blood	Clinical	SRR14802887	VF125AL	WT
MRL 5561	South Africa	III	2017	2017	Unknown	Unknown	Unknown	Blood	Clinical	SRR14802885	VF125AL	WT
MRL 5574	South Africa	III	2017	2017	Unknown	Unknown	Unknown	Blood	Clinical	SRR14802884	VF125AL	WT
MRL 5580	South Africa	III	2017	2017	Unknown	Unknown	Unknown	Blood	Clinical	SRR14802883	VF125AL	WT
MRL 5585	South Africa	III	2017	2017	Unknown	Unknown	Unknown	Blood	Clinical	SRR14802882	VF125AL	WT
MRL 5590	South Africa	III	2017	2017	Unknown	Unknown	Unknown	Blood	Clinical	SRR14802897	VF125AL	WT
MRL 5591	South Africa	III	2017	2017	Unknown	Unknown	Unknown	Blood	Clinical	SRR14802886	VF125AL	WT
MRL 5593	South Africa	III	2017	2017	Unknown	Unknown	Unknown	Blood	Clinical	SRR14802881	VF125AL	WT
MRL 5624	South Africa	III	2017	2017	Unknown	Unknown	Unknown	Blood	Clinical	SRR14802880	VF125AL	WT
MRL 5625	South Africa	III	2017	2017	Unknown	Unknown	Unknown	Blood	Clinical	SRR14802879	VF125AL	WT
MRL 5704	South Africa	III	2017	2017	Unknown	Unknown	Unknown	Blood	Clinical	SRR14802878	VF125AL	WT
MRL 5714	South Africa	III	2017	2017	Unknown	Unknown	Unknown	Blood	Clinical	SRR14802875	VF125AL	WT
MRL 5715	South Africa	III	2017	2017	Unknown	Unknown	Unknown	Blood	Clinical	SRR14802944	VF125AL	WT
MRL 5716	South Africa	III	2017	2017	Unknown	Unknown	Unknown	Blood	Clinical	SRR14802858	VF125AL	WT
MRL 5734	South Africa	III	2017	2017	Unknown	Unknown	Unknown	Blood	Clinical	SRR14802877	VF125AL	WT
MRL 5762	South Africa	III	2017	2017	Unknown	Unknown	Unknown	Blood	Clinical	SRR14802852	VF125AL	WT
MRL 5771	South Africa	III	2017	2017	Unknown	Unknown	Unknown	Blood	Clinical	SRR14802876	VF125AL	WT
MRL 5946	South Africa	III	2017	2017	Unknown	Unknown	Unknown	Blood	Clinical	SRR14802874	VF125AL	WT
MRL 5948	South Africa	III	2017	2017	Unknown	Unknown	Unknown	Blood	Clinical	SRR14802850	VF125AL	WT
MRL 5953	South Africa	III	2017	2017	Unknown	Unknown	Unknown	Blood	Clinical	SRR14802873	VF125AL	WT
MRL 6057	South Africa	III	2017	2017	Unknown	Unknown	Unknown	Blood	Clinical	SRR14802849	VF125AL	WT
MRL 6241	South Africa	III	2017	2017	Unknown	Unknown	Unknown	Blood	Clinical	SRR14802872	VF125AL	WT
MRL 6277b	South Africa	III	2017	2017	Unknown	Unknown	Unknown	Blood	Clinical	SRR14802871	VF125AL	WT
MRL B159-2	South Africa	III	2017	2017	Unknown	Unknown	Unknown	Environmental	Environmental	SRR14802870	VF125AL	WT
MRL B195-1	South Africa	III	2017	2017	Unknown	Unknown	Unknown	Environmental	Environmental	SRR14802869	VF125AL	WT
MRL B235-1	South Africa	III	2017	2017	Unknown	Unknown	Unknown	Environmental	Environmental	SRR14802867	VF125AL	WT
MRL B296-3	South Africa	III	2017	2017	Unknown	Unknown	Unknown	Environmental	Environmental	SRR14802942	VF125AL	WT
MRL S158-1	South Africa	III	2017	2017	Unknown	Unknown	Unknown	Environmental	Environmental	SRR14802939	VF125AL	WT
MRL S16	South Africa	III	2017	2017	Unknown	Unknown	Unknown	Axilla/Groin swab	Clinical	SRR14802943	VF125AL	WT
MRL S19	South Africa	III	2017	2017	Unknown	Unknown	Unknown	Axilla/Groin swab	Clinical	SRR14802868	VF125AL	WT
MRL S2	South Africa	III	2017	2017	Unknown	Unknown	Unknown	Axilla/Groin swab	Clinical	SRR14802866	VF125AL	WT
MRL S200-1	South Africa	III	2017	2017	Unknown	Unknown	Unknown	Environmental	Environmental	SRR14802865	VF125AL	WT
MRL S26	South Africa	III	2017	2017	Unknown	Unknown	Unknown	Axilla/Groin swab	Clinical	SRR14802864	VF125AL	WT
MRL S27	South Africa	III	2017	2017	Unknown	Unknown	Unknown	Axilla/Groin swab	Clinical	SRR14802863	VF125AL	WT
MRL S29	South Africa	III	2017	2017	Unknown	Unknown	Unknown	Axilla/Groin swab	Clinical	SRR14802862	VF125AL	WT
MRL S31	South Africa	III	2017	2017	Unknown	Unknown	Unknown	Axilla/Groin swab	Clinical	SRR14802861	VF125AL	WT
MRL S316-2	South Africa	III	2017	2017	Unknown	Unknown	Unknown	Environmental	Environmental	SRR14802860	VF125AL	WT
MRL S33	South Africa	III	2017	2017	Unknown	Unknown	Unknown	Axilla/Groin swab	Clinical	SRR14802859	VF125AL	WT
MRL S353-2	South Africa	III	2017	2017	Unknown	Unknown	Unknown	Environmental	Environmental	SRR14802857	VF125AL	WT
MRL S360-1	South Africa	III	2017	2017	Unknown	Unknown	Unknown	Environmental	Environmental	SRR14802856	VF125AL	WT
MRL S4	South Africa	III	2017	2017	Unknown	Unknown	Unknown	Axilla/Groin swab	Clinical	SRR14802855	VF125AL	WT
MRL S6	South Africa	III	2017	2017	Unknown	Unknown	Unknown	Axilla/Groin swab	Clinical	SRR14802854	VF125AL	WT
MRL W-KNH046	South Africa	III	2017	2017	Unknown	Unknown	Unknown	Environmental	Environmental	SRR14802853	VF125AL	WT
N18-02-486	Canada	III	2012	2012	Susceptible	Susceptible	Susceptible	Ear	Clinical	SRR15192316	WT	WT
N19-01-047	Canada	III	2019	2019	Resistant	Susceptible	Susceptible	Bronchial wash	Clinical	SRR15192306	VF125AL	WT
NICU10-A58	China	III	2018-02-03	2018	Resistant	Susceptible	Susceptible	Urine	Clinical	SRR9316776	VF125AL	WT
NICU11-A77	China	III	2018-05-12	2018	Resistant	Susceptible	Susceptible	Blood	Clinical	SRR9316803	VF125AL	WT
NICU12-A79	China	III	2018-05-15	2018	Resistant	Susceptible	Susceptible	Urine	Clinical	SRR9316800	VF125AL	WT
NICU13-A93	China	III	2018-08-10	2018	Resistant	Susceptible	Susceptible	Urine	Clinical	SRR9316812	VF125AL	WT
NICU14-A96	China	III	2018-08-27	2018	Resistant	Susceptible	Susceptible	Urine	Clinical	SRR9316734	VF125AL	WT
NICU15-A100	China	III	2018-09-04	2018	Resistant	Susceptible	Susceptible	Urine	Clinical	SRR9316790	VF125AL	WT
NICU16-A101	China	III	2018-09-04	2018	Resistant	Susceptible	Susceptible	Urine	Clinical	SRR9316791	VF125AL	WT
NICU2-A13	China	III	2016-12-24	2016	Resistant	Susceptible	Susceptible	Urine	Clinical	SRR9316739	VF125AL	WT
NICU3-A15	China	III	2017-02-04	2017	Resistant	Susceptible	Susceptible	Urine	Clinical	SRR9316741	VF125AL	WT
NICU4-A19	China	III	2017-03-17	2017	Resistant	Susceptible	Susceptible	Urine	Clinical	SRR9316745	VF125AL	WT
NICU5-A36	China	III	2017-09-30	2017	Resistant	Susceptible	Susceptible	Urine	Clinical	SRR9316756	VF125AL	WT
NICU6-A41	China	III	2017-10-17	2017	Resistant	Susceptible	Susceptible	Fluid	Clinical	SRR9316762	VF125AL	WT
NICU7-A42	China	III	2017-11-22	2017	Resistant	Susceptible	Susceptible	Urine	Clinical	SRR9316761	VF125AL	WT
NICU8-A43	China	III	2017-11-24	2017	Resistant	Susceptible	Susceptible	Urine	Clinical	SRR9316760	VF125AL	WT
NICU9-A50	China	III	2017-12-12	2017	Resistant	Susceptible	Susceptible	Urine	Clinical	SRR9316770	VF125AL	WT
NSICU1-A24	China	III	2017-08-24	2017	Resistant	Susceptible	Susceptible	Urine	Clinical	SRR9316751	VF125AL	WT
NY-WCPHL-CAU-006553-(	United States	III	2023-01	2022	Unknown	Unknown	Unknown	Wound finger	Clinical	SRR24422757	VF125AL	WT
NY-WCPHL-CAU-052664	United States	III	2019-10-11	2019	Unknown	Unknown	Unknown	Peritoneal dialysis catheter	Clinical	SRR24621174	VF125AL	WT
NY-WCPHL-CAU-055098	United States	III	2022-11	2022	Unknown	Unknown	Unknown	Blood (peripheral)	Clinical	SRR24422751	VF125AL	WT
NY-WCPHL-CAU-244146	United States	III	2020-09-25	2020	Unknown	Unknown	Unknown	wound	Clinical	SRR24621161	VF125AL	WT
Patient-001	United Kingdom	III	2015-02-02	2015	Unknown	Unknown	Unknown	clinical sample	Clinical	SRR7976549	VF125AL	WT
Patient-002	United Kingdom	III	2015-07-10	2015	Unknown	Unknown	Unknown	clinical sample	Clinical	SRR7976579	VF125AL	WT
Patient-003	United Kingdom	III	2015-07-13	2015	Unknown	Unknown	Unknown	clinical sample	Clinical	SRR7976616	VF125AL	WT
Patient-004	United Kingdom	III	2015-11-24	2015	Unknown	Unknown	Unknown	clinical sample	Clinical	SRR7976582	VF125AL	WT

Patient-005	United Kingdom	III	2016-01-02	2016	Unknown	Unknown	Unknown	clinical sample	Clinical	SRR7976614	VF125AL	WT
Patient-006-01	United Kingdom	III	2016-10-25	2016	Unknown	Unknown	Unknown	clinical sample	Clinical	SRR7976557	VF125AL	WT
Patient-007	United Kingdom	III	2016-11-17	2016	Unknown	Unknown	Unknown	clinical sample	Clinical	SRR7976542	VF125AL	WT
Patient-008-01	United Kingdom	III	2016-11-28	2016	Unknown	Unknown	Unknown	clinical sample	Clinical	SRR7976584	VF125AL	WT
Patient-009-01	United Kingdom	III	2016-12-05	2016	Unknown	Unknown	Unknown	clinical sample	Clinical	SRR7976597	VF125AL	WT
Patient-010-01	United Kingdom	III	2016-12-11	2016	Unknown	Unknown	Unknown	clinical sample	Clinical	SRR7976591	VF125AL	WT
Patient-011-01	United Kingdom	III	2016-12-19	2016	Unknown	Unknown	Unknown	clinical sample	Clinical	SRR7976577	VF125AL	WT
Patient-012-01	United Kingdom	III	2017-01-09	2017	Unknown	Unknown	Unknown	clinical sample	Clinical	SRR7976604	VF125AL	WT
Patient-013-01	United Kingdom	III	2017-01-10	2017	Unknown	Unknown	Unknown	clinical sample	Clinical	SRR7976564	VF125AL	WT
Patient-015-01	United Kingdom	III	2017-01-25	2017	Unknown	Unknown	Unknown	clinical sample	Clinical	SRR7976599	VF125AL	WT
Patient-016	United Kingdom	III	2017-01-27	2017	Unknown	Unknown	Unknown	clinical sample	Clinical	SRR7976578	VF125AL	WT
Patient-017	United Kingdom	III	2017-02-10	2017	Unknown	Unknown	Unknown	clinical sample	Clinical	SRR7976540	VF125AL	WT
Patient-019-01	United Kingdom	III	2017-02-17	2017	Unknown	Unknown	Unknown	clinical sample	Clinical	SRR7976552	VF125AL	WT
Patient-021-01	United Kingdom	III	2017-03-08	2017	Unknown	Unknown	Unknown	clinical sample	Clinical	SRR7976548	VF125AL	WT
Patient-022-01	United Kingdom	III	2017-03-10	2017	Unknown	Unknown	Unknown	clinical sample	Clinical	SRR7976572	VF125AL	WT
Patient-023	United Kingdom	III	2017-03-18	2017	Unknown	Unknown	Unknown	clinical sample	Clinical	SRR7976553	VF125AL	WT
Patient-024-01	United Kingdom	III	2017-03-24	2017	Unknown	Unknown	Unknown	clinical sample	Clinical	SRR7976592	VF125AL	WT
Patient-025-01	United Kingdom	III	2017-03-27	2017	Unknown	Unknown	Unknown	clinical sample	Clinical	SRR7976565	VF125AL	WT
Patient-026	United Kingdom	III	2017-03-29	2017	Unknown	Unknown	Unknown	clinical sample	Clinical	SRR7976600	VF125AL	WT
Patient-027-01	United Kingdom	III	2017-03-31	2017	Unknown	Unknown	Unknown	clinical sample	Clinical	SRR7976615	VF125AL	WT
Patient-028-01	United Kingdom	III	2017-04-03	2017	Unknown	Unknown	Unknown	clinical sample	Clinical	SRR7976585	VF125AL	WT
Patient-029-01	United Kingdom	III	2017-04-07	2017	Unknown	Unknown	Unknown	clinical sample	Clinical	SRR7976558	VF125AL	WT
Patient-030	United Kingdom	III	2017-04-09	2017	Unknown	Unknown	Unknown	clinical sample	Clinical	SRR7976598	VF125AL	WT
Patient-031	United Kingdom	III	2017-04-10	2017	Unknown	Unknown	Unknown	clinical sample	Clinical	SRR7976607	VF125AL	WT
Patient-032	United Kingdom	III	2017-04-26	2017	Unknown	Unknown	Unknown	clinical sample	Clinical	SRR7976580	VF125AL	WT
Patient-033-01	United Kingdom	III	2017-05-05	2017	Unknown	Unknown	Unknown	clinical sample	Clinical	SRR7976541	VF125AL	WT
Patient-034	United Kingdom	III	2017-05-22	2017	Unknown	Unknown	Unknown	clinical sample	Clinical	SRR7976556	VF125AL	WT
Patient-037	United Kingdom	III	2017-04-24	2017	Unknown	Unknown	Unknown	clinical sample	Clinical	SRR7976603	VF125AL	WT
Patient-038	United Kingdom	III	2017-01-25	2017	Unknown	Unknown	Unknown	clinical sample	Clinical	SRR7976545	VF125AL	WT
RI-A112	China	III	2018-11-07	2018	Resistant	Susceptible	Susceptible	Urine	Clinical	SRR9316720	VF125AL	WT
RICU1-A1	China	III	2016-04-06	2016	Resistant	Susceptible	Susceptible	Urine	Clinical	SRR9316788	VF125AL	WT
RICU10-A57	China	III	2018-01-24	2018	Resistant	Susceptible	Susceptible	Tracheal catheter	Clinical	SRR9316775	VF125AL	WT
RICU11-A62	China	III	2018-03-02	2018	Resistant	Susceptible	Susceptible	Sputum	Clinical	SRR9316784	VF125AL	WT
RICU12-A80	China	III	2018-05-15	2018	Resistant	Susceptible	Susceptible	Sputum	Clinical	SRR9316798	VF125AL	WT
RICU13-A83	China	III	2018-05-31	2018	Resistant	Susceptible	Susceptible	Sputum	Clinical	SRR9316810	VF125AL	WT
RICU14-A95	China	III	2018-08-23	2018	Resistant	Susceptible	Susceptible	Urinary catheter	Clinical	SRR9316816	VF125AL	WT
RICU15-A105	China	III	2018-09-30	2018	Resistant	Susceptible	Susceptible	Sputum	Clinical	SRR9316795	VF125AL	WT
RICU16-A106	China	III	2018-10-05	2018	Resistant	Susceptible	Susceptible	Sputum	Clinical	SRR9316796	VF125AL	WT
RICU17-A108	China	III	2016-12-14	2018	Resistant	Susceptible	Susceptible	Blood	Clinical	SRR9316724	VF125AL	WT
RICU18-A113	China	III	2018-11-13	2018	Resistant	Susceptible	Susceptible	Urinary catheter	Clinical	SRR9316721	VF125AL	WT
RICU19-A115	China	III	2018-11-20	2018	Resistant	Susceptible	Susceptible	Sputum	Clinical	SRR9316723	VF125AL	WT
RICU2-A2	China	III	2016-06-03	2016	Resistant	Susceptible	Susceptible	Urine	Clinical	SRR9316744	VF125AL	WT
RICU3-A3	China	III	2016-06-17	2016	Resistant	Susceptible	Susceptible	Sputum	Clinical	SRR9316754	VF125AL	WT
RICU4-A7	China	III	2016-08-05	2016	Resistant	Susceptible	Susceptible	Sputum	Clinical	SRR9316779	VF125AL	WT
RICU5-A12	China	III	2016-12-07	2016	Resistant	Susceptible	Susceptible	Urinary catheter	Clinical	SRR9316729	VF125AL	WT
RICU6-A14	China	III	2017-01-26	2017	Resistant	Susceptible	Susceptible	Urinary catheter	Clinical	SRR9316738	VF125AL	WT
RICU7-A22	China	III	2017-07-29	2017	Resistant	Susceptible	Susceptible	Urine	Clinical	SRR9316746	VF125AL	WT
RICU8-A38	China	III	2017-10-10	2017	Resistant	Susceptible	Susceptible	Urine	Clinical	SRR9316765	VF125AL	WT
RICU9-A52	China	III	2017-12-31	2017	Resistant	Susceptible	Susceptible	Blood	Clinical	SRR9316772	VF125AL	WT
S1	Spain	III	2017-09-03	2017	Resistant	Unknown	Susceptible	Urinary catheter	Clinical	ERR12321083	VF125AL	WT
S103	Spain	III	2020-02-04	2020	Resistant	Unknown	Susceptible	Urinary catheter	Clinical	ERR12321104	VF125AL	WT
S117	Spain	III	2020-03-24	2020	Resistant	Unknown	Susceptible	Blood (venipuncture)	Clinical	ERR12321086	VF125AL	WT
S127	Spain	III	2020-04-14	2020	Resistant	Unknown	Susceptible	Axillary-rectal exudate	Clinical	ERR12321098	VF125AL	WT
S129	Spain	III	2020-05-04	2020	Resistant	Unknown	Susceptible	Blood (catheter)	Clinical	ERR12321077	VF125AL	WT
S131	Spain	III	2020-06-09	2020	Resistant	Unknown	Susceptible	Axillary-rectal exudate	Clinical	ERR12321100	VF125AL	WT
S132	Spain	III	2020-07-07	2020	Resistant	Unknown	Susceptible	Blood (catheter)	Clinical	ERR12321108	VF125AL	WT
S134	Spain	III	2020-08-17	2020	Resistant	Unknown	Susceptible	Axillary-rectal exudate	Clinical	ERR12321088	VF125AL	WT
S135	Spain	III	2020-09-09	2020	Resistant	Unknown	Susceptible	Blood (catheter)	Clinical	ERR12321091	VF125AL	WT
S138	Spain	III	2020-11-30	2020	Resistant	Unknown	Susceptible	Blood (catheter)	Clinical	ERR12321105	VF125AL	WT
S14	Spain	III	2018-02-06	2018	Resistant	Unknown	Susceptible	Blood (venipuncture)	Clinical	ERR12321082	VF125AL	WT
S140	Spain	III	2020-12-18	2020	Resistant	Unknown	Susceptible	Blood (catheter)	Clinical	ERR12321101	VF125AL	WT
S141	Spain	III	2021-01-12	2021	Resistant	Unknown	Susceptible	Axillary-rectal exudate	Clinical	ERR12321094	VF125AL	WT
S145	Spain	III	2021-02-11	2021	Resistant	Unknown	Susceptible	Blood (catheter)	Clinical	ERR12321107	VF125AL	WT
S147	Spain	III	2021-04-17	2021	Resistant	Unknown	Susceptible	Blood (catheter)	Clinical	ERR12321089	VF125AL	WT
S150	Spain	III	2021-05-12	2021	Resistant	Unknown	Susceptible	Blood (venipuncture)	Clinical	ERR12321093	VF125AL	WT
S151	Spain	III	2021-06-14	2021	Resistant	Unknown	Susceptible	Environmental	Environmental	ERR12321079	VF125AL	WT
S2	Spain	III	2017-10-14	2017	Resistant	Unknown	Susceptible	Blood (catheter)	Clinical	ERR12321099	VF125AL	WT
S28	Spain	III	2018-04-30	2018	Resistant	Unknown	Susceptible	Blood (catheter)	Clinical	ERR12321090	VF125AL	WT
S33	Spain	III	2018-05-27	2018	Resistant	Unknown	Susceptible	Blood (catheter)	Clinical	ERR12321092	VF125AL	WT
S35	Spain	III	2018-06-28	2018	Resistant	Unknown	Susceptible	Blood (venipuncture)	Clinical	ERR12321075	VF125AL	WT
S4	Spain	III	2017-10-18	2017	Resistant	Unknown	Susceptible	Blood (venipuncture)	Clinical	ERR12321102	VF125AL	WT
S44	Spain	III	2018-08-31	2018	Resistant	Unknown	Susceptible	Catheter	Clinical	ERR12321096	VF125AL	WT
S56	Spain	III	2018-11-13	2018	Resistant	Unknown	Susceptible	Lumbar puncture	Clinical	ERR12321097	VF125AL	WT
S62	Spain	III	2019-01-23	2019	Resistant	Unknown	Susceptible	Catheter	Clinical	ERR12321081	VF125AL	WT
S67	Spain	III	2019-02-22	2019	Resistant	Unknown	Resistant	Blood (catheter)	Clinical	ERR12321078	VF125AL	S639Y
S72	Spain	III	2019-04-02	2019	Resistant	Unknown	Susceptible	Urinary catheter	Clinical	ERR12321076	VF125AL	WT
S75	Spain	III	2019-05-21	2019	Resistant	Unknown	Susceptible	Blood (catheter)	Clinical	ERR12321103	VF125AL	WT
S77	Spain	III	2019-06-02	2019	Resistant	Unknown	Susceptible	Urine	Clinical	ERR12321087	VF125AL	WT
S8	Spain	III	2017-12-12	2017	Resistant	Unknown	Susceptible	Blood (catheter)	Clinical	ERR12321106	VF125AL	WT

S81	Spain	III	2019-08-16	2019	Resistant	Unknown	Susceptible	Blood (catheter)	Clinical	ERR12321085	VF125AL	WT
S82	Spain	III	2019-09-05	2019	Resistant	Unknown	Susceptible	Catheter tip	Clinical	ERR12321095	VF125AL	WT
S86	Spain	III	2019-10-18	2019	Resistant	Unknown	Susceptible	Blood (catheter)	Clinical	ERR12321084	VF125AL	WT
S94	Spain	III	2019-12-05	2019	Resistant	Unknown	Susceptible	Blood (catheter)	Clinical	ERR12321080	VF125AL	WT
S99	Spain	III	2020-01-29	2020	Resistant	Unknown	Susceptible	Blood (venipuncture)	Clinical	ERR12321074	VF125AL	WT
SICU1-A66	China	III	2018-04-06	2018	Resistant	Susceptible	Susceptible	Urine	Clinical	SRR9316780	VF125AL	WT
SICU2-A89	China	III	2018-07-25	2018	Resistant	Susceptible	Susceptible	Sputum	Clinical	SRR9316815	VF125AL	WT
TempProbe1	United Kingdom	III	2017-04-04	2017	Unknown	Unknown	Unknown	Environmental	Environmental	SRR7976593	VF125AL	WT
TempProbe2	United Kingdom	III	2017-05-16	2017	Unknown	Unknown	Unknown	Environmental	Environmental	SRR7976601	VF125AL	WT
TempProbe3	United Kingdom	III	2017-05-16	2017	Unknown	Unknown	Unknown	Environmental	Environmental	SRR7976602	VF125AL	WT
TempProbe4	United Kingdom	III	2017-05-16	2017	Unknown	Unknown	Unknown	Environmental	Environmental	SRR7976571	VF125AL	WT
TempProbe5	United Kingdom	III	2017-05-16	2017	Unknown	Unknown	Unknown	Environmental	Environmental	SRR7976560	VF125AL	WT
UCLA-A1	United States	III	2019-10-08	2019	Resistant	Resistant	Susceptible	inguinal/axillary swab	Clinical	SRR12916694	VF125AL	WT
UCLA-A2	United States	III	2019-10-08	2019	Resistant	Resistant	Susceptible	Trachea	Clinical	SRR12916693	VF125AL	WT
UCLA-C1	United States	III	2020-08-12	2020	Resistant	Resistant	Susceptible	inguinal/axillary swab	Clinical	SRR12916692	VF125AL	WT
UCLA-D1	United States	III	2020-08-19	2020	Resistant	Resistant	Susceptible	inguinal/axillary swab	Clinical	SRR12916691	VF125AL	WT
WM-18-176	Australia	III	2015	2015	Resistant	Susceptible	Susceptible	Sternal bone	Clinical	SRR11485318	VF125AL	WT
WM-18-177	South Africa	III	2018-07	2018	Resistant	Susceptible	Susceptible	Blood culture	Clinical	SRR11485317	VF125AL	WT
WM-18-178	South Africa	III	2017	2017	Resistant	Susceptible	Susceptible	Blood culture	Clinical	SRR11485332	VF125AL	WT
WM-18-179	South Africa	III	2017	2017	Resistant	Susceptible	Susceptible	Blood culture	Clinical	SRR11485331	VF125AL	WT
WM-18-180	Australia	III	2018-09-20	2018	Resistant	Susceptible	Susceptible	nose swab	Clinical	SRR11485320	VF125AL	WT
WM-18-181	Australia	III	2018-09-05	2018	Resistant	Susceptible	Susceptible	Wound swab PICC site	Clinical	SRR11485334	VF125AL	WT
WM-18-182	Australia	III	2018-09-05	2018	Resistant	Susceptible	Susceptible	sputum	Clinical	SRR11485333	VF125AL	WT
WM-18-187	Australia	III	2018-09-06	2018	Resistant	Susceptible	Susceptible	Axilla swab	Clinical	SRR11485324	VF125AL	WT
WM-18-188	Australia	III	2018-09-07	2018	Resistant	Susceptible	Susceptible	sputum	Clinical	SRR11485323	VF125AL	WT
WM-18-189	Australia	III	2018-09-11	2018	Resistant	Susceptible	Susceptible	Axilla swab	Clinical	SRR11485322	VF125AL	WT
WM-18-190	Australia	III	2018-09-11	2018	Resistant	Susceptible	Susceptible	Groin	Clinical	SRR11485321	VF125AL	WT
WM-18-197	Australia	III	2018-11-08	2018	Resistant	Susceptible	Susceptible	throat	Clinical	SRR11485319	VF125AL	WT
ACEGID-C2	Nigeria	IV	2021-02	2021	Unknown	Unknown	Unknown	blood	Clinical	SRR19214214	WT	WT
ACEGID-C3	Nigeria	IV	2021-03	2021	Unknown	Unknown	Unknown	blood	Clinical	SRR19214213	WT	WT
B11892	Israel	IV	2014-10-15	2014	Resistant	Susceptible	Susceptible	Blood	Clinical	SRR10461259	WT	WT
B11896	Israel	IV	2015-04-15	2015	Susceptible	Susceptible	Susceptible	Urine	Clinical	SRR10461255	WT	WT
B11897	Israel	IV	2015-04-15	2015	Resistant	Susceptible	Susceptible	Blood	Clinical	SRR10461254	WT	WT
B12094	Panama	IV	2016-09-22	2016	Resistant	Susceptible	Susceptible	Urine	Clinical	SRR10461252	WT	WT
B12101	Panama	IV	2016-09-22	2016	Susceptible	Susceptible	Susceptible	Urine	Clinical	SRR10461183	WT	WT
B12106	Panama	IV	2016-09-21	2016	Susceptible	Susceptible	Susceptible	Urine	Clinical	SRR10461179	WT	WT
B12186	Venezuela	IV	2016-09-28	2016	Resistant	Susceptible	Susceptible	Catheter tip	Clinical	SRR10461195	Y132F	WT
B12187	Venezuela	IV	2016-09-30	2016	Resistant	Susceptible	Susceptible	Blood	Clinical	SRR10461194	Y132F	WT
B12188	Venezuela	IV	2016-06-10	2016	Resistant	Susceptible	Susceptible	Catheter tip	Clinical	SRR10461193	Y132F	WT
B12244	Colombia	IV	2016-12-15	2016	Susceptible	Susceptible	Susceptible	Environmental	Environmental	SRR10461192	WT	WT
B12279	Colombia	IV	2016-09-30	2016	Susceptible	Susceptible	Susceptible	Rectum swab	Clinical	SRR10461189	WT	WT
B12284	Colombia	IV	2016-12-15	2016	Susceptible	Susceptible	Susceptible	Axilla swab	Clinical	SRR10461188	WT	WT
B12388	United States	IV	2016-09-25	2016	Susceptible	Susceptible	Susceptible	Axilla/Groin swab	Clinical	SRR7909221	WT	WT
B12406	United States	IV	2016-12-27	2016	Susceptible	Susceptible	Susceptible	Urine	Clinical	SRR7909405	WT	WT
B12847	United States	IV	2017	2017	Unknown	Unknown	Unknown	urine	Clinical	SRR12784142	Y132F	S639F
B12847	United States	IV	2017-05-04	2017	Unknown	Unknown	Unknown	urine	Clinical	SRR7909414	WT	WT
B12938	United States	IV	2017-04-17	2017	Susceptible	Susceptible	Susceptible	Axilla/Groin swab	Clinical	SRR7909357	WT	WT
B13699	United States	IV	2017	2017	Unknown	Unknown	Unknown	wound	Clinical	SRR12784141	WT	WT
B13700	United States	IV	2017	2017	Unknown	Unknown	Unknown	wound	Clinical	SRR12784130	WT	WT
B13701	United States	IV	2017	2017	Unknown	Unknown	Unknown	wound	Clinical	SRR12784125	WT	WT
B13702	United States	IV	2017	2017	Unknown	Unknown	Unknown	wound	Clinical	SRR12784124	WT	WT
B13703	United States	IV	2017	2017	Unknown	Unknown	Unknown	blood	Clinical	SRR12073466	WT	WT
B16329	United States	IV	2018	2018	Unknown	Unknown	Unknown	blood	Clinical	SRR12073454	WT	WT
GCA 003014415.1	Venezuela	IV	2013-03-23	2013	Resistant	Susceptible	Unknown	Blood	Clinical	Reference assembly	Y132F	WT
MRL 2723	South Africa	IV	2017	2017	Unknown	Unknown	Unknown	Blood	Clinical	SRR14802837	WT	WT
MRL 353	South Africa	IV	2009	2009	Unknown	Unknown	Unknown	Blood	Clinical	SRR14802929	WT	WT
MRL 4916	South Africa	IV	2017	2017	Unknown	Unknown	Unknown	Blood	Clinical	SRR14802909	WT	WT
N19-00-135	Canada	IV	2015	2015	Susceptible	Susceptible	Susceptible	Peritoneal fluid	Clinical	SRR15192303	WT	WT
NG-20724Am-01	Netherlands	IV	2019	2019	Resistant	Susceptible	Susceptible	Urine	Clinical	SRR9993948	WT	WT
IFRC2087	Iran	V	2018	2018	Susceptible	Susceptible	Susceptible	Ear swab	Clinical	SRR9007776	WT	WT

Supplementary Table 3. Metadata (sample origin, phenotypic and genotypic characteristics of resistance-related mutations) of all *Candida auris* Clade III genomes analyzed in this study (n=566).

Sample	Country	City/State	hylogenetic clad	clade3 group	Collection date	Collection year	Fluconazole (FLC)	Amphotericin B (AMB)	Micafungin (MFG)	Specimen source	SRA run	ERG11 (FLC)	FKS1 (MFG)
B17721	United States	California	III	clade3 group3	2019-02-17	2019	Unknown	Unknown	Unknown	urine	SRR17577117	VF125AL	WT
B17741	United States	California	III	clade3 group3	2019-03-14	2019	Unknown	Unknown	Unknown	axilla and groin	SRR17577116	VF125AL	WT
B17742	United States	California	III	clade3 group3	2019-03-14	2019	Unknown	Unknown	Unknown	axilla and groin	SRR17577105	VF125AL	WT
B17743	United States	California	III	clade3 group3	2019-03-14	2019	Unknown	Unknown	Unknown	axilla and groin	SRR17577094	VF125AL	WT
B17746	United States	California	III	clade3 group3	2019-03-14	2019	Unknown	Unknown	Unknown	axilla and groin	SRR17577083	VF125AL	WT
B17806	United States	California	III	clade3 group3	2019-03-28	2019	Unknown	Unknown	Unknown	not applicable	SRR17577060	VF125AL	WT
B17853	United States	California	III	clade3 group3	2019-04-03	2019	Unknown	Unknown	Unknown	axilla and groin	SRR17577071	VF125AL	WT
B17854	United States	California	III	clade3 group3	2019-04-03	2019	Unknown	Unknown	Unknown	axilla and groin	SRR17577070	VF125AL	WT
B17855	United States	California	III	clade3 group3	2019-04-03	2019	Unknown	Unknown	Unknown	axilla and groin	SRR17577115	VF125AL	WT
B17873	United States	California	III	clade3 group3	2019-04-03	2019	Unknown	Unknown	Unknown	nose	SRR17577096	VF125AL	WT
B17874	United States	California	III	clade3 group3	2019-04-03	2019	Unknown	Unknown	Unknown	nose	SRR17577095	VF125AL	WT
B17856	United States	California	III	clade3 group3	2019-04-04	2019	Unknown	Unknown	Unknown	axilla and groin	SRR17577114	VF125AL	WT
B17857	United States	California	III	clade3 group3	2019-04-08	2019	Unknown	Unknown	Unknown	nose	SRR17577113	VF125AL	WT
B17858	United States	California	III	clade3 group3	2019-04-08	2019	Unknown	Unknown	Unknown	axilla and groin	SRR17577112	VF125AL	WT
B17859	United States	California	III	clade3 group3	2019-04-08	2019	Unknown	Unknown	Unknown	axilla and groin	SRR17577111	VF125AL	WT
B17860	United States	California	III	clade3 group3	2019-04-08	2019	Unknown	Unknown	Unknown	axilla and groin	SRR17577110	VF125AL	WT
B17861	United States	California	III	clade3 group3	2019-04-08	2019	Unknown	Unknown	Unknown	axilla and groin	SRR17577109	VF125AL	WT
B17862	United States	California	III	clade3 group3	2019-04-08	2019	Unknown	Unknown	Unknown	axilla	SRR17577108	VF125AL	WT
B17863	United States	California	III	clade3 group3	2019-04-08	2019	Unknown	Unknown	Unknown	nose	SRR17577107	VF125AL	WT
B17864	United States	California	III	clade3 group3	2019-04-08	2019	Unknown	Unknown	Unknown	nose	SRR17577106	VF125AL	WT
B17865	United States	California	III	clade3 group3	2019-04-08	2019	Unknown	Unknown	Unknown	axilla and groin	SRR17577104	VF125AL	WT
B17866	United States	California	III	clade3 group3	2019-04-08	2019	Unknown	Unknown	Unknown	axilla and groin	SRR17577103	VF125AL	WT
B17867	United States	California	III	clade3 group3	2019-04-08	2019	Unknown	Unknown	Unknown	axilla	SRR17577102	VF125AL	WT
B17868	United States	California	III	clade3 group3	2019-04-08	2019	Unknown	Unknown	Unknown	axilla and groin	SRR17577101	VF125AL	WT
B17869	United States	California	III	clade3 group3	2019-04-08	2019	Unknown	Unknown	Unknown	axilla and groin	SRR17577100	VF125AL	WT
B17870	United States	California	III	clade3 group3	2019-04-08	2019	Unknown	Unknown	Unknown	axilla and groin	SRR17577099	VF125AL	WT
B17871	United States	California	III	clade3 group3	2019-04-11	2019	Unknown	Unknown	Unknown	axilla and groin	SRR17577098	VF125AL	WT
B17872	United States	California	III	clade3 group3	2019-04-11	2019	Unknown	Unknown	Unknown	nose	SRR17577097	VF125AL	WT
C20955	United States	California	III	clade3 group3	2019-04-18	2019	Unknown	Unknown	Unknown	axilla and groin	SRR17577053	VF125AL	WT
B17833	United States	California	III	clade3 group3	2019-04-22	2019	Unknown	Unknown	Unknown	nose	SRR17577049	VF125AL	WT
B17834	United States	California	III	clade3 group3	2019-04-22	2019	Unknown	Unknown	Unknown	nose	SRR17577038	VF125AL	WT
C21072	United States	California	III	clade3 group3	2019-04-22	2019	Unknown	Unknown	Unknown	axilla and groin	SRR17577052	VF125AL	WT
C21086	United States	California	III	clade3 group3	2019-04-22	2019	Unknown	Unknown	Unknown	axilla and groin	SRR17577051	VF125AL	WT
C21485	United States	California	III	clade3 group3	2019-04-25	2019	Unknown	Unknown	Unknown	axilla and groin	SRR17577050	VF125AL	WT
B17924	United States	California	III	clade3 group3	2019-05-04	2019	Unknown	Unknown	Unknown	blood	SRR17577088	VF125AL	WT
B17875	United States	California	III	clade3 group3	2019-05-07	2019	Unknown	Unknown	Unknown	nose	SRR17577093	VF125AL	WT
B17876	United States	California	III	clade3 group3	2019-05-07	2019	Unknown	Unknown	Unknown	nose	SRR17577092	VF125AL	WT
B17916	United States	California	III	clade3 group3	2019-05-07	2019	Unknown	Unknown	Unknown	bronchial washings	SRR17577089	VF125AL	WT
C45587	United States	California	III	clade3 group3	2019-05-07	2019	Unknown	Unknown	Unknown	axilla and groin	SRR17577048	VF125AL	WT
C45616	United States	California	III	clade3 group3	2019-05-07	2019	Unknown	Unknown	Unknown	axilla and groin	SRR17577047	VF125AL	WT
C45954	United States	California	III	clade3 group3	2019-05-08	2019	Unknown	Unknown	Unknown	axilla and groin	SRR17577046	VF125AL	WT
C45960	United States	California	III	clade3 group3	2019-05-08	2019	Unknown	Unknown	Unknown	axilla and groin	SRR17577045	VF125AL	WT
C45964	United States	California	III	clade3 group3	2019-05-08	2019	Unknown	Unknown	Unknown	axilla and groin	SRR17577044	VF125AL	WT
C45965	United States	California	III	clade3 group3	2019-05-08	2019	Unknown	Unknown	Unknown	axilla and groin	SRR17577043	VF125AL	WT
C45969	United States	California	III	clade3 group3	2019-05-08	2019	Unknown	Unknown	Unknown	axilla and groin	SRR17577042	VF125AL	WT
C45991	United States	California	III	clade3 group3	2019-05-08	2019	Unknown	Unknown	Unknown	axilla and groin	SRR17577041	VF125AL	WT
C46002	United States	California	III	clade3 group3	2019-05-08	2019	Unknown	Unknown	Unknown	axilla and groin	SRR17577040	VF125AL	WT
C46008	United States	California	III	clade3 group3	2019-05-08	2019	Unknown	Unknown	Unknown	axilla and groin	SRR17577039	VF125AL	WT
C46010	United States	California	III	clade3 group3	2019-05-08	2019	Unknown	Unknown	Unknown	axilla and groin	SRR17577081	VF125AL	WT
C46013	United States	California	III	clade3 group3	2019-05-08	2019	Unknown	Unknown	Unknown	axilla and groin	SRR17577080	VF125AL	WT
C46014	United States	California	III	clade3 group3	2019-05-08	2019	Unknown	Unknown	Unknown	axilla and groin	SRR17577079	VF125AL	WT
C46015	United States	California	III	clade3 group3	2019-05-08	2019	Unknown	Unknown	Unknown	axilla and groin	SRR17577078	VF125AL	WT
C46020	United States	California	III	clade3 group3	2019-05-08	2019	Unknown	Unknown	Unknown	axilla and groin	SRR17577077	VF125AL	WT
C46022	United States	California	III	clade3 group3	2019-05-08	2019	Unknown	Unknown	Unknown	axilla and groin	SRR17577076	VF125AL	WT
C46027	United States	California	III	clade3 group3	2019-05-08	2019	Unknown	Unknown	Unknown	axilla and groin	SRR17577075	VF125AL	WT
B17877	United States	California	III	clade3 group3	2019-05-09	2019	Unknown	Unknown	Unknown	nose	SRR17577091	VF125AL	WT
B17878	United States	California	III	clade3 group3	2019-05-09	2019	Unknown	Unknown	Unknown	nose	SRR17577090	VF125AL	WT
C46062	United States	California	III	clade3 group3	2019-05-09	2019	Unknown	Unknown	Unknown	axilla and groin	SRR17577074	VF125AL	WT
C46107	United States	California	III	clade3 group3	2019-05-09	2019	Unknown	Unknown	Unknown	axilla and groin	SRR17577073	VF125AL	WT
C46121	United States	California	III	clade3 group3	2019-05-09	2019	Unknown	Unknown	Unknown	axilla and groin	SRR17577072	VF125AL	WT
B17948	United States	California	III	clade3 group3	2019-05-15	2019	Unknown	Unknown	Unknown	axilla and groin	SRR17577086	VF125AL	WT
B17949	United States	California	III	clade3 group3	2019-05-15	2019	Unknown	Unknown	Unknown	axilla and groin	SRR17577085	VF125AL	WT
B17950	United States	California	III	clade3 group3	2019-05-15	2019	Unknown	Unknown	Unknown	axilla and groin	SRR17577084	VF125AL	WT
B17937	United States	California	III	clade3 group3	2019-05-29	2019	Unknown	Unknown	Unknown	axilla and groin	SRR17577087	VF125AL	WT
B18157	United States	California	III	clade3 group3	2019-07-04	2019	Unknown	Unknown	Unknown	urine	SRR17577082	VF125AL	WT

B18158	United States	California	III	clade3 group3	2019-07-07	2019	Unknown	Unknown	Unknown	foreign object (body)	SRR17577069	VF125AL	WT
B18160	United States	California	III	clade3 group3	2019-07-15	2019	Unknown	Unknown	Unknown	blood	SRR17577067	VF125AL	WT
B18159	United States	California	III	clade3 group3	2019-07-17	2019	Unknown	Unknown	Unknown	axilla and groin	SRR17577068	VF125AL	WT
B18225	United States	California	III	clade3 group3	2019-07-17	2019	Unknown	Unknown	Unknown	urine	SRR17577066	VF125AL	WT
B18227	United States	California	III	clade3 group3	2019-07-24	2019	Unknown	Unknown	Unknown	urine	SRR17577064	VF125AL	WT
B18226	United States	California	III	clade3 group3	2019-07-29	2019	Unknown	Unknown	Unknown	sputum	SRR17577065	VF125AL	WT
CA-OCPHL-CA	United States	California	III	clade3 group3	2019-07-29	2019	Unknown	Unknown	Unknown	sputum	SRR24806737	VF125AL	WT
B18249	United States	California	III	clade3 group3	2019-08-13	2019	Unknown	Unknown	Unknown	axilla and groin	SRR17577063	VF125AL	WT
B18461	United States	California	III	clade3 group3	2019-08-17	2019	Unknown	Unknown	Unknown	ear	SRR17577056	VF125AL	WT
B18289	United States	California	III	clade3 group3	2019-08-22	2019	Unknown	Unknown	Unknown	blood	SRR17577059	VF125AL	WT
B18263	United States	California	III	clade3 group3	2019-08-28	2019	Unknown	Unknown	Unknown	axilla and groin	SRR17577062	VF125AL	WT
B18460	United States	California	III	clade3 group3	2019-09-01	2019	Unknown	Unknown	Unknown	blood	SRR17577057	VF125AL	WT
B18278	United States	California	III	clade3 group3	2019-09-11	2019	Unknown	Unknown	Unknown	axilla and groin	SRR17577061	VF125AL	WT
B18540	United States	California	III	clade3 group3	2019-09-15	2019	Unknown	Unknown	Unknown	blood	SRR17577055	VF125AL	WT
B18458	United States	California	III	clade3 group3	2019-09-25	2019	Unknown	Unknown	Unknown	axilla and groin	SRR17577058	VF125AL	WT
B18657	United States	California	III	clade3 group3	2019-10-01	2019	Unknown	Unknown	Unknown	axilla and groin	SRR17577054	VF125AL	WT
UCLA-A1	United States	California	III	clade3 group3	2019-10-08	2019	Resistant	Resistant	Susceptible	inguinal/axillary swab	SRR12916694	VF125AL	WT
UCLA-A2	United States	California	III	clade3 group3	2019-10-08	2019	Resistant	Resistant	Susceptible	Trachea	SRR12916693	VF125AL	WT
CA-OCPHL-CA	United States	California	III	clade3 group3	2020-05-24	2020	Unknown	Unknown	Unknown	urine	SRR24806768	VF125AL	WT
UCLA-C1	United States	California	III	clade3 group3	2020-08-12	2020	Resistant	Resistant	Susceptible	inguinal/axillary swab	SRR12916692	VF125AL	WT
UCLA-D1	United States	California	III	clade3 group3	2020-08-19	2020	Resistant	Resistant	Susceptible	inguinal/axillary swab	SRR12916691	VF125AL	WT
CA-OCPHL-CA	United States	California	III	clade3 group3	2021-04-27	2021	Unknown	Unknown	Unknown	blood	SRR24806704	VF125AL	WT
CA-OCPHL-CA	United States	California	III	clade3 group3	2021-05-07	2021	Unknown	Unknown	Unknown	urine	SRR24806760	VF125AL	S639F
CA-OCPHL-CA	United States	California	III	clade3 group3	2021-05-12	2021	Unknown	Unknown	Unknown	urine	SRR24806759	VF125AL	WT
CA-OCPHL-CA	United States	California	III	clade3 group3	2021-05-28	2021	Unknown	Unknown	Unknown	body fluid	SRR24806693	VF125AL	WT
CA-OCPHL-CA	United States	California	III	clade3 group3	2021-06-26	2021	Unknown	Unknown	Unknown	tracheal aspirate	SRR24806708	VF125AL	WT
CA-OCPHL-CA	United States	California	III	clade3 group3	2021-07-14	2021	Unknown	Unknown	Unknown	Axilla/Groin	SRR24806751	VF125AL	WT
CA-OCPHL-CA	United States	California	III	clade3 group3	2021-07-19	2021	Unknown	Unknown	Unknown	Resp Processed	SRR24806707	VF125AL	WT
CA-OCPHL-CA	United States	California	III	clade3 group3	2021-07-30	2021	Unknown	Unknown	Unknown	urine	SRR24806763	VF125AL	WT
CA-OCPHL-CA	United States	California	III	clade3 group3	2021-07-31	2021	Unknown	Unknown	Unknown	wound	SRR24806706	VF125AL	WT
CA-OCPHL-CA	United States	California	III	clade3 group3	2021-08-28	2021	Unknown	Unknown	Unknown	Axilla/Groin	SRR24806692	VF125AL	WT
CA-OCPHL-CA	United States	California	III	clade3 group3	2021-09-03	2021	Unknown	Unknown	Unknown	wound	SRR24806764	VF125AL	WT
CA-OCPHL-CA	United States	California	III	clade3 group3	2021-09-11	2021	Unknown	Unknown	Unknown	tracheal aspirate	SRR24806705	VF125AL	WT
CA-OCPHL-CA	United States	California	III	clade3 group3	2021-09-14	2021	Unknown	Unknown	Unknown	Axilla/Groin	SRR24806673	VF125AL	WT
CA-OCPHL-CA	United States	California	III	clade3 group3	2021-09-17	2021	Unknown	Unknown	Unknown	Axilla/Groin	SRR24806691	VF125AL	WT
CA-OCPHL-CA	United States	California	III	clade3 group3	2021-09-28	2021	Unknown	Unknown	Unknown	urine	SRR24806761	VF125AL	WT
CA-OCPHL-CA	United States	California	III	clade3 group3	2021-10-01	2021	Unknown	Unknown	Unknown	wound	SRR24806690	VF125AL	WT
CA-OCPHL-CA	United States	California	III	clade3 group3	2021-10-11	2021	Unknown	Unknown	Unknown	urine	SRR24806703	VF125AL	WT
CA-OCPHL-CA	United States	California	III	clade3 group3	2021-10-20	2021	Unknown	Unknown	Unknown	blood	SRR24806767	VF125AL	WT
CA-OCPHL-CA	United States	California	III	clade3 group3	2021-10-21	2021	Unknown	Unknown	Unknown	urine	SRR24806702	VF125AL	WT
CA-OCPHL-CA	United States	California	III	clade3 group3	2021-10-29	2021	Unknown	Unknown	Unknown	urine	SRR24806701	VF125AL	WT
CA-OCPHL-CA	United States	California	III	clade3 group3	2021-11-11	2021	Unknown	Unknown	Unknown	Tissue	SRR24806715	VF125AL	WT
CA-OCPHL-CA	United States	California	III	clade3 group3	2021-11-17	2021	Unknown	Unknown	Unknown	urine	SRR24806689	VF125AL	WT
CA-OCPHL-CA	United States	California	III	clade3 group3	2021-11-20	2021	Unknown	Unknown	Unknown	urine	SRR24806766	VF125AL	WT
CA-OCPHL-CA	United States	California	III	clade3 group3	2021-11-22	2021	Unknown	Unknown	Unknown	Axilla/Groin	SRR24806688	VF125AL	WT
CA-OCPHL-CA	United States	California	III	clade3 group3	2021-12-03	2021	Unknown	Unknown	Unknown	blood	SRR24806700	VF125AL	WT
CA-OCPHL-CA	United States	California	III	clade3 group3	2021-12-16	2021	Unknown	Unknown	Unknown	urine	SRR24806748	VF125AL	WT
CA-OCPHL-CA	United States	California	III	clade3 group3	2021-12-17	2021	Unknown	Unknown	Unknown	blood	SRR24806699	VF125AL	WT
CA-OCPHL-CA	United States	California	III	clade3 group3	2021-12-18	2021	Unknown	Unknown	Unknown	wound	SRR24806722	VF125AL	WT
CA-OCPHL-CA	United States	California	III	clade3 group3	2021-12-29	2021	Unknown	Unknown	Unknown	Catheter	SRR24806698	VF125AL	WT
CA-OCPHL-CA	United States	California	III	clade3 group3	2022-01-02	2022	Unknown	Unknown	Unknown	blood	SRR24806783	VF125AL	WT
CA-OCPHL-CA	United States	California	III	clade3 group3	2022-01-03	2022	Unknown	Unknown	Unknown	wound	SRR24806755	VF125AL	WT
CA-OCPHL-CA	United States	California	III	clade3 group3	2022-01-09	2022	Unknown	Unknown	Unknown	blood	SRR24806682	VF125AL	WT
CA-OCPHL-CA	United States	California	III	clade3 group3	2022-02-08	2022	Unknown	Unknown	Unknown	urine	SRR24806687	VF125AL	WT
CA-OCPHL-CA	United States	California	III	clade3 group3	2022-02-12	2022	Unknown	Unknown	Unknown	blood	SRR24806686	VF125AL	WT
CA-OCPHL-CA	United States	California	III	clade3 group3	2022-02-15	2022	Unknown	Unknown	Unknown	Axilla/Groin	SRR24806744	VF125AL	WT
CA-OCPHL-CA	United States	California	III	clade3 group3	2022-02-24	2022	Unknown	Unknown	Unknown	urine	SRR24806697	VF125AL	WT
CA-OCPHL-CA	United States	California	III	clade3 group3	2022-02-27	2022	Unknown	Unknown	Unknown	urine	SRR24806721	VF125AL	WT
CA-OCPHL-CA	United States	California	III	clade3 group3	2022-03-01	2022	Unknown	Unknown	Unknown	urine	SRR24806685	VF125AL	WT
CA-OCPHL-CA	United States	California	III	clade3 group3	2022-03-28	2022	Unknown	Unknown	Unknown	Axilla/Groin	SRR24806729	VF125AL	WT
CA-OCPHL-CA	United States	California	III	clade3 group3	2022-03-28	2022	Unknown	Unknown	Unknown	Axilla/Groin	SRR24806739	VF125AL	WT
CA-OCPHL-CA	United States	California	III	clade3 group3	2022-03-28	2022	Unknown	Unknown	Unknown	Axilla/Groin	SRR24806746	VF125AL	WT
CA-OCPHL-CA	United States	California	III	clade3 group3	2022-04-08	2022	Unknown	Unknown	Unknown	wound	SRR24806725	VF125AL	WT
CA-OCPHL-CA	United States	California	III	clade3 group3	2022-04-15	2022	Unknown	Unknown	Unknown	blood	SRR24806696	VF125AL	WT
CA-OCPHL-CA	United States	California	III	clade3 group3	2022-04-27	2022	Unknown	Unknown	Unknown	blood	SRR24806695	VF125AL	WT
CA-OCPHL-CA	United States	California	III	clade3 group3	2022-05-10	2022	Unknown	Unknown	Unknown	BAL	SRR24806757	VF125AL	WT
CA-OCPHL-CA	United States	California	III	clade3 group3	2022-05-13	2022	Unknown	Unknown	Unknown	wound	SRR24806684	VF125AL	WT

CA-OCPHL-CA	United States	California	III	clade3 group3	2022-05-19	2022	Unknown	Unknown	Unknown	Axilla/Groin	SRR24806730	VF125AL	WT
CA-OCPHL-CA	United States	California	III	clade3 group3	2022-05-26	2022	Unknown	Unknown	Unknown	Axilla/Groin	SRR24806683	VF125AL	WT
CA-OCPHL-CA	United States	California	III	clade3 group3	2022-05-28	2022	Unknown	Unknown	Unknown	urine	SRR24806720	VF125AL	WT
CA-OCPHL-CA	United States	California	III	clade3 group3	2022-05-30	2022	Unknown	Unknown	Unknown	Tissue	SRR24806681	VF125AL	WT
CA-OCPHL-CA	United States	California	III	clade3 group3	2022-06-01	2022	Unknown	Unknown	Unknown	Bronch Wash	SRR24806680	VF125AL	WT
CA-OCPHL-CA	United States	California	III	clade3 group3	2022-06-03	2022	Unknown	Unknown	Unknown	wound	SRR24806747	VF125AL	WT
CA-OCPHL-CA	United States	California	III	clade3 group3	2022-06-07	2022	Unknown	Unknown	Unknown	wound	SRR24806750	VF125AL	WT
CA-OCPHL-CA	United States	California	III	clade3 group3	2022-06-16	2022	Unknown	Unknown	Unknown	sputum	SRR24806758	VF125AL	WT
CA-OCPHL-CA	United States	California	III	clade3 group3	2022-06-20	2022	Unknown	Unknown	Unknown	Axilla/Groin	SRR24806732	VF125AL	WT
CA-OCPHL-CA	United States	California	III	clade3 group3	2022-07-01	2022	Unknown	Unknown	Unknown	urine	SRR24806765	VF125AL	WT
CA-OCPHL-CA	United States	California	III	clade3 group3	2022-07-01	2022	Unknown	Unknown	Unknown	Axilla/Groin	SRR24806679	VF125AL	WT
CA-OCPHL-CA	United States	California	III	clade3 group3	2022-07-08	2022	Unknown	Unknown	Unknown	urine	SRR24806678	VF125AL	WT
CA-OCPHL-CA	United States	California	III	clade3 group3	2022-07-09	2022	Unknown	Unknown	Unknown	body fluid	SRR24806754	VF125AL	WT
CA-OCPHL-CA	United States	California	III	clade3 group3	2022-07-11	2022	Unknown	Unknown	Unknown	Respiratory Process	SRR24806709	VF125AL	WT
CA-OCPHL-CA	United States	California	III	clade3 group3	2022-07-15	2022	Unknown	Unknown	Unknown	urine	SRR24806718	VF125AL	WT
CA-OCPHL-CA	United States	California	III	clade3 group3	2022-08-06	2022	Unknown	Unknown	Unknown	blood	SRR24806784	VF125AL	WT
CA-OCPHL-CA	United States	California	III	clade3 group3	2022-08-25	2022	Unknown	Unknown	Unknown	urine	SRR24806752	VF125AL	WT
CA-OCPHL-CA	United States	California	III	clade3 group3	2022-08-27	2022	Unknown	Unknown	Unknown	Axilla/Groin	SRR24806677	VF125AL	WT
CA-OCPHL-CA	United States	California	III	clade3 group3	2022-09-06	2022	Unknown	Unknown	Unknown	Axilla/Groin	SRR24806727	VF125AL	WT
CA-OCPHL-CA	United States	California	III	clade3 group3	2022-09-07	2022	Unknown	Unknown	Unknown	sputum	SRR24806676	VF125AL	WT
CA-OCPHL-CA	United States	California	III	clade3 group3	2022-09-13	2022	Unknown	Unknown	Unknown	Axilla/Groin	SRR24806735	VF125AL	WT
CA-OCPHL-CA	United States	California	III	clade3 group3	2022-09-20	2022	Unknown	Unknown	Unknown	urine	SRR24806710	VF125AL	WT
CA-OCPHL-CA	United States	California	III	clade3 group3	2022-09-28	2022	Unknown	Unknown	Unknown	Peg site	SRR24806724	VF125AL	WT
CA-OCPHL-CA	United States	California	III	clade3 group3	2022-09-30	2022	Unknown	Unknown	Unknown	blood	SRR24806694	VF125AL	WT
CA-OCPHL-CA	United States	California	III	clade3 group3	2022-10-07	2022	Unknown	Unknown	Unknown	blood	SRR24806749	VF125AL	WT
CA-OCPHL-CA	United States	California	III	clade3 group3	2022-10-13	2022	Unknown	Unknown	Unknown	Tissue	SRR24806726	VF125AL	WT
CA-OCPHL-CA	United States	California	III	clade3 group3	2022-10-19	2022	Unknown	Unknown	Unknown	Axilla/Groin	SRR24806742	VF125AL	WT
CA-OCPHL-CA	United States	California	III	clade3 group3	2022-10-19	2022	Unknown	Unknown	Unknown	Axilla/Groin	SRR24806736	VF125AL	WT
CA-OCPHL-CA	United States	California	III	clade3 group3	2022-10-19	2022	Unknown	Unknown	Unknown	wound	SRR24806717	VF125AL	WT
CA-OCPHL-CA	United States	California	III	clade3 group3	2022-10-23	2022	Unknown	Unknown	Unknown	urine	SRR24806712	VF125AL	WT
CA-OCPHL-CA	United States	California	III	clade3 group3	2022-10-26	2022	Unknown	Unknown	Unknown	Axilla/Groin	SRR24806728	VF125AL	WT
CA-OCPHL-CA	United States	California	III	clade3 group3	2022-11-01	2022	Unknown	Unknown	Unknown	Axilla/Groin	SRR24806740	VF125AL	WT
CA-OCPHL-CA	United States	California	III	clade3 group3	2022-11-02	2022	Unknown	Unknown	Unknown	Axilla/Groin	SRR24806743	VF125AL	WT
CA-OCPHL-CA	United States	California	III	clade3 group3	2022-11-02	2022	Unknown	Unknown	Unknown	urine	SRR24806770	VF125AL	WT
CA-OCPHL-CA	United States	California	III	clade3 group3	2022-11-06	2022	Unknown	Unknown	Unknown	Tissue	SRR24806753	VF125AL	WT
CA-OCPHL-CA	United States	California	III	clade3 group3	2022-11-10	2022	Unknown	Unknown	Unknown	wound	SRR24806762	VF125AL	WT
CA-OCPHL-CA	United States	California	III	clade3 group3	2022-11-13	2022	Unknown	Unknown	Unknown	Axilla/Groin	SRR24806716	VF125AL	WT
CA-OCPHL-CA	United States	California	III	clade3 group3	2022-11-14	2022	Unknown	Unknown	Unknown	Axilla/Groin	SRR24806714	VF125AL	WT
CA-OCPHL-CA	United States	California	III	clade3 group3	2022-11-21	2022	Unknown	Unknown	Unknown	urine	SRR24806781	VF125AL	WT
CA-OCPHL-CA	United States	California	III	clade3 group3	2022-11-28	2022	Unknown	Unknown	Unknown	sputum	SRR24806773	VF125AL	WT
CA-OCPHL-CA	United States	California	III	clade3 group3	2022-12-04	2022	Unknown	Unknown	Unknown	Axilla/Groin	SRR24806738	VF125AL	WT
CA-OCPHL-CA	United States	California	III	clade3 group3	2022-12-10	2022	Unknown	Unknown	Unknown	blood	SRR24806674	VF125AL	WT
CA-OCPHL-CA	United States	California	III	clade3 group3	2022-12-12	2022	Unknown	Unknown	Unknown	blood	SRR24806776	VF125AL	WT
CA-OCPHL-CA	United States	California	III	clade3 group3	2022-12-18	2022	Unknown	Unknown	Unknown	Axilla/Groin	SRR24806741	VF125AL	WT
CA-OCPHL-CA	United States	California	III	clade3 group3	2022-12-18	2022	Unknown	Unknown	Unknown	Axilla/Groin	SRR24806731	VF125AL	WT
CA-OCPHL-CA	United States	California	III	clade3 group3	2022-12-30	2022	Unknown	Unknown	Unknown	urine	SRR24806675	VF125AL	WT
CA-OCPHL-CA	United States	California	III	clade3 group3	2023-01-01	2023	Unknown	Unknown	Unknown	Tissue	SRR24806713	VF125AL	WT
CA-OCPHL-CA	United States	California	III	clade3 group3	2023-01-04	2023	Unknown	Unknown	Unknown	Axilla/Groin	SRR24806745	VF125AL	WT
CA-OCPHL-CA	United States	California	III	clade3 group3	2023-01-04	2023	Unknown	Unknown	Unknown	Axilla/Groin	SRR24806734	VF125AL	WT
CA-OCPHL-CA	United States	California	III	clade3 group3	2023-01-04	2023	Unknown	Unknown	Unknown	Tissue	SRR24806723	VF125AL	WT
CA-OCPHL-CA	United States	California	III	clade3 group3	2023-01-17	2023	Unknown	Unknown	Unknown	Axilla/Groin	SRR24806733	VF125AL	WT
CA-OCPHL-CA	United States	California	III	clade3 group3	2023-01-22	2023	Unknown	Unknown	Unknown	urine	SRR24806778	VF125AL	WT
CA-OCPHL-CA	United States	California	III	clade3 group3	2023-01-30	2023	Unknown	Unknown	Unknown	urine	SRR24806672	VF125AL	WT
CA-OCPHL-CA	United States	California	III	clade3 group3	2023-02-10	2023	Unknown	Unknown	Unknown	wound	SRR24806779	VF125AL	WT
CA-OCPHL-CA	United States	California	III	clade3 group3	2023-02-17	2023	Unknown	Unknown	Unknown	urine	SRR24806780	VF125AL	WT
CA-OCPHL-CA	United States	California	III	clade3 group3	2023-02-21	2023	Unknown	Unknown	Unknown	urine	SRR24806769	VF125AL	WT
CA-OCPHL-CA	United States	California	III	clade3 group3	2023-03-16	2023	Unknown	Unknown	Unknown	wound	SRR24806775	VF125AL	WT
CT-WCPHL-CA	United States	Connecticut	III	clade3 group3	2020-09-08	2020	Unknown	Unknown	Unknown		SRR24621173	VF125AL	WT
B16820	United States	Florida	III	clade3 group3	2018	2018	Unknown	Unknown	Unknown	urine	SRR12073443	VF125AL	WT
B17050	United States	Florida	III	clade3 group3	2018	2018	Unknown	Unknown	Unknown	urine	SRR12073471	VF125AL	WT
B18531	United States	Florida	III	clade3 group3	2019	2019	Unknown	Unknown	Unknown	sputum	SRR12073436	VF125AL	WT
B18530	United States	Florida	III	clade3 group3	2019	2019	Unknown	Unknown	Unknown	aspirate	SRR12073437	VF125AL	WT
B18529	United States	Florida	III	clade3 group3	2019	2019	Unknown	Unknown	Unknown	blood	SRR12073438	VF125AL	WT
B18528	United States	Florida	III	clade3 group3	2019	2019	Unknown	Unknown	Unknown	blood	SRR12073439	VF125AL	WT
B18527	United States	Florida	III	clade3 group3	2019	2019	Unknown	Unknown	Unknown	sputum	SRR12073440	VF125AL	WT
B18526	United States	Florida	III	clade3 group3	2019	2019	Unknown	Unknown	Unknown	blood	SRR12073441	VF125AL	WT
B17981	United States	Florida	III	clade3 group3	2019	2019	Unknown	Unknown	Unknown	blood	SRR12073442	VF125AL	WT

B17980	United States	Florida	III	clade3 group3	2019	2019	Unknown	Unknown	Unknown	blood	SRR12073444	VF125AL	WT
B17979	United States	Florida	III	clade3 group3	2019	2019	Unknown	Unknown	Unknown	blood	SRR12073445	VF125AL	WT
B17915	United States	Florida	III	clade3 group3	2019	2019	Unknown	Unknown	Unknown	blood	SRR12073446	VF125AL	WT
B17914	United States	Florida	III	clade3 group3	2019	2019	Unknown	Unknown	Unknown	bronchial washings	SRR12073447	VF125AL	WT
B17913	United States	Florida	III	clade3 group3	2019	2019	Unknown	Unknown	Unknown	wound	SRR12073448	VF125AL	WT
B17912	United States	Florida	III	clade3 group3	2019	2019	Unknown	Unknown	Unknown	not applicable	SRR12073449	VF125AL	WT
B17911	United States	Florida	III	clade3 group3	2019	2019	Unknown	Unknown	Unknown	urine	SRR12073450	VF125AL	WT
B17910	United States	Florida	III	clade3 group3	2019	2019	Unknown	Unknown	Unknown	blood	SRR12073451	VF125AL	WT
B17909	United States	Florida	III	clade3 group3	2019	2019	Unknown	Unknown	Unknown	blood	SRR12073452	VF125AL	WT
B17908	United States	Florida	III	clade3 group3	2019	2019	Unknown	Unknown	Unknown	blood	SRR12073453	VF125AL	WT
B17905	United States	Florida	III	clade3 group3	2019	2019	Unknown	Unknown	Unknown	blood	SRR12073456	VF125AL	WT
B17904	United States	Florida	III	clade3 group3	2019	2019	Unknown	Unknown	Unknown	axilla and groin	SRR12073457	VF125AL	WT
B17798	United States	Florida	III	clade3 group3	2019	2019	Unknown	Unknown	Unknown	blood	SRR12073458	VF125AL	WT
B17656	United States	Florida	III	clade3 group3	2019	2019	Unknown	Unknown	Unknown	axilla and groin	SRR12073459	VF125AL	WT
B17655	United States	Florida	III	clade3 group3	2019	2019	Unknown	Unknown	Unknown	axilla and groin	SRR12073460	VF125AL	WT
B17654	United States	Florida	III	clade3 group3	2019	2019	Unknown	Unknown	Unknown	axilla and groin	SRR12073461	VF125AL	WT
B17653	United States	Florida	III	clade3 group3	2019	2019	Unknown	Unknown	Unknown	axilla and groin	SRR12073462	VF125AL	WT
B17651	United States	Florida	III	clade3 group3	2019	2019	Unknown	Unknown	Unknown	blood	SRR12073463	VF125AL	WT
B17054	United States	Florida	III	clade3 group3	2019	2019	Unknown	Unknown	Unknown	urine	SRR12073469	VF125AL	WT
B18759	United States	Florida	III	clade3 group3	2019	2019	Unknown	Unknown	Unknown	blood	SRR12073472	VF125AL	WT
B18754	United States	Florida	III	clade3 group3	2019	2019	Unknown	Unknown	Unknown	fluid body	SRR12073473	VF125AL	WT
B18734	United States	Florida	III	clade3 group3	2019	2019	Unknown	Unknown	Unknown	axilla and groin	SRR12073474	VF125AL	WT
B18733	United States	Florida	III	clade3 group3	2019	2019	Unknown	Unknown	Unknown	axilla and groin	SRR12073475	VF125AL	WT
B18732	United States	Florida	III	clade3 group3	2019	2019	Unknown	Unknown	Unknown	axilla and groin	SRR12073476	VF125AL	WT
B18669	United States	Florida	III	clade3 group3	2019	2019	Unknown	Unknown	Unknown	axilla and groin	SRR12073477	VF125AL	WT
B18667	United States	Florida	III	clade3 group3	2019	2019	Unknown	Unknown	Unknown	axilla and groin	SRR12073479	VF125AL	WT
B18666	United States	Florida	III	clade3 group3	2019	2019	Unknown	Unknown	Unknown	axilla and groin	SRR12073480	VF125AL	WT
B17018	United States	Florida	III	clade3 group3	2019	2019	Unknown	Unknown	Unknown	blood	SRR12073482	VF125AL	WT
B18534	United States	Florida	III	clade3 group3	2019	2019	Unknown	Unknown	Unknown	ulcer	SRR12073483	VF125AL	WT
B18906	United States	Florida	III	clade3 group3	2019	2019	Unknown	Unknown	Unknown	blood	SRR12526236	VF125AL	WT
B18908	United States	Florida	III	clade3 group3	2019	2019	Unknown	Unknown	Unknown	blood	SRR12526238	VF125AL	WT
B18925	United States	Florida	III	clade3 group3	2020	2020	Unknown	Unknown	Unknown	blood	SRR12526234	VF125AL	WT
B18891	United States	Florida	III	clade3 group3	2020	2020	Unknown	Unknown	Unknown	blood	SRR12526237	VF125AL	WT
B18830	United States	Florida	III	clade3 group3	2020	2020	Unknown	Unknown	Unknown	urine	SRR12526239	VF125AL	WT
B18919	United States	Florida	III	clade3 group3	2020	2020	Unknown	Unknown	Unknown	sputum	SRR12526240	VF125AL	WT
B18833	United States	Florida	III	clade3 group3	2020	2020	Unknown	Unknown	Unknown	wound	SRR12526241	VF125AL	WT
B18811	United States	Florida	III	clade3 group3	2020	2020	Unknown	Unknown	Unknown	urine	SRR12526242	VF125AL	WT
B18996	United States	Florida	III	clade3 group3	2020	2020	Unknown	Unknown	Unknown	blood	SRR12526243	VF125AL	WT
B18995	United States	Florida	III	clade3 group3	2020	2020	Unknown	Unknown	Unknown	wound	SRR12526244	VF125AL	WT
B18982	United States	Florida	III	clade3 group3	2020	2020	Unknown	Unknown	Unknown	blood	SRR12526245	VF125AL	WT
B18981	United States	Florida	III	clade3 group3	2020	2020	Unknown	Unknown	Unknown	bronchial washings	SRR12526246	VF125AL	WT
B18977	United States	Florida	III	clade3 group3	2020	2020	Unknown	Unknown	Unknown	blood	SRR12526248	VF125AL	WT
B18976	United States	Florida	III	clade3 group3	2020	2020	Unknown	Unknown	Unknown	urine	SRR12526249	VF125AL	WT
B18832	United States	Florida	III	clade3 group3	2020	2020	Unknown	Unknown	Unknown	bronchial washings	SRR12526251	VF125AL	WT
B18926	United States	Florida	III	clade3 group3	2020	2020	Unknown	Unknown	Unknown	blood	SRR12526252	VF125AL	WT
B19025	United States	Florida	III	clade3 group3	2020	2020	Unknown	Unknown	Unknown	urine	SRR12784119	VF125AL	WT
B19024	United States	Florida	III	clade3 group3	2020	2020	Unknown	Unknown	Unknown	blood	SRR12784120	VF125AL	WT
B18812	United States	Florida	III	clade3 group3	2020	2020	Unknown	Unknown	Unknown	aspirate	SRR12784121	VF125AL	WT
B19069	United States	Florida	III	clade3 group3	2020	2020	Unknown	Unknown	Unknown	sputum	SRR12784122	VF125AL	WT
B18933	United States	Florida	III	clade3 group3	2020	2020	Unknown	Unknown	Unknown	blood	SRR12784123	VF125AL	WT
B19007	United States	Florida	III	clade3 group3	2020	2020	Unknown	Unknown	Unknown	urine	SRR12784126	VF125AL	WT
B18997	United States	Florida	III	clade3 group3	2020	2020	Unknown	Unknown	Unknown	urine	SRR12784128	VF125AL	WT
B19132	United States	Florida	III	clade3 group3	2020	2020	Unknown	Unknown	Unknown	trachea aspirate	SRR12784129	VF125AL	WT
B19131	United States	Florida	III	clade3 group3	2020	2020	Unknown	Unknown	Unknown	blood	SRR12784131	VF125AL	WT
B19066	United States	Florida	III	clade3 group3	2020	2020	Unknown	Unknown	Unknown	aspirate	SRR12784133	VF125AL	WT
B19065	United States	Florida	III	clade3 group3	2020	2020	Unknown	Unknown	Unknown	blood	SRR12784134	VF125AL	WT
B19062	United States	Florida	III	clade3 group3	2020	2020	Unknown	Unknown	Unknown	wound	SRR12784135	VF125AL	WT
B19056	United States	Florida	III	clade3 group3	2020	2020	Unknown	Unknown	Unknown	urine	SRR12784136	VF125AL	WT
B19055	United States	Florida	III	clade3 group3	2020	2020	Unknown	Unknown	Unknown	blood	SRR12784137	VF125AL	WT
B19030	United States	Florida	III	clade3 group3	2020	2020	Unknown	Unknown	Unknown	urine	SRR12784138	VF125AL	WT
B19029	United States	Florida	III	clade3 group3	2020	2020	Unknown	Unknown	Unknown	urine	SRR12784139	VF125AL	WT
B19026	United States	Florida	III	clade3 group3	2020	2020	Unknown	Unknown	Unknown	sputum	SRR12784140	VF125AL	WT
B19006	United States	Florida	III	clade3 group3	2020	2020	Unknown	Unknown	Unknown	blood	SRR12784127	WT	WT
B18532	United States	Florida	III	clade3 group3	2020-07-07	2020	Unknown	Unknown	Unknown	Sputum	SRR12073435	VF125AL	WT
B18533	United States	Florida	III	clade3 group3	2020-07-07	2020	Unknown	Unknown	Unknown	Blood	SRR12073484	VF125AL	WT
B18665	United States	Florida	III	clade3 group3	2020-07-07	2020	Unknown	Unknown	Unknown	Axilla/Groin swab	SRR12073481	VF125AL	WT
B12631	United States	Indiana	III	clade3 group3	2017-03-16	2017	Resistant	Susceptible	Susceptible	Wound	SRR7909359	VF125AL	WT

DN474116W-A	United Kingdom	London	III	clade3 group3		2016-2018	Unknown	Unknown	Unknown	clinical	ERR2300769	VF125AL	WT
DN474116W-A	United Kingdom	London	III	clade3 group3		2016-2018	Unknown	Unknown	Unknown	clinical	ERR2300809	VF125AL	WT
DN474116W-B	United Kingdom	London	III	clade3 group3		2016-2018	Unknown	Unknown	Unknown	clinical	ERR2300770	VF125AL	WT
DN474116W-B	United Kingdom	London	III	clade3 group3		2016-2018	Unknown	Unknown	Unknown	clinical	ERR2300810	VF125AL	WT
DN474116W-C	United Kingdom	London	III	clade3 group3		2016-2018	Unknown	Unknown	Unknown	clinical	ERR2300771	VF125AL	WT
DN474116W-D	United Kingdom	London	III	clade3 group3		2016-2018	Unknown	Unknown	Unknown	clinical	ERR2300772	VF125AL	WT
DN474116W-D	United Kingdom	London	III	clade3 group3		2016-2018	Unknown	Unknown	Unknown	clinical	ERR2300796	VF125AL	WT
DN474116W-D	United Kingdom	London	III	clade3 group3		2016-2018	Unknown	Unknown	Unknown	clinical	ERR2300804	VF125AL	WT
DN474116W-E	United Kingdom	London	III	clade3 group3		2016-2018	Unknown	Unknown	Unknown	clinical	ERR2300789	VF125AL	WT
DN474116W-E	United Kingdom	London	III	clade3 group3		2016-2018	Unknown	Unknown	Unknown	clinical	ERR2300797	VF125AL	WT
DN474116W-F	United Kingdom	London	III	clade3 group3		2016-2018	Unknown	Unknown	Unknown	clinical	ERR2300798	VF125AL	WT
DN474116W-G	United Kingdom	London	III	clade3 group3		2016-2018	Unknown	Unknown	Unknown	clinical	ERR2300783	VF125AL	WT
NY-WCPHL-CA	United States	New York	III	clade3 group3	2019-10-11	2019	Unknown	Unknown	Unknown	Peritoneal dialysis catheter	SRR24621174	VF125AL	WT
NY-WCPHL-CA	United States	New York	III	clade3 group3	2020-09-25	2020	Unknown	Unknown	Unknown	wound	SRR24621161	VF125AL	WT
NY-WCPHL-CA	United States	New York	III	clade3 group3	2022-11	2022	Unknown	Unknown	Unknown	Blood (peripheral)	SRR24422751	VF125AL	WT
NY-WCPHL-CA	United States	New York	III	clade3 group3	2023-01	2022	Unknown	Unknown	Unknown	Wound finger	SRR24422757	VF125AL	WT
Patient-001	United Kingdom	Oxfordshire	III	clade3 group3	2015-02-02	2015	Unknown	Unknown	Unknown	clinical sample	SRR7976549	VF125AL	WT
Patient-002	United Kingdom	Oxfordshire	III	clade3 group3	2015-07-10	2015	Unknown	Unknown	Unknown	clinical sample	SRR7976579	VF125AL	WT
Patient-003	United Kingdom	Oxfordshire	III	clade3 group3	2015-07-13	2015	Unknown	Unknown	Unknown	clinical sample	SRR7976616	VF125AL	WT
Patient-004	United Kingdom	Oxfordshire	III	clade3 group3	2015-11-24	2015	Unknown	Unknown	Unknown	clinical sample	SRR7976582	VF125AL	WT
Patient-005	United Kingdom	Oxfordshire	III	clade3 group3	2016-01-02	2016	Unknown	Unknown	Unknown	clinical sample	SRR7976614	VF125AL	WT
Patient-006-01	United Kingdom	Oxfordshire	III	clade3 group3	2016-10-25	2016	Unknown	Unknown	Unknown	clinical sample	SRR7976557	VF125AL	WT
Patient-007	United Kingdom	Oxfordshire	III	clade3 group3	2016-11-17	2016	Unknown	Unknown	Unknown	clinical sample	SRR7976542	VF125AL	WT
Patient-008-01	United Kingdom	Oxfordshire	III	clade3 group3	2016-11-28	2016	Unknown	Unknown	Unknown	clinical sample	SRR7976584	VF125AL	WT
Patient-009-01	United Kingdom	Oxfordshire	III	clade3 group3	2016-12-05	2016	Unknown	Unknown	Unknown	clinical sample	SRR7976597	VF125AL	WT
Patient-010-01	United Kingdom	Oxfordshire	III	clade3 group3	2016-12-11	2016	Unknown	Unknown	Unknown	clinical sample	SRR7976591	VF125AL	WT
Patient-011-01	United Kingdom	Oxfordshire	III	clade3 group3	2016-12-19	2016	Unknown	Unknown	Unknown	clinical sample	SRR7976577	VF125AL	WT
Patient-012-01	United Kingdom	Oxfordshire	III	clade3 group3	2017-01-09	2017	Unknown	Unknown	Unknown	clinical sample	SRR7976604	VF125AL	WT
Patient-013-01	United Kingdom	Oxfordshire	III	clade3 group3	2017-01-10	2017	Unknown	Unknown	Unknown	clinical sample	SRR7976564	VF125AL	WT
Patient-015-01	United Kingdom	Oxfordshire	III	clade3 group3	2017-01-25	2017	Unknown	Unknown	Unknown	clinical sample	SRR7976599	VF125AL	WT
Patient-038	United Kingdom	Oxfordshire	III	clade3 group3	2017-01-25	2017	Unknown	Unknown	Unknown	clinical sample	SRR7976545	VF125AL	WT
Patient-016	United Kingdom	Oxfordshire	III	clade3 group3	2017-01-27	2017	Unknown	Unknown	Unknown	clinical sample	SRR7976578	VF125AL	WT
Patient-017	United Kingdom	Oxfordshire	III	clade3 group3	2017-02-10	2017	Unknown	Unknown	Unknown	clinical sample	SRR7976540	VF125AL	WT
Patient-019-01	United Kingdom	Oxfordshire	III	clade3 group3	2017-02-17	2017	Unknown	Unknown	Unknown	clinical sample	SRR7976552	VF125AL	WT
Patient-021-01	United Kingdom	Oxfordshire	III	clade3 group3	2017-03-08	2017	Unknown	Unknown	Unknown	clinical sample	SRR7976548	VF125AL	WT
Patient-022-01	United Kingdom	Oxfordshire	III	clade3 group3	2017-03-10	2017	Unknown	Unknown	Unknown	clinical sample	SRR7976572	VF125AL	WT
Patient-023	United Kingdom	Oxfordshire	III	clade3 group3	2017-03-18	2017	Unknown	Unknown	Unknown	clinical sample	SRR7976553	VF125AL	WT
Patient-024-01	United Kingdom	Oxfordshire	III	clade3 group3	2017-03-24	2017	Unknown	Unknown	Unknown	clinical sample	SRR7976592	VF125AL	WT
Patient-025-01	United Kingdom	Oxfordshire	III	clade3 group3	2017-03-27	2017	Unknown	Unknown	Unknown	clinical sample	SRR7976565	VF125AL	WT
Patient-026	United Kingdom	Oxfordshire	III	clade3 group3	2017-03-29	2017	Unknown	Unknown	Unknown	clinical sample	SRR7976600	VF125AL	WT
Patient-027-01	United Kingdom	Oxfordshire	III	clade3 group3	2017-03-31	2017	Unknown	Unknown	Unknown	clinical sample	SRR7976615	VF125AL	WT
Patient-028-01	United Kingdom	Oxfordshire	III	clade3 group3	2017-04-03	2017	Unknown	Unknown	Unknown	clinical sample	SRR7976585	VF125AL	WT
Hoist	United Kingdom	Oxfordshire	III	clade3 group3	2017-04-04	2017	Unknown	Unknown	Unknown	Environmental	SRR7976583	VF125AL	WT
TempProbe1	United Kingdom	Oxfordshire	III	clade3 group3	2017-04-04	2017	Unknown	Unknown	Unknown	Environmental	SRR7976593	VF125AL	WT
Patient-029-01	United Kingdom	Oxfordshire	III	clade3 group3	2017-04-07	2017	Unknown	Unknown	Unknown	clinical sample	SRR7976558	VF125AL	WT
Patient-030	United Kingdom	Oxfordshire	III	clade3 group3	2017-04-09	2017	Unknown	Unknown	Unknown	clinical sample	SRR7976598	VF125AL	WT
Patient-031	United Kingdom	Oxfordshire	III	clade3 group3	2017-04-10	2017	Unknown	Unknown	Unknown	clinical sample	SRR7976607	VF125AL	WT
Patient-037	United Kingdom	Oxfordshire	III	clade3 group3	2017-04-24	2017	Unknown	Unknown	Unknown	clinical sample	SRR7976603	VF125AL	WT
Patient-032	United Kingdom	Oxfordshire	III	clade3 group3	2017-04-26	2017	Unknown	Unknown	Unknown	clinical sample	SRR7976580	VF125AL	WT
Patient-033-01	United Kingdom	Oxfordshire	III	clade3 group3	2017-05-05	2017	Unknown	Unknown	Unknown	clinical sample	SRR7976541	VF125AL	WT
TempProbe2	United Kingdom	Oxfordshire	III	clade3 group3	2017-05-16	2017	Unknown	Unknown	Unknown	Environmental	SRR7976601	VF125AL	WT
TempProbe3	United Kingdom	Oxfordshire	III	clade3 group3	2017-05-16	2017	Unknown	Unknown	Unknown	Environmental	SRR7976602	VF125AL	WT
TempProbe4	United Kingdom	Oxfordshire	III	clade3 group3	2017-05-16	2017	Unknown	Unknown	Unknown	Environmental	SRR7976571	VF125AL	WT
TempProbe5	United Kingdom	Oxfordshire	III	clade3 group3	2017-05-16	2017	Unknown	Unknown	Unknown	Environmental	SRR7976560	VF125AL	WT
Patient-034	United Kingdom	Oxfordshire	III	clade3 group3	2017-05-22	2017	Unknown	Unknown	Unknown	clinical sample	SRR7976556	VF125AL	WT
RICU1-A1	China	Shenyang	III	clade3 group3	2016-04-06	2016	Resistant	Susceptible	Susceptible	Urine	SRR9316788	VF125AL	WT
RICU2-A2	China	Shenyang	III	clade3 group3	2016-06-03	2016	Resistant	Susceptible	Susceptible	Urine	SRR9316744	VF125AL	WT
RICU3-A3	China	Shenyang	III	clade3 group3	2016-06-17	2016	Resistant	Susceptible	Susceptible	Sputum	SRR9316754	VF125AL	WT
RICU4-A7	China	Shenyang	III	clade3 group3	2016-08-05	2016	Resistant	Susceptible	Susceptible	Sputum	SRR9316779	VF125AL	WT
RICU5-A12	China	Shenyang	III	clade3 group3	2016-12-07	2016	Resistant	Susceptible	Susceptible	Urinary catheter	SRR9316729	VF125AL	WT
NICU2-A13	China	Shenyang	III	clade3 group3	2016-12-24	2016	Resistant	Susceptible	Susceptible	Urine	SRR9316739	VF125AL	WT
RICU6-A14	China	Shenyang	III	clade3 group3	2017-01-26	2017	Resistant	Susceptible	Susceptible	Urinary catheter	SRR9316738	VF125AL	WT
NICU3-A15	China	Shenyang	III	clade3 group3	2017-02-04	2017	Resistant	Susceptible	Susceptible	Urine	SRR9316741	VF125AL	WT
NICU4-A19	China	Shenyang	III	clade3 group3	2017-03-17	2017	Resistant	Susceptible	Susceptible	Urine	SRR9316745	VF125AL	WT
RICU7-A22	China	Shenyang	III	clade3 group3	2017-07-29	2017	Resistant	Susceptible	Susceptible	Urine	SRR9316746	VF125AL	WT
NSICU1-A24	China	Shenyang	III	clade3 group3	2017-08-24	2017	Resistant	Susceptible	Susceptible	Urine	SRR9316751	VF125AL	WT
NICU5-A36	China	Shenyang	III	clade3 group3	2017-09-30	2017	Resistant	Susceptible	Susceptible	Urine	SRR9316756	VF125AL	WT

RICU8-A38	China	Shenyang	III	clade3 group3	2017-10-10	2017	Resistant	Susceptible	Susceptible	Urine	SRR9316765	VF125AL	WT
NICU6-A41	China	Shenyang	III	clade3 group3	2017-10-17	2017	Resistant	Susceptible	Susceptible	Fluid	SRR9316762	VF125AL	WT
NICU7-A42	China	Shenyang	III	clade3 group3	2017-11-22	2017	Resistant	Susceptible	Susceptible	Urine	SRR9316761	VF125AL	WT
NICU8-A43	China	Shenyang	III	clade3 group3	2017-11-24	2017	Resistant	Susceptible	Susceptible	Urine	SRR9316760	VF125AL	WT
NICU9-A50	China	Shenyang	III	clade3 group3	2017-12-12	2017	Resistant	Susceptible	Susceptible	Urine	SRR9316770	VF125AL	WT
RICU9-A52	China	Shenyang	III	clade3 group3	2017-12-31	2017	Resistant	Susceptible	Susceptible	Blood	SRR9316772	VF125AL	WT
RICU17-A108	China	Shenyang	III	clade3 group3	2016-12-14	2018	Resistant	Susceptible	Susceptible	Blood	SRR9316724	VF125AL	WT
RICU10-A57	China	Shenyang	III	clade3 group3	2018-01-24	2018	Resistant	Susceptible	Susceptible	Tracheal catheter	SRR9316775	VF125AL	WT
NICU10-A58	China	Shenyang	III	clade3 group3	2018-02-03	2018	Resistant	Susceptible	Susceptible	Urine	SRR9316776	VF125AL	WT
RICU11-A62	China	Shenyang	III	clade3 group3	2018-03-02	2018	Resistant	Susceptible	Susceptible	Sputum	SRR9316784	VF125AL	WT
C12-A109	China	Shenyang	III	clade3 group3	2018-03-09	2018	Resistant	Susceptible	Susceptible	Environmental	SRR9316725	VF125AL	WT
SICU1-A66	China	Shenyang	III	clade3 group3	2018-04-06	2018	Resistant	Susceptible	Susceptible	Urine	SRR9316780	VF125AL	WT
NICU11-A77	China	Shenyang	III	clade3 group3	2018-05-12	2018	Resistant	Susceptible	Susceptible	Blood	SRR9316803	VF125AL	WT
NICU12-A79	China	Shenyang	III	clade3 group3	2018-05-15	2018	Resistant	Susceptible	Susceptible	Urine	SRR9316800	VF125AL	WT
RICU12-A80	China	Shenyang	III	clade3 group3	2018-05-15	2018	Resistant	Susceptible	Susceptible	Sputum	SRR9316798	VF125AL	WT
RICU13-A83	China	Shenyang	III	clade3 group3	2018-05-31	2018	Resistant	Susceptible	Susceptible	Sputum	SRR9316810	VF125AL	WT
SICU2-A89	China	Shenyang	III	clade3 group3	2018-07-25	2018	Resistant	Susceptible	Susceptible	Sputum	SRR9316815	VF125AL	WT
NICU13-A93	China	Shenyang	III	clade3 group3	2018-08-10	2018	Resistant	Susceptible	Susceptible	Urine	SRR9316812	VF125AL	WT
RICU14-A95	China	Shenyang	III	clade3 group3	2018-08-23	2018	Resistant	Susceptible	Susceptible	Urinary catheter	SRR9316816	VF125AL	WT
NICU14-A96	China	Shenyang	III	clade3 group3	2018-08-27	2018	Resistant	Susceptible	Susceptible	Urine	SRR9316734	VF125AL	WT
NICU15-A100	China	Shenyang	III	clade3 group3	2018-09-04	2018	Resistant	Susceptible	Susceptible	Urine	SRR9316790	VF125AL	WT
NICU16-A101	China	Shenyang	III	clade3 group3	2018-09-04	2018	Resistant	Susceptible	Susceptible	Urine	SRR9316791	VF125AL	WT
RICU15-A105	China	Shenyang	III	clade3 group3	2018-09-30	2018	Resistant	Susceptible	Susceptible	Sputum	SRR9316795	VF125AL	WT
RICU16-A106	China	Shenyang	III	clade3 group3	2018-10-05	2018	Resistant	Susceptible	Susceptible	Sputum	SRR9316796	VF125AL	WT
RI-A112	China	Shenyang	III	clade3 group3	2018-11-07	2018	Resistant	Susceptible	Susceptible	Urine	SRR9316720	VF125AL	WT
RICU18-A113	China	Shenyang	III	clade3 group3	2018-11-13	2018	Resistant	Susceptible	Susceptible	Urinary catheter	SRR9316721	VF125AL	WT
RICU19-A115	China	Shenyang	III	clade3 group3	2018-11-20	2018	Resistant	Susceptible	Susceptible	Sputum	SRR9316723	VF125AL	WT
WM-18-176	Australia	Sydney	III	clade3 group3	2015	2015	Resistant	Susceptible	Susceptible	Sternal bone	SRR11485318	VF125AL	WT
WM-18-181	Australia	Sydney	III	clade3 group3	2018-09-05	2018	Resistant	Susceptible	Susceptible	Wound swab PICC site	SRR11485334	VF125AL	WT
WM-18-182	Australia	Sydney	III	clade3 group3	2018-09-05	2018	Resistant	Susceptible	Susceptible	sputum	SRR11485333	VF125AL	WT
WM-18-187	Australia	Sydney	III	clade3 group3	2018-09-06	2018	Resistant	Susceptible	Susceptible	Axilla swab	SRR11485324	VF125AL	WT
WM-18-188	Australia	Sydney	III	clade3 group3	2018-09-07	2018	Resistant	Susceptible	Susceptible	sputum	SRR11485323	VF125AL	WT
WM-18-189	Australia	Sydney	III	clade3 group3	2018-09-11	2018	Resistant	Susceptible	Susceptible	Axilla swab	SRR11485322	VF125AL	WT
WM-18-190	Australia	Sydney	III	clade3 group3	2018-09-11	2018	Resistant	Susceptible	Susceptible	Groin	SRR11485321	VF125AL	WT
WM-18-180	Australia	Sydney	III	clade3 group3	2018-09-20	2018	Resistant	Susceptible	Susceptible	nose swab	SRR11485320	VF125AL	WT
WM-18-197	Australia	Sydney	III	clade3 group3	2018-11-08	2018	Resistant	Susceptible	Susceptible	throat	SRR11485319	VF125AL	WT
B16425	Kenya	Unknown	III	clade3 group3	2011-11-08	2011	Unknown	Unknown	Unknown	blood	SRR10461245	VF125AL	WT
N18-02-486	Canada	Unknown	III	clade3 group1	2012	2012	Susceptible	Susceptible	Susceptible	Ear	SRR15192316	WT	WT
B11222	South Africa	Unknown	III	clade3 group3	2012-10-15	2012	Resistant	Susceptible	Susceptible	Blood	SRR3883454	VF125AL	WT
B12037	Canada	Unknown	III	clade3 group1	2012-10-18	2012	Susceptible	Susceptible	Susceptible	Ear fluid	SRR10461253	WT	WT
GCF 00277501	South Africa	Unknown	III	clade3 group3	2012-10-23	2012	Resistant	Susceptible	Susceptible	Blood		VF125AL	WT
B11223	South Africa	Unknown	III	clade3 group3	2013-01-15	2013	Resistant	Susceptible	Susceptible	Blood	SRR3883455	VF125AL	WT
B16431	Kenya	Unknown	III	clade3 group3	2013-10-10	2013	Resistant	Susceptible	Susceptible	Blood	SRR10461244	VF125AL	WT
B11224	South Africa	Unknown	III	clade3 group3	2013-10-12	2013	Resistant	Susceptible	Susceptible	Blood	SRR3883456	VF125AL	WT
B16441	Kenya	Unknown	III	clade3 group3	2013-10-22	2013	Resistant	Susceptible	Susceptible	Pleural	SRR10461238	VF125AL	WT
B16485	Kenya	Unknown	III	clade3 group3	2013-11-05	2013	Resistant	Susceptible	Susceptible	Blood	SRR10461219	VF125AL	WT
B16496	Kenya	Unknown	III	clade3 group3	2013-11-05	2013	Resistant	Susceptible	Susceptible	Blood	SRR10461215	VF125AL	WT
B16507	Kenya	Unknown	III	clade3 group3	2013-12-14	2013	Resistant	Susceptible	Susceptible	Blood	SRR10461212	VF125AL	WT
B16432	Kenya	Unknown	III	clade3 group3	2013-12-17	2013	Resistant	Susceptible	Susceptible	Blood	SRR10461211	VF125AL	WT
B16433	Kenya	Unknown	III	clade3 group3	2013-12-17	2013	Resistant	Susceptible	Susceptible	Blood	SRR10461243	VF125AL	WT
B16436	Kenya	Unknown	III	clade3 group3	2014-01-21	2014	Resistant	Susceptible	Susceptible	Blood	SRR10461242	VF125AL	WT
B16439	Kenya	Unknown	III	clade3 group3	2014-02-11	2014	Resistant	Susceptible	Susceptible	Blood	SRR10461240	VF125AL	WT
B16440	Kenya	Unknown	III	clade3 group3	2014-02-16	2014	Resistant	Susceptible	Resistant	Urine	SRR10461239	VF125AL	S639Y
B11226	South Africa	Unknown	III	clade3 group3	2014-03-09	2014	Resistant	Susceptible	Susceptible	Right leg	SRR3883458	VF125AL	WT
B11230	South Africa	Unknown	III	clade3 group2	2014-03-13	2014	Resistant	Susceptible	Susceptible	Urine	SRR3883463	WT	WT
B11227	South Africa	Unknown	III	clade3 group3	2014-03-16	2014	Resistant	Susceptible	Susceptible	Blood	SRR3883459	VF125AL	WT
B11228	South Africa	Unknown	III	clade3 group3	2014-03-18	2014	Resistant	Susceptible	Susceptible	Hip tissue	SRR3883461	VF125AL	WT
B16445	Kenya	Unknown	III	clade3 group3	2014-03-21	2014	Resistant	Susceptible	Susceptible	Blood	SRR10461236	VF125AL	WT
B16451	Kenya	Unknown	III	clade3 group3	2014-04-07	2014	Resistant	Susceptible	Susceptible	Blood	SRR10461234	VF125AL	WT
B16454	Kenya	Unknown	III	clade3 group3	2014-04-15	2014	Resistant	Susceptible	Susceptible	Vascular tip	SRR10461233	VF125AL	WT
B11225	South Africa	Unknown	III	clade3 group3	2014-04-20	2014	Resistant	Susceptible	Susceptible	Urine	SRR3883457	VF125AL	WT
B11229	South Africa	Unknown	III	clade3 group3	2014-04-21	2014	Resistant	Susceptible	Susceptible	Urine (catheter)	SRR3883462	VF125AL	WT
B16457	Kenya	Unknown	III	clade3 group3	2014-05-22	2014	Resistant	Susceptible	Susceptible	Blood	SRR10461232	VF125AL	WT
B16459	Kenya	Unknown	III	clade3 group3	2014-08-16	2014	Resistant	Susceptible	Susceptible	Blood	SRR10461231	VF125AL	WT
B16461	Kenya	Unknown	III	clade3 group3	2014-10-08	2014	Resistant	Susceptible	Susceptible	Blood	SRR10461230	VF125AL	WT
B16466	Kenya	Unknown	III	clade3 group3	2015-01-21	2015	Resistant	Susceptible	Susceptible	Blood	SRR10461229	VF125AL	WT
B16467	Kenya	Unknown	III	clade3 group3	2015-02-02	2015	Resistant	Susceptible	Susceptible	Blood	SRR10461228	VF125AL	WT

B16469	Kenya	Unknown	III	clade3 group3	2015-02-09	2015	Resistant	Susceptible	Susceptible	Blood	SRR10461227	VF125AL	WT
CA S97	Australia	Unknown	III	clade3 group3	2015-07-15	2015	Resistant	Susceptible	Susceptible	Bone	SRR7657927	VF125AL	WT
B16473	Kenya	Unknown	III	clade3 group3	2015-07-27	2015	Resistant	Susceptible	Susceptible	Pleural	SRR10461225	VF125AL	WT
B16481	Kenya	Unknown	III	clade3 group3	2016-02-27	2016	Resistant	Susceptible	Susceptible	Blood	SRR10461222	VF125AL	WT
B16482	Kenya	Unknown	III	clade3 group3	2016-03-02	2016	Resistant	Susceptible	Susceptible	Blood	SRR10461221	VF125AL	WT
B16484	Kenya	Unknown	III	clade3 group3	2016-03-21	2016	Resistant	Susceptible	Resistant	Urine	SRR10461220	VF125AL	WT
MRL 3560	South Africa	Unknown	III	clade3 group3	2017	2017	Unknown	Unknown	Unknown	Blood	SRR14802928	VF125AL	S639P
MRL 3561	South Africa	Unknown	III	clade3 group3	2017	2017	Unknown	Unknown	Unknown	Blood	SRR14802927	VF125AL	S639P
MRL 3562	South Africa	Unknown	III	clade3 group3	2017	2017	Unknown	Unknown	Unknown	Blood	SRR14802926	VF125AL	S639P
MRL 2491	South Africa	Unknown	III	clade3 group3	2017	2017	Unknown	Unknown	Unknown	Blood	SRR14802846	VF125AL	WT
MRL 2526	South Africa	Unknown	III	clade3 group3	2017	2017	Unknown	Unknown	Unknown	Blood	SRR14802845	VF125AL	WT
MRL 2546	South Africa	Unknown	III	clade3 group3	2017	2017	Unknown	Unknown	Unknown	Blood	SRR14802843	VF125AL	WT
MRL 2547	South Africa	Unknown	III	clade3 group3	2017	2017	Unknown	Unknown	Unknown	Blood	SRR14802842	VF125AL	WT
MRL 2566	South Africa	Unknown	III	clade3 group3	2017	2017	Unknown	Unknown	Unknown	Blood	SRR14802841	VF125AL	WT
MRL 2609	South Africa	Unknown	III	clade3 group3	2017	2017	Unknown	Unknown	Unknown	Blood	SRR14802840	VF125AL	WT
MRL 2634	South Africa	Unknown	III	clade3 group3	2017	2017	Unknown	Unknown	Unknown	Blood	SRR14802839	VF125AL	WT
MRL 2678	South Africa	Unknown	III	clade3 group3	2017	2017	Unknown	Unknown	Unknown	Blood	SRR14802838	VF125AL	WT
MRL 2736	South Africa	Unknown	III	clade3 group3	2017	2017	Unknown	Unknown	Unknown	Blood	SRR14802836	VF125AL	WT
MRL 2859	South Africa	Unknown	III	clade3 group3	2017	2017	Unknown	Unknown	Unknown	Blood	SRR14802835	VF125AL	WT
MRL 2980	South Africa	Unknown	III	clade3 group3	2017	2017	Unknown	Unknown	Unknown	Blood	SRR14802834	VF125AL	WT
MRL 3225	South Africa	Unknown	III	clade3 group3	2017	2017	Unknown	Unknown	Unknown	Blood	SRR14802832	VF125AL	WT
MRL 3269	South Africa	Unknown	III	clade3 group3	2017	2017	Unknown	Unknown	Unknown	Blood	SRR14802940	VF125AL	WT
MRL 3289	South Africa	Unknown	III	clade3 group3	2017	2017	Unknown	Unknown	Unknown	Blood	SRR14802938	VF125AL	WT
MRL 3338	South Africa	Unknown	III	clade3 group3	2017	2017	Unknown	Unknown	Unknown	Blood	SRR14802937	VF125AL	WT
MRL 3345	South Africa	Unknown	III	clade3 group3	2017	2017	Unknown	Unknown	Unknown	Blood	SRR14802936	VF125AL	WT
MRL 3402	South Africa	Unknown	III	clade3 group3	2017	2017	Unknown	Unknown	Unknown	Blood	SRR14802935	VF125AL	WT
MRL 3405	South Africa	Unknown	III	clade3 group3	2017	2017	Unknown	Unknown	Unknown	Blood	SRR14802934	VF125AL	WT
MRL 3406	South Africa	Unknown	III	clade3 group3	2017	2017	Unknown	Unknown	Unknown	Blood	SRR14802933	VF125AL	WT
MRL 3411	South Africa	Unknown	III	clade3 group3	2017	2017	Unknown	Unknown	Unknown	Blood	SRR14802932	VF125AL	WT
MRL 3511	South Africa	Unknown	III	clade3 group3	2017	2017	Unknown	Unknown	Unknown	Blood	SRR14802931	VF125AL	WT
MRL 3589	South Africa	Unknown	III	clade3 group3	2017	2017	Unknown	Unknown	Unknown	Blood	SRR14802925	VF125AL	WT
MRL 3706	South Africa	Unknown	III	clade3 group3	2017	2017	Unknown	Unknown	Unknown	Blood	SRR14802924	VF125AL	WT
MRL 3770	South Africa	Unknown	III	clade3 group3	2017	2017	Unknown	Unknown	Unknown	Blood	SRR14802922	VF125AL	WT
MRL 3788	South Africa	Unknown	III	clade3 group3	2017	2017	Unknown	Unknown	Unknown	Blood	SRR14802921	VF125AL	WT
MRL 4152	South Africa	Unknown	III	clade3 group3	2017	2017	Unknown	Unknown	Unknown	Blood	SRR14802920	VF125AL	WT
MRL 4414	South Africa	Unknown	III	clade3 group3	2017	2017	Unknown	Unknown	Unknown	Blood	SRR14802918	VF125AL	WT
MRL 4603	South Africa	Unknown	III	clade3 group3	2017	2017	Unknown	Unknown	Unknown	Blood	SRR14802916	VF125AL	WT
MRL 4642	South Africa	Unknown	III	clade3 group3	2017	2017	Unknown	Unknown	Unknown	Blood	SRR14802915	VF125AL	WT
MRL 4742	South Africa	Unknown	III	clade3 group3	2017	2017	Unknown	Unknown	Unknown	Blood	SRR14802914	VF125AL	WT
MRL 4836	South Africa	Unknown	III	clade3 group3	2017	2017	Unknown	Unknown	Unknown	Blood	SRR14802913	VF125AL	WT
MRL 4845	South Africa	Unknown	III	clade3 group3	2017	2017	Unknown	Unknown	Unknown	Blood	SRR14802912	VF125AL	WT
MRL 4888	South Africa	Unknown	III	clade3 group3	2017	2017	Unknown	Unknown	Unknown	Blood	SRR14802911	VF125AL	WT
MRL 4895	South Africa	Unknown	III	clade3 group3	2017	2017	Unknown	Unknown	Unknown	Blood	SRR14802910	VF125AL	WT
MRL 4925	South Africa	Unknown	III	clade3 group3	2017	2017	Unknown	Unknown	Unknown	Blood	SRR14802907	VF125AL	WT
MRL 4926	South Africa	Unknown	III	clade3 group3	2017	2017	Unknown	Unknown	Unknown	Blood	SRR14802906	VF125AL	WT
MRL 4928	South Africa	Unknown	III	clade3 group3	2017	2017	Unknown	Unknown	Unknown	Blood	SRR14802905	VF125AL	WT
MRL 4930	South Africa	Unknown	III	clade3 group3	2017	2017	Unknown	Unknown	Unknown	Blood	SRR14802904	VF125AL	WT
MRL 4943	South Africa	Unknown	III	clade3 group3	2017	2017	Unknown	Unknown	Unknown	Blood	SRR14802902	VF125AL	WT
MRL 4944	South Africa	Unknown	III	clade3 group3	2017	2017	Unknown	Unknown	Unknown	Blood	SRR14802901	VF125AL	WT
MRL 5006	South Africa	Unknown	III	clade3 group3	2017	2017	Unknown	Unknown	Unknown	Blood	SRR14802900	VF125AL	WT
MRL 5109	South Africa	Unknown	III	clade3 group3	2017	2017	Unknown	Unknown	Unknown	Blood	SRR14802899	VF125AL	WT
MRL 5222	South Africa	Unknown	III	clade3 group3	2017	2017	Unknown	Unknown	Unknown	Blood	SRR14802895	VF125AL	WT
MRL 5268	South Africa	Unknown	III	clade3 group3	2017	2017	Unknown	Unknown	Unknown	Blood	SRR14802893	VF125AL	WT
MRL 5305	South Africa	Unknown	III	clade3 group3	2017	2017	Unknown	Unknown	Unknown	Blood	SRR14802892	VF125AL	WT
MRL 5386	South Africa	Unknown	III	clade3 group3	2017	2017	Unknown	Unknown	Unknown	Blood	SRR14802891	VF125AL	WT
MRL 5405	South Africa	Unknown	III	clade3 group3	2017	2017	Unknown	Unknown	Unknown	Blood	SRR14802890	VF125AL	WT
MRL 5483	South Africa	Unknown	III	clade3 group3	2017	2017	Unknown	Unknown	Unknown	Blood	SRR14802889	VF125AL	WT
MRL 5543	South Africa	Unknown	III	clade3 group3	2017	2017	Unknown	Unknown	Unknown	Blood	SRR14802888	VF125AL	WT
MRL 5547	South Africa	Unknown	III	clade3 group3	2017	2017	Unknown	Unknown	Unknown	Blood	SRR14802887	VF125AL	WT
MRL 5561	South Africa	Unknown	III	clade3 group3	2017	2017	Unknown	Unknown	Unknown	Blood	SRR14802885	VF125AL	WT
MRL 5574	South Africa	Unknown	III	clade3 group3	2017	2017	Unknown	Unknown	Unknown	Blood	SRR14802884	VF125AL	WT
MRL 5580	South Africa	Unknown	III	clade3 group3	2017	2017	Unknown	Unknown	Unknown	Blood	SRR14802883	VF125AL	WT
MRL 5585	South Africa	Unknown	III	clade3 group3	2017	2017	Unknown	Unknown	Unknown	Blood	SRR14802882	VF125AL	WT
MRL 5590	South Africa	Unknown	III	clade3 group3	2017	2017	Unknown	Unknown	Unknown	Blood	SRR14802897	VF125AL	WT
MRL 5591	South Africa	Unknown	III	clade3 group3	2017	2017	Unknown	Unknown	Unknown	Blood	SRR14802886	VF125AL	WT
MRL 5593	South Africa	Unknown	III	clade3 group3	2017	2017	Unknown	Unknown	Unknown	Blood	SRR14802881	VF125AL	WT
MRL 5624	South Africa	Unknown	III	clade3 group3	2017	2017	Unknown	Unknown	Unknown	Blood	SRR14802880	VF125AL	WT

MRL 5625	South Africa	Unknown	III	clade3 group3	2017	2017	Unknown	Unknown	Unknown	Blood	SRR14802879	VF125AL	WT
MRL 5704	South Africa	Unknown	III	clade3 group3	2017	2017	Unknown	Unknown	Unknown	Blood	SRR14802878	VF125AL	WT
MRL 5714	South Africa	Unknown	III	clade3 group3	2017	2017	Unknown	Unknown	Unknown	Blood	SRR14802875	VF125AL	WT
MRL 5715	South Africa	Unknown	III	clade3 group3	2017	2017	Unknown	Unknown	Unknown	Blood	SRR14802944	VF125AL	WT
MRL 5716	South Africa	Unknown	III	clade3 group3	2017	2017	Unknown	Unknown	Unknown	Blood	SRR14802858	VF125AL	WT
MRL 5734	South Africa	Unknown	III	clade3 group3	2017	2017	Unknown	Unknown	Unknown	Blood	SRR14802877	VF125AL	WT
MRL 5762	South Africa	Unknown	III	clade3 group3	2017	2017	Unknown	Unknown	Unknown	Blood	SRR14802852	VF125AL	WT
MRL 5771	South Africa	Unknown	III	clade3 group3	2017	2017	Unknown	Unknown	Unknown	Blood	SRR14802876	VF125AL	WT
MRL 5946	South Africa	Unknown	III	clade3 group3	2017	2017	Unknown	Unknown	Unknown	Blood	SRR14802874	VF125AL	WT
MRL 5948	South Africa	Unknown	III	clade3 group3	2017	2017	Unknown	Unknown	Unknown	Blood	SRR14802850	VF125AL	WT
MRL 5953	South Africa	Unknown	III	clade3 group3	2017	2017	Unknown	Unknown	Unknown	Blood	SRR14802873	VF125AL	WT
MRL 6057	South Africa	Unknown	III	clade3 group3	2017	2017	Unknown	Unknown	Unknown	Blood	SRR14802849	VF125AL	WT
MRL 6241	South Africa	Unknown	III	clade3 group3	2017	2017	Unknown	Unknown	Unknown	Blood	SRR14802872	VF125AL	WT
MRL 6277b	South Africa	Unknown	III	clade3 group3	2017	2017	Unknown	Unknown	Unknown	Blood	SRR14802871	VF125AL	WT
MRL B159-2	South Africa	Unknown	III	clade3 group3	2017	2017	Unknown	Unknown	Unknown	Environmental	SRR14802870	VF125AL	WT
MRL B195-1	South Africa	Unknown	III	clade3 group3	2017	2017	Unknown	Unknown	Unknown	Environmental	SRR14802869	VF125AL	WT
MRL B235-1	South Africa	Unknown	III	clade3 group3	2017	2017	Unknown	Unknown	Unknown	Environmental	SRR14802867	VF125AL	WT
MRL B296-3	South Africa	Unknown	III	clade3 group3	2017	2017	Unknown	Unknown	Unknown	Environmental	SRR14802942	VF125AL	WT
MRL S158-1	South Africa	Unknown	III	clade3 group3	2017	2017	Unknown	Unknown	Unknown	Environmental	SRR14802939	VF125AL	WT
MRL S16	South Africa	Unknown	III	clade3 group3	2017	2017	Unknown	Unknown	Unknown	Axilla/Groin swab	SRR14802943	VF125AL	WT
MRL S19	South Africa	Unknown	III	clade3 group3	2017	2017	Unknown	Unknown	Unknown	Axilla/Groin swab	SRR14802868	VF125AL	WT
MRL S2	South Africa	Unknown	III	clade3 group3	2017	2017	Unknown	Unknown	Unknown	Axilla/Groin swab	SRR14802866	VF125AL	WT
MRL S200-1	South Africa	Unknown	III	clade3 group3	2017	2017	Unknown	Unknown	Unknown	Environmental	SRR14802865	VF125AL	WT
MRL S26	South Africa	Unknown	III	clade3 group3	2017	2017	Unknown	Unknown	Unknown	Axilla/Groin swab	SRR14802864	VF125AL	WT
MRL S27	South Africa	Unknown	III	clade3 group3	2017	2017	Unknown	Unknown	Unknown	Axilla/Groin swab	SRR14802863	VF125AL	WT
MRL S29	South Africa	Unknown	III	clade3 group3	2017	2017	Unknown	Unknown	Unknown	Axilla/Groin swab	SRR14802862	VF125AL	WT
MRL S31	South Africa	Unknown	III	clade3 group3	2017	2017	Unknown	Unknown	Unknown	Axilla/Groin swab	SRR14802861	VF125AL	WT
MRL S316-2	South Africa	Unknown	III	clade3 group3	2017	2017	Unknown	Unknown	Unknown	Environmental	SRR14802860	VF125AL	WT
MRL S33	South Africa	Unknown	III	clade3 group3	2017	2017	Unknown	Unknown	Unknown	Axilla/Groin swab	SRR14802859	VF125AL	WT
MRL S353-2	South Africa	Unknown	III	clade3 group3	2017	2017	Unknown	Unknown	Unknown	Environmental	SRR14802857	VF125AL	WT
MRL S360-1	South Africa	Unknown	III	clade3 group3	2017	2017	Unknown	Unknown	Unknown	Environmental	SRR14802856	VF125AL	WT
MRL S4	South Africa	Unknown	III	clade3 group3	2017	2017	Unknown	Unknown	Unknown	Axilla/Groin swab	SRR14802855	VF125AL	WT
MRL S6	South Africa	Unknown	III	clade3 group3	2017	2017	Unknown	Unknown	Unknown	Axilla/Groin swab	SRR14802854	VF125AL	WT
MRL W-KNH04	South Africa	Unknown	III	clade3 group3	2017	2017	Unknown	Unknown	Unknown	Environmental	SRR14802853	VF125AL	WT
WM-18-178	South Africa	Unknown	III	clade3 group3	2017	2017	Resistant	Susceptible	Susceptible	Blood culture	SRR11485332	VF125AL	WT
WM-18-179	South Africa	Unknown	III	clade3 group3	2017	2017	Resistant	Susceptible	Susceptible	Blood culture	SRR11485331	VF125AL	WT
MRL 3758	South Africa	Unknown	III	clade3 group2	2017	2017	Unknown	Unknown	Unknown	Blood	SRR14802923	WT	WT
B16514	Kenya	Unknown	III	clade3 group3	2017-07-05	2017	Resistant	Susceptible	Susceptible	Blood	SRR10461207	VF125AL	WT
B16491	Kenya	Unknown	III	clade3 group3	2017-10-02	2017	Resistant	Susceptible	Susceptible	Urine	SRR10461216	VF125AL	F635dup
B16404	Kenya	Unknown	III	clade3 group3	2017-10-16	2017	Resistant	Susceptible	Resistant	Urine	SRR10461146	VF125AL	F635dup
B16504	Kenya	Unknown	III	clade3 group3	2017-10-23	2017	Resistant	Susceptible	Susceptible	Urine	SRR10461213	VF125AL	WT
B16406	Kenya	Unknown	III	clade3 group3	2017-10-30	2017	Resistant	Susceptible	Resistant	Urine	SRR10461145	VF125AL	S639F
B16519	Kenya	Unknown	III	clade3 group3	2018-03-07	2018	Resistant	Susceptible	Susceptible	Blood	SRR10461205	VF125AL	WT
B16419	Kenya	Unknown	III	clade3 group3	2018-04-30	2018	Resistant	Susceptible	Susceptible	Blood	SRR10461141	VF125AL	WT
B16424	Kenya	Unknown	III	clade3 group3	2018-05-16	2018	Resistant	Susceptible	Susceptible	Blood	SRR10461138	VF125AL	WT
B16410	Kenya	Unknown	III	clade3 group3	2018-06-15	2018	Resistant	Susceptible	Susceptible	Blood	SRR10461144	VF125AL	WT
B16417	Kenya	Unknown	III	clade3 group3	2018-06-26	2018	Resistant	Susceptible	Susceptible	Blood	SRR10461142	VF125AL	WT
WM-18-177	South Africa	Unknown	III	clade3 group3	2018-07	2018	Resistant	Susceptible	Susceptible	Blood culture	SRR11485317	VF125AL	WT
B16415	Kenya	Unknown	III	clade3 group3	2018-07-16	2018	Resistant	Susceptible	Susceptible	Blood	SRR10461143	VF125AL	WT
N19-01-047	Canada	Unknown	III	clade3 group3	2019	2019	Resistant	Susceptible	Susceptible	Bronchial wash	SRR15192306	VF125AL	WT
B17835	United States	Unknown	III	clade3 group3	2019-04-22	2019	Unknown	Unknown	Unknown	nose	SRR14252432	VF125AL	WT
B18683	Kenya	Unknown	III	clade3 group3	2019-11-25	2019	Unknown	Unknown	Unknown	skin swab	SRR14252431	VF125AL	WT
B19285	United States	Unknown	III	clade3 group3	2020-09-03	2020	Unknown	Unknown	Unknown	urine	SRR14590379	VF125AL	WT
B19448	United States	Unknown	III	clade3 group3	2020-10-02	2020	Unknown	Unknown	Unknown	leg	SRR14590390	VF125AL	WT
B19584	United States	Unknown	III	clade3 group3	2020-10-29	2020	Unknown	Unknown	Unknown	not applicable	SRR14590383	VF125AL	WT
B19913	United States	Unknown	III	clade3 group3	2020-11-16	2020	Unknown	Unknown	Unknown	blood	SRR14590389	VF125AL	WT
B19920	United States	Unknown	III	clade3 group3	2021-01-04	2021	Unknown	Unknown	Unknown	urine	SRR14590388	VF125AL	WT
B19985	United States	Unknown	III	clade3 group3	2021-01-15	2021	Unknown	Unknown	Unknown	wound	SRR14590385	VF125AL	WT
B19983	United States	Unknown	III	clade3 group3	2021-01-19	2021	Unknown	Unknown	Unknown	wound	SRR14590386	VF125AL	WT
B19944	United States	Unknown	III	clade3 group3	2021-01-24	2021	Unknown	Unknown	Unknown	sputum	SRR14590384	VF125AL	WT
Cau4	Austria	Unknown	III	clade3 group3	2021-10	2021	Resistant	Susceptible	Susceptible	Throat	SRR23080876	VF125AL	WT
AA-194	Spain	Valencia	III	clade3 group3	2016-06-28	2016	Resistant	Susceptible	Susceptible	Blood	SRR10461267	VF125AL	WT
AA-200	Spain	Valencia	III	clade3 group3	2016-08-28	2016	Resistant	Susceptible	Susceptible	Blood	SRR10461266	VF125AL	WT
AA-214	Spain	Valencia	III	clade3 group3	2016-11-15	2016	Resistant	Susceptible	Susceptible	Blood	SRR10461265	VF125AL	WT
S1	Spain	Valencia	III	clade3 group3	2017-09-03	2017	Resistant	Unknown	Susceptible	Urinary catheter	ERR12321083	VF125AL	WT
S2	Spain	Valencia	III	clade3 group3	2017-10-14	2017	Resistant	Unknown	Susceptible	Blood (catheter)	ERR12321099	VF125AL	WT
S4	Spain	Valencia	III	clade3 group3	2017-10-18	2017	Resistant	Unknown	Susceptible	Blood (venipuncture)	ERR12321102	VF125AL	WT

S8	Spain	Valencia	III	clade3 group3	2017-12-12	2017	Resistant	Unknown	Susceptible	Blood (catheter)	ERR12321106	VF125AL	WT
S14	Spain	Valencia	III	clade3 group3	2018-02-06	2018	Resistant	Unknown	Susceptible	Blood (venipuncture)	ERR12321082	VF125AL	WT
S28	Spain	Valencia	III	clade3 group3	2018-04-30	2018	Resistant	Unknown	Susceptible	Blood (catheter)	ERR12321090	VF125AL	WT
S33	Spain	Valencia	III	clade3 group3	2018-05-27	2018	Resistant	Unknown	Susceptible	Blood (catheter)	ERR12321092	VF125AL	WT
S35	Spain	Valencia	III	clade3 group3	2018-06-28	2018	Resistant	Unknown	Susceptible	Blood (venipuncture)	ERR12321075	VF125AL	WT
S44	Spain	Valencia	III	clade3 group3	2018-08-31	2018	Resistant	Unknown	Susceptible	Catheter	ERR12321096	VF125AL	WT
S56	Spain	Valencia	III	clade3 group3	2018-11-13	2018	Resistant	Unknown	Susceptible	Lumbar puncture	ERR12321097	VF125AL	WT
S62	Spain	Valencia	III	clade3 group3	2019-01-23	2019	Resistant	Unknown	Susceptible	Catheter	ERR12321081	VF125AL	WT
S67	Spain	Valencia	III	clade3 group3	2019-02-22	2019	Resistant	Unknown	Resistant	Blood (catheter)	ERR12321078	VF125AL	S639Y
S72	Spain	Valencia	III	clade3 group3	2019-04-02	2019	Resistant	Unknown	Susceptible	Urinary catheter	ERR12321076	VF125AL	WT
S75	Spain	Valencia	III	clade3 group3	2019-05-21	2019	Resistant	Unknown	Susceptible	Blood (catheter)	ERR12321103	VF125AL	WT
S77	Spain	Valencia	III	clade3 group3	2019-06-02	2019	Resistant	Unknown	Susceptible	Urine	ERR12321087	VF125AL	WT
S81	Spain	Valencia	III	clade3 group3	2019-08-16	2019	Resistant	Unknown	Susceptible	Blood (catheter)	ERR12321085	VF125AL	WT
S82	Spain	Valencia	III	clade3 group3	2019-09-05	2019	Resistant	Unknown	Susceptible	Catheter tip	ERR12321095	VF125AL	WT
S86	Spain	Valencia	III	clade3 group3	2019-10-18	2019	Resistant	Unknown	Susceptible	Blood (catheter)	ERR12321084	VF125AL	WT
S94	Spain	Valencia	III	clade3 group3	2019-12-05	2019	Resistant	Unknown	Susceptible	Blood (catheter)	ERR12321080	VF125AL	WT
S99	Spain	Valencia	III	clade3 group3	2020-01-29	2020	Resistant	Unknown	Susceptible	Blood (venipuncture)	ERR12321074	VF125AL	WT
S103	Spain	Valencia	III	clade3 group3	2020-02-04	2020	Resistant	Unknown	Susceptible	Urinary catheter	ERR12321104	VF125AL	WT
S117	Spain	Valencia	III	clade3 group3	2020-03-24	2020	Resistant	Unknown	Susceptible	Blood (venipuncture)	ERR12321086	VF125AL	WT
S127	Spain	Valencia	III	clade3 group3	2020-04-14	2020	Resistant	Unknown	Susceptible	Axillary-rectal exudate	ERR12321098	VF125AL	WT
S129	Spain	Valencia	III	clade3 group3	2020-05-04	2020	Resistant	Unknown	Susceptible	Blood (catheter)	ERR12321077	VF125AL	WT
S131	Spain	Valencia	III	clade3 group3	2020-06-09	2020	Resistant	Unknown	Susceptible	Axillary-rectal exudate	ERR12321100	VF125AL	WT
S132	Spain	Valencia	III	clade3 group3	2020-07-07	2020	Resistant	Unknown	Susceptible	Blood (catheter)	ERR12321108	VF125AL	WT
S134	Spain	Valencia	III	clade3 group3	2020-08-17	2020	Resistant	Unknown	Susceptible	Axillary-rectal exudate	ERR12321088	VF125AL	WT
S135	Spain	Valencia	III	clade3 group3	2020-09-09	2020	Resistant	Unknown	Susceptible	Blood (catheter)	ERR12321091	VF125AL	WT
S138	Spain	Valencia	III	clade3 group3	2020-11-30	2020	Resistant	Unknown	Susceptible	Blood (catheter)	ERR12321105	VF125AL	WT
S140	Spain	Valencia	III	clade3 group3	2020-12-18	2020	Resistant	Unknown	Susceptible	Blood (catheter)	ERR12321101	VF125AL	WT
S141	Spain	Valencia	III	clade3 group3	2021-01-12	2021	Resistant	Unknown	Susceptible	Axillary-rectal exudate	ERR12321094	VF125AL	WT
S145	Spain	Valencia	III	clade3 group3	2021-02-11	2021	Resistant	Unknown	Susceptible	Blood (catheter)	ERR12321107	VF125AL	WT
S147	Spain	Valencia	III	clade3 group3	2021-04-17	2021	Resistant	Unknown	Susceptible	Blood (catheter)	ERR12321089	VF125AL	WT
S150	Spain	Valencia	III	clade3 group3	2021-05-12	2021	Resistant	Unknown	Susceptible	Blood (venipuncture)	ERR12321093	VF125AL	WT
S151	Spain	Valencia	III	clade3 group3	2021-06-14	2021	Resistant	Unknown	Susceptible	Environmental	ERR12321079	VF125AL	WT

Supplementary Table 4. Metadata (sample origin, phenotypic and genotypic characteristics of resistance-related mutations) of all *Candida auris* clade III genomes used for dating analysis (n=380).

ID	Specimen_orig	Collection_dat	Collection_y	Phylogenetic clad	clade3_group	Country	City/State	Outbreak/Cluste	fluconazole (FLC	Amphotericin B (AMB	Micafungin (MFG	Specimen_source	SRA_run	Study	ERG11 (FLC)	FKS1 (MFG)
AA-194	Clinical	2016-06-28	2016	III	clade3_group3	Spain	Valencia	Spain	Resistant	Susceptible	Susceptible	Blood	SRR10461267	Chow 2020	VF125AL	WT
AA-200	Clinical	2016-08-28	2016	III	clade3_group3	Spain	Valencia	Spain	Resistant	Susceptible	Susceptible	Blood	SRR10461266	Chow 2020	VF125AL	WT
AA-214	Clinical	2016-11-15	2016	III	clade3_group3	Spain	Valencia	Spain	Resistant	Susceptible	Susceptible	Blood	SRR10461265	Chow 2020	VF125AL	WT
B11222	Clinical	2012-10-15	2012	III	clade3_group3	South Africa	Unknown	NA	Resistant	Susceptible	Susceptible	Blood	SRR3883454	Lockhart 2017	VF125AL	WT
B11223	Clinical	2013-01-15	2013	III	clade3_group3	South Africa	Unknown	NA	Resistant	Susceptible	Susceptible	Blood	SRR3883455	Lockhart 2017	VF125AL	WT
B11224	Clinical	2013-10-12	2013	III	clade3_group3	South Africa	Unknown	NA	Resistant	Susceptible	Susceptible	Blood	SRR3883456	Lockhart 2017	VF125AL	WT
B11225	Clinical	2014-04-20	2014	III	clade3_group3	South Africa	Unknown	NA	Resistant	Susceptible	Susceptible	Urine	SRR3883457	Lockhart 2017	VF125AL	WT
B11226	Clinical	2014-03-09	2014	III	clade3_group3	South Africa	Unknown	NA	Resistant	Susceptible	Susceptible	Right leg	SRR3883458	Lockhart 2017	VF125AL	WT
B11227	Clinical	2014-03-16	2014	III	clade3_group3	South Africa	Unknown	NA	Resistant	Susceptible	Susceptible	Blood	SRR3883459	Lockhart 2017	VF125AL	WT
B11228	Clinical	2014-03-18	2014	III	clade3_group3	South Africa	Unknown	NA	Resistant	Susceptible	Susceptible	Hip tissue	SRR3883461	Lockhart 2017	VF125AL	WT
B11229	Clinical	2014-04-21	2014	III	clade3_group3	South Africa	Unknown	NA	Resistant	Susceptible	Susceptible	Urine (catheter)	SRR3883462	Lockhart 2017	VF125AL	WT
B11230	Clinical	2014-03-13	2014	III	clade3_group2	South Africa	Unknown	NA	Resistant	Susceptible	Susceptible	Urine	SRR3883463	Lockhart 2017	WT	WT
B12631	Clinical	2017-03-16	2017	III	clade3_group3	United States	Indiana		Resistant	Susceptible	Susceptible	Wound	SRR7909359	Chow 2018	VF125AL	WT
B16404	Clinical	2017-10-16	2017	III	clade3_group3	Kenya	Unknown	Kenya	Resistant	Susceptible	Resistant	Urine	SRR10461146	Chow 2020	VF125AL	F635dup
B16406	Clinical	2017-10-30	2017	III	clade3_group3	Kenya	Unknown	Kenya	Resistant	Susceptible	Resistant	Urine	SRR10461145	Chow 2020	VF125AL	S639F
B16410	Clinical	2018-06-15	2018	III	clade3_group3	Kenya	Unknown	Kenya	Resistant	Susceptible	Susceptible	Blood	SRR10461144	Chow 2020	VF125AL	WT
B16415	Clinical	2018-07-16	2018	III	clade3_group3	Kenya	Unknown	Kenya	Resistant	Susceptible	Susceptible	Blood	SRR10461143	Chow 2020	VF125AL	WT
B16417	Clinical	2018-06-26	2018	III	clade3_group3	Kenya	Unknown	Kenya	Resistant	Susceptible	Susceptible	Blood	SRR10461142	Chow 2020	VF125AL	WT
B16419	Clinical	2018-04-30	2018	III	clade3_group3	Kenya	Unknown	Kenya	Resistant	Susceptible	Susceptible	Blood	SRR10461141	Chow 2020	VF125AL	WT
B16424	Clinical	2018-05-16	2018	III	clade3_group3	Kenya	Unknown	Kenya	Resistant	Susceptible	Susceptible	Blood	SRR10461138	Chow 2020	VF125AL	WT
B16425	Clinical	2011-11-08	2011	III	clade3_group3	Kenya	Unknown	Kenya	Unknown			blood	SRR10461245	Chow 2020	VF125AL	WT
B16431	Clinical	2013-10-10	2013	III	clade3_group3	Kenya	Unknown	Kenya	Resistant	Susceptible	Susceptible	Blood	SRR10461244	Chow 2020	VF125AL	WT
B16432	Clinical	2013-12-17	2013	III	clade3_group3	Kenya	Unknown	Kenya	Resistant	Susceptible	Susceptible	Blood	SRR10461211	Chow 2020	VF125AL	WT
B16433	Clinical	2013-12-17	2013	III	clade3_group3	Kenya	Unknown	Kenya	Resistant	Susceptible	Susceptible	Blood	SRR10461243	Chow 2020	VF125AL	WT
B16436	Clinical	2014-01-21	2014	III	clade3_group3	Kenya	Unknown	Kenya	Resistant	Susceptible	Susceptible	Blood	SRR10461242	Chow 2020	VF125AL	WT
B16439	Clinical	2014-02-11	2014	III	clade3_group3	Kenya	Unknown	Kenya	Resistant	Susceptible	Susceptible	Blood	SRR10461240	Chow 2020	VF125AL	WT
B16440	Clinical	2014-02-16	2014	III	clade3_group3	Kenya	Unknown	Kenya	Resistant	Susceptible	Resistant	Urine	SRR10461239	Chow 2020	VF125AL	S639Y
B16441	Clinical	2013-10-22	2013	III	clade3_group3	Kenya	Unknown	Kenya	Resistant	Susceptible	Susceptible	Pleural	SRR10461238	Chow 2020	VF125AL	WT
B16445	Clinical	2014-03-21	2014	III	clade3_group3	Kenya	Unknown	Kenya	Resistant	Susceptible	Susceptible	Blood	SRR10461236	Chow 2020	VF125AL	WT
B16451	Clinical	2014-04-07	2014	III	clade3_group3	Kenya	Unknown	Kenya	Resistant	Susceptible	Susceptible	Blood	SRR10461234	Chow 2020	VF125AL	WT
B16454	Clinical	2014-04-15	2014	III	clade3_group3	Kenya	Unknown	Kenya	Resistant	Susceptible	Susceptible	Vascular tip	SRR10461233	Chow 2020	VF125AL	WT
B16457	Clinical	2014-05-22	2014	III	clade3_group3	Kenya	Unknown	Kenya	Resistant	Susceptible	Susceptible	Blood	SRR10461232	Chow 2020	VF125AL	WT
B16459	Clinical	2014-08-16	2014	III	clade3_group3	Kenya	Unknown	Kenya	Resistant	Susceptible	Susceptible	Blood	SRR10461231	Chow 2020	VF125AL	WT
B16461	Clinical	2014-10-08	2014	III	clade3_group3	Kenya	Unknown	Kenya	Resistant	Susceptible	Susceptible	Blood	SRR10461230	Chow 2020	VF125AL	WT
B16466	Clinical	2015-01-21	2015	III	clade3_group3	Kenya	Unknown	Kenya	Resistant	Susceptible	Susceptible	Blood	SRR10461229	Chow 2020	VF125AL	WT
B16467	Clinical	2015-02-02	2015	III	clade3_group3	Kenya	Unknown	Kenya	Resistant	Susceptible	Susceptible	Blood	SRR10461228	Chow 2020	VF125AL	WT
B16469	Clinical	2015-02-09	2015	III	clade3_group3	Kenya	Unknown	Kenya	Resistant	Susceptible	Susceptible	Blood	SRR10461227	Chow 2020	VF125AL	WT
B16473	Clinical	2015-07-27	2015	III	clade3_group3	Kenya	Unknown	Kenya	Resistant	Susceptible	Susceptible	Pleural	SRR10461225	Chow 2020	VF125AL	WT
B16481	Clinical	2016-02-27	2016	III	clade3_group3	Kenya	Unknown	Kenya	Resistant	Susceptible	Susceptible	Blood	SRR10461222	Chow 2020	VF125AL	WT
B16482	Clinical	2016-03-02	2016	III	clade3_group3	Kenya	Unknown	Kenya	Resistant	Susceptible	Susceptible	Blood	SRR10461221	Chow 2020	VF125AL	WT
B16484	Clinical	2016-03-21	2016	III	clade3_group3	Kenya	Unknown	Kenya	Resistant	Susceptible	Resistant	Urine	SRR10461220	Chow 2020	VF125AL	WT
B16485	Clinical	2013-11-05	2013	III	clade3_group3	Kenya	Unknown	Kenya	Resistant	Susceptible	Susceptible	Blood	SRR10461219	Chow 2020	VF125AL	WT
B16491	Clinical	2017-10-02	2017	III	clade3_group3	Kenya	Unknown	Kenya	Resistant	Susceptible	Susceptible	Urine	SRR10461216	Chow 2020	VF125AL	F635dup
B16496	Clinical	2013-11-05	2013	III	clade3_group3	Kenya	Unknown	Kenya	Resistant	Susceptible	Susceptible	Blood	SRR10461215	Chow 2020	VF125AL	WT
B16504	Clinical	2017-10-23	2017	III	clade3_group3	Kenya	Unknown	Kenya	Resistant	Susceptible	Susceptible	Urine	SRR10461213	Chow 2020	VF125AL	WT
B16507	Clinical	2013-12-14	2013	III	clade3_group3	Kenya	Unknown	Kenya	Resistant	Susceptible	Susceptible	Blood	SRR10461212	Chow 2020	VF125AL	WT
B16514	Clinical	2017-07-05	2017	III	clade3_group3	Kenya	Unknown	Kenya	Resistant	Susceptible	Susceptible	Blood	SRR10461207	Chow 2020	VF125AL	WT
B16519	Clinical	2018-03-07	2018	III	clade3_group3	Kenya	Unknown	Kenya	Resistant	Susceptible	Susceptible	Blood	SRR10461205	Chow 2020	VF125AL	WT
B17721	Clinical	2019-02-17	2019	III	clade3_group3	United States	California	US				urine	SRR17577117		VF125AL	WT
B17741	Clinical	2019-03-14	2019	III	clade3_group3	United States	California	US				axilla and groin	SRR17577116		VF125AL	WT
B17742	Clinical	2019-03-14	2019	III	clade3_group3	United States	California	US				axilla and groin	SRR17577105		VF125AL	WT
B17743	Clinical	2019-03-14	2019	III	clade3_group3	United States	California	US				axilla and groin	SRR17577094		VF125AL	WT
B17746	Clinical	2019-03-14	2019	III	clade3_group3	United States	California	US				axilla and groin	SRR17577083		VF125AL	WT
B17806	Clinical	2019-03-28	2019	III	clade3_group3	United States	California	US				not applicable	SRR17577060		VF125AL	WT
B17833	Clinical	2019-04-22	2019	III	clade3_group3	United States	California	US				nose	SRR17577049		VF125AL	WT
B17834	Clinical	2019-04-22	2019	III	clade3_group3	United States	California	US				nose	SRR17577038		VF125AL	WT
B17835	Clinical	2019-04-22	2019	III	clade3_group3	United States	Unknown	US				nose	SRR14252432		VF125AL	WT
B17853	Clinical	2019-04-03	2019	III	clade3_group3	United States	California	US				axilla and groin	SRR17577071		VF125AL	WT
B17854	Clinical	2019-04-03	2019	III	clade3_group3	United States	California	US				axilla and groin	SRR17577070		VF125AL	WT
B17855	Clinical	2019-04-03	2019	III	clade3_group3	United States	California	US				axilla and groin	SRR17577115		VF125AL	WT
B17856	Clinical	2019-04-04	2019	III	clade3_group3	United States	California	US				axilla and groin	SRR17577114		VF125AL	WT
B17857	Clinical	2019-04-08	2019	III	clade3_group3	United States	California	US				nose	SRR17577113		VF125AL	WT
B17858	Clinical	2019-04-08	2019	III	clade3_group3	United States	California	US				axilla and groin	SRR17577112		VF125AL	WT
B17859	Clinical	2019-04-08	2019	III	clade3_group3	United States	California	US				axilla and groin	SRR17577111		VF125AL	WT
B17860	Clinical	2019-04-08	2019	III	clade3_group3	United States	California	US				axilla and groin	SRR17577110		VF125AL	WT
B17861	Clinical	2019-04-08	2019	III	clade3_group3	United States	California	US				axilla and groin	SRR17577109		VF125AL	WT
B17862	Clinical	2019-04-08	2019	III	clade3_group3	United States	California	US				axilla	SRR17577108		VF125AL	WT
B17863	Clinical	2019-04-08	2019	III	clade3_group3	United States	California	US				nose	SRR17577107		VF125AL	WT
B17864	Clinical	2019-04-08	2019	III	clade3_group3	United States	California	US				nose	SRR17577106		VF125AL	WT
B17865	Clinical	2019-04-08	2019	III	clade3_group3	United States	California	US				axilla and groin	SRR17577104		VF125AL	WT
B17866	Clinical	2019-04-08	2019	III	clade3_group3	United States	California	US				axilla and groin	SRR17577103		VF125AL	WT
B17867	Clinical	2019-04-08	2019	III	clade3_group3	United States	California	US				axilla	SRR17577102		VF125AL	WT
B17868	Clinical	2019-04-08	2019	III	clade3_group3	United States	California	US				axilla and groin	SRR17577101		VF125AL	WT
B17869	Clinical	2019-04-08	2019	III	clade3_group3	United States	California	US				axilla and groin	SRR17577100		VF125AL	WT
B17870	Clinical	2019-04-08	2019	III	clade3_group3	United States	California	US				axilla and groin	SRR17577099		VF125AL	WT
B17871	Clinical	2019-04-11	2019	III	clade3_group3	United States	California	US				axilla and groin	SRR17577098		VF125AL	WT
B17872	Clinical	2019-04-11	2019	III	clade3_group3	United States	California	US				nose	SRR17577097		VF125AL	WT
B17873	Clinical	2019-04-03	2019	III	clade3_group3	United States	California	US				nose	SRR17577096		VF125AL	WT
B17874	Clinical	2019-04-03	2019	III	clade3_group3	United States	California	US				nose	SRR17577095		VF125AL	WT
B17875	Clinical	2019-05-07	2019	III	clade3_group3	United States	California	US				nose	SRR17577093		VF125AL	WT
B17876	Clinical	2019-05-07	2019	III	clade3_group3	United States	California	US				nose	SRR17577092		VF125AL	WT
B17877	Clinical	2019-05-09	2019	III	clade3_group3	United States	California	US				nose	SRR17577091		VF125AL	WT
B17878	Clinical	2019-05-09	2019	III	clade3_group3	United States	California	US				nose	SRR17577090		VF125AL	WT
B17916	Clinical	2019-05-07	2019	III	clade3_group3	United States	California	US				bronchial washings	SRR17577089		VF125AL	WT
B17924	Clinical	2019-05-04	2019	III	clade3_group3	United States	California	US				blood	SRR17577088		VF125AL	WT
B17937	Clinical	2019-05-29	2019	III	clade3_group3	United States	California	US				axilla and groin	SRR17577087		VF125AL	WT
B17948	Clinical	2019-05-15	2019	III	clade3_group3	United States	California	US				axilla and groin	SRR17577086		VF125AL	WT
B17949	Clinical	2019-05-15	2019	III	clade3_group3	United States	California	US				axilla and groin	SRR17577085		VF125AL	WT
B17950	Clinical	2019-05-15	2019	III	clade3_group3	United States	California	US				axilla and groin	SRR17577084		VF125AL	WT
B18157	Clinical	2019-07-04	2019	III	clade3_group3	United States	California	US				urine	SRR17577082		VF125AL	WT

B18158	Clinical	2019-07-07	2019	III	clade3_group3	United States	California	US				foreign object (body)	SRR17577069		VF125AL	WT
B18159	Clinical	2019-07-17	2019	III	clade3_group3	United States	California	US				axilla and groin	SRR17577068		VF125AL	WT
B18160	Clinical	2019-07-15	2019	III	clade3_group3	United States	California	US				blood	SRR17577067		VF125AL	WT
B18225	Clinical	2019-07-17	2019	III	clade3_group3	United States	California	US				urine	SRR17577066		VF125AL	WT
B18226	Clinical	2019-07-29	2019	III	clade3_group3	United States	California	US				sputum	SRR17577065		VF125AL	WT
B18227	Clinical	2019-07-24	2019	III	clade3_group3	United States	California	US				urine	SRR17577064		VF125AL	WT
B18249	Clinical	2019-08-13	2019	III	clade3_group3	United States	California	US				axilla and groin	SRR17577063		VF125AL	WT
B18263	Clinical	2019-08-28	2019	III	clade3_group3	United States	California	US				axilla and groin	SRR17577062		VF125AL	WT
B18278	Clinical	2019-09-11	2019	III	clade3_group3	United States	California	US				axilla and groin	SRR17577061		VF125AL	WT
B18289	Clinical	2019-08-22	2019	III	clade3_group3	United States	California	US				blood	SRR17577059		VF125AL	WT
B18458	Clinical	2019-09-25	2019	III	clade3_group3	United States	California	US				axilla and groin	SRR17577058		VF125AL	WT
B18460	Clinical	2019-09-01	2019	III	clade3_group3	United States	California	US				blood	SRR17577057		VF125AL	WT
B18461	Clinical	2019-08-17	2019	III	clade3_group3	United States	California	US				ear	SRR17577056		VF125AL	WT
B18532	Clinical	2020-07-07	2020	III	clade3_group3	United States	Florida		Unknown	Unknown	Unknown	Sputum	SRR12073435	CDC Mycotic Diseases Branch Canc	VF125AL	WT
B18533	Clinical	2020-07-07	2020	III	clade3_group3	United States	Florida		Unknown	Unknown	Unknown	Blood	SRR12073484	CDC Mycotic Diseases Branch Canc	VF125AL	WT
B18540	Clinical	2019-09-15	2019	III	clade3_group3	United States	California	US				blood	SRR17577055		VF125AL	WT
B18657	Clinical	2019-10-01	2019	III	clade3_group3	United States	California	US				axilla and groin	SRR17577054		VF125AL	WT
B18665	Clinical	2020-07-07	2020	III	clade3_group3	United States	Florida		Unknown	Unknown	Unknown	Axilla/Groin swab	SRR12073481	CDC Mycotic Diseases Branch Canc	VF125AL	WT
B18683	Clinical	2019-11-25	2019	III	clade3_group3	Kenya	Unknown	NA				skin swab	SRR14252431		VF125AL	WT
B19285	Clinical	2020-09-03	2020	III	clade3_group3	United States	Unknown					urine	SRR14590379		VF125AL	WT
B19448	Clinical	2020-10-02	2020	III	clade3_group3	United States	Unknown					leg	SRR14590390		VF125AL	WT
B19584	Clinical	2020-10-29	2020	III	clade3_group3	United States	Unknown					not applicable	SRR14590383		VF125AL	WT
B19913	Clinical	2020-11-16	2020	III	clade3_group3	United States	Unknown					blood	SRR14590389		VF125AL	WT
B19920	Clinical	2021-01-04	2021	III	clade3_group3	United States	Unknown					urine	SRR14590388		VF125AL	WT
B19944	Clinical	2021-01-24	2021	III	clade3_group3	United States	Unknown					sputum	SRR14590384		VF125AL	WT
B19983	Clinical	2021-01-19	2021	III	clade3_group3	United States	Unknown					wound	SRR14590386		VF125AL	WT
B19985	Clinical	2021-01-15	2021	III	clade3_group3	United States	Unknown					wound	SRR14590385		VF125AL	WT
C12-A109	Environmental	2018-03-09	2018	III	clade3_group3	China	Shenyang	China	Resistant	Susceptible	Susceptible	Environmental	SRR9316725	Tian 2021	VF125AL	WT
C20955	Clinical	2019-04-18	2019	III	clade3_group3	United States	California	US				axilla and groin	SRR17577053		VF125AL	WT
C21072	Clinical	2019-04-22	2019	III	clade3_group3	United States	California	US				axilla and groin	SRR17577052		VF125AL	WT
C21086	Clinical	2019-04-22	2019	III	clade3_group3	United States	California	US				axilla and groin	SRR17577051		VF125AL	WT
C21485	Clinical	2019-04-25	2019	III	clade3_group3	United States	California	US				axilla and groin	SRR17577050		VF125AL	WT
C45587	Clinical	2019-05-07	2019	III	clade3_group3	United States	California	US				axilla and groin	SRR17577048		VF125AL	WT
C45616	Clinical	2019-05-07	2019	III	clade3_group3	United States	California	US				axilla and groin	SRR17577047		VF125AL	WT
C45954	Clinical	2019-05-08	2019	III	clade3_group3	United States	California	US				axilla and groin	SRR17577046		VF125AL	WT
C45960	Clinical	2019-05-08	2019	III	clade3_group3	United States	California	US				axilla and groin	SRR17577045		VF125AL	WT
C45964	Clinical	2019-05-08	2019	III	clade3_group3	United States	California	US				axilla and groin	SRR17577044		VF125AL	WT
C45965	Clinical	2019-05-08	2019	III	clade3_group3	United States	California	US				axilla and groin	SRR17577043		VF125AL	WT
C45969	Clinical	2019-05-08	2019	III	clade3_group3	United States	California	US				axilla and groin	SRR17577042		VF125AL	WT
C45991	Clinical	2019-05-08	2019	III	clade3_group3	United States	California	US				axilla and groin	SRR17577041		VF125AL	WT
C46002	Clinical	2019-05-08	2019	III	clade3_group3	United States	California	US				axilla and groin	SRR17577040		VF125AL	WT
C46008	Clinical	2019-05-08	2019	III	clade3_group3	United States	California	US				axilla and groin	SRR17577039		VF125AL	WT
C46010	Clinical	2019-05-08	2019	III	clade3_group3	United States	California	US				axilla and groin	SRR17577081		VF125AL	WT
C46013	Clinical	2019-05-08	2019	III	clade3_group3	United States	California	US				axilla and groin	SRR17577080		VF125AL	WT
C46014	Clinical	2019-05-08	2019	III	clade3_group3	United States	California	US				axilla and groin	SRR17577079		VF125AL	WT
C46015	Clinical	2019-05-08	2019	III	clade3_group3	United States	California	US				axilla and groin	SRR17577078		VF125AL	WT
C46020	Clinical	2019-05-08	2019	III	clade3_group3	United States	California	US				axilla and groin	SRR17577077		VF125AL	WT
C46022	Clinical	2019-05-08	2019	III	clade3_group3	United States	California	US				axilla and groin	SRR17577076		VF125AL	WT
C46027	Clinical	2019-05-08	2019	III	clade3_group3	United States	California	US				axilla and groin	SRR17577075		VF125AL	WT
C46062	Clinical	2019-05-09	2019	III	clade3_group3	United States	California	US				axilla and groin	SRR17577074		VF125AL	WT
C46107	Clinical	2019-05-09	2019	III	clade3_group3	United States	California	US				axilla and groin	SRR17577073		VF125AL	WT
C46121	Clinical	2019-05-09	2019	III	clade3_group3	United States	California	US				axilla and groin	SRR17577072		VF125AL	WT
CA-OCPHL-CAU	Clinical	2019-07-29	2019	III	clade3_group3	United States	California	US				sputum	SRR24806737		VF125AL	WT
CA-OCPHL-CAU	Clinical	2021-04-27	2021	III	clade3_group3	United States	California	US				blood	SRR24806704		VF125AL	WT
CA-OCPHL-CAU	Clinical	2021-05-07	2021	III	clade3_group3	United States	California	US				urine	SRR24806760		VF125AL	S639F
CA-OCPHL-CAU	Clinical	2021-05-12	2021	III	clade3_group3	United States	California	US				urine	SRR24806759		VF125AL	WT
CA-OCPHL-CAU	Clinical	2021-05-28	2021	III	clade3_group3	United States	California	US				body fluid	SRR24806693		VF125AL	WT
CA-OCPHL-CAU	Clinical	2021-07-30	2021	III	clade3_group3	United States	California	US				urine	SRR24806763		VF125AL	WT
CA-OCPHL-CAU	Clinical	2021-09-03	2021	III	clade3_group3	United States	California	US				wound	SRR24806764		VF125AL	WT
CA-OCPHL-CAU	Clinical	2021-09-28	2021	III	clade3_group3	United States	California	US				urine	SRR24806761		VF125AL	WT
CA-OCPHL-CAU	Clinical	2021-10-20	2021	III	clade3_group3	United States	California	US				blood	SRR24806767		VF125AL	WT
CA-OCPHL-CAU	Clinical	2021-11-11	2021	III	clade3_group3	United States	California	US				Tissue	SRR24806715		VF125AL	WT
CA-OCPHL-CAU	Clinical	2021-11-20	2021	III	clade3_group3	United States	California	US				urine	SRR24806766		VF125AL	WT
CA-OCPHL-CAU	Clinical	2021-12-16	2021	III	clade3_group3	United States	California	US				urine	SRR24806748		VF125AL	WT
CA-OCPHL-CAU	Clinical	2022-01-02	2022	III	clade3_group3	United States	California	US				blood	SRR24806783		VF125AL	WT
CA-OCPHL-CAU	Clinical	2022-01-09	2022	III	clade3_group3	United States	California	US				blood	SRR24806682		VF125AL	WT
CA-OCPHL-CAU	Clinical	2022-07-01	2022	III	clade3_group3	United States	California	US				urine	SRR24806765		VF125AL	WT
CA-OCPHL-CAU	Clinical	2022-08-06	2022	III	clade3_group3	United States	California	US				blood	SRR24806784		VF125AL	WT
CA-OCPHL-CAU	Clinical	2022-10-13	2022	III	clade3_group3	United States	California	US				Tissue	SRR24806726		VF125AL	WT
CA-OCPHL-CAU	Clinical	2022-11-10	2022	III	clade3_group3	United States	California	US				wound	SRR24806762		VF125AL	WT
CA-OCPHL-CAU	Clinical	2022-01-03	2022	III	clade3_group3	United States	California	US				wound	SRR24806755		VF125AL	WT
CA-OCPHL-CAU	Clinical	2022-05-10	2022	III	clade3_group3	United States	California	US				BAL	SRR24806757		VF125AL	WT
CA-OCPHL-CAU	Clinical	2022-06-16	2022	III	clade3_group3	United States	California	US				sputum	SRR24806758		VF125AL	WT
CA-OCPHL-CAU	Clinical	2022-07-09	2022	III	clade3_group3	United States	California	US				body fluid	SRR24806754		VF125AL	WT
CA-OCPHL-CAU	Clinical	2022-08-25	2022	III	clade3_group3	United States	California	US				urine	SRR24806752		VF125AL	WT
CA-OCPHL-CAU	Clinical	2022-11-06	2022	III	clade3_group3	United States	California	US				Tissue	SRR24806753		VF125AL	WT
CA-OCPHL-CAU	Clinical	2021-07-14	2021	III	clade3_group3	United States	California	US				Axilla/Groin	SRR24806751		VF125AL	WT
CA-OCPHL-CAU	Clinical	2022-06-07	2022	III	clade3_group3	United States	California	US				wound	SRR24806750		VF125AL	WT
CA-OCPHL-CAU	Clinical	2022-10-07	2022	III	clade3_group3	United States	California	US				blood	SRR24806749		VF125AL	WT
CA-OCPHL-CAU	Clinical	2022-06-03	2022	III	clade3_group3	United States	California	US				wound	SRR24806747		VF125AL	WT
CA-OCPHL-CAU	Clinical	2022-12-18	2022	III	clade3_group3	United States	California	US				Axilla/Groin	SRR24806741		VF125AL	WT
CA-OCPHL-CAU	Clinical	2022-12-18	2022	III	clade3_group3	United States	California	US				Axilla/Groin	SRR24806731		VF125AL	WT
CA-OCPHL-CAU	Clinical	2022-12-04	2022	III	clade3_group3	United States	California	US				Axilla/Groin	SRR24806738		VF125AL	WT
CA-OCPHL-CAU	Clinical	2022-11-02	2022	III	clade3_group3	United States	California	US				Axilla/Groin	SRR24806743		VF125AL	WT
CA-OCPHL-CAU	Clinical	2022-11-01	2022	III	clade3_group3	United States	California	US				Axilla/Groin	SRR24806740		VF125AL	WT
CA-OCPHL-CAU	Clinical	2022-10-19	2022	III	clade3_group3	United States	California	US				Axilla/Groin	SRR24806742		VF125AL	WT
CA-OCPHL-CAU	Clinical	2022-10-19	2022	III	clade3_group3	United States	California	US				Axilla/Groin	SRR24806736		VF125AL	WT
CA-OCPHL-CAU	Clinical	2022-09-13	2022	III	clade3_group3	United States	California	US				Axilla/Groin	SRR24806735		VF125AL	WT
CA-OCPHL-CAU	Clinical	2022-02-15	2022	III	clade3_group3	United States	California	US				Axilla/Groin	SRR24806744		VF125AL	WT
CA-OCPHL-CAU	Clinical	2022-10-26	2022	III	clade3_group3	United States	California	US				Axilla/Groin	SRR24806728		VF125AL	WT
CA-OCPHL-CAU	Clinical	2023-01-04	2023	III	clade3_group3	United States	California	US				Axilla/Groin	SRR24806745		VF125AL	WT
CA-OCPHL-CAU	Clinical	2023-01-04	2023	III	clade3_group3	United States	California	US				Axilla/Groin	SRR24806734		VF125AL	WT

CA-OCPHL-CAU	Clinical	2022-09-06	2022	III	clade3_group3	United States	California	US				Axilla/Groin	SRR24806727		VF125AL	WT
CA-OCPHL-CAU	Clinical	2022-06-20	2022	III	clade3_group3	United States	California	US				Axilla/Groin	SRR24806732		VF125AL	WT
CA-OCPHL-CAU	Clinical	2022-05-19	2022	III	clade3_group3	United States	California	US				Axilla/Groin	SRR24806730		VF125AL	WT
CA-OCPHL-CAU	Clinical	2022-03-28	2022	III	clade3_group3	United States	California	US				Axilla/Groin	SRR24806729		VF125AL	WT
CA-OCPHL-CAU	Clinical	2022-03-28	2022	III	clade3_group3	United States	California	US				Axilla/Groin	SRR24806739		VF125AL	WT
CA-OCPHL-CAU	Clinical	2022-03-28	2022	III	clade3_group3	United States	California	US				Axilla/Groin	SRR24806746		VF125AL	WT
CA-OCPHL-CAU	Clinical	2023-01-17	2023	III	clade3_group3	United States	California	US				Axilla/Groin	SRR24806733		VF125AL	WT
CA-OCPHL-CAU	Clinical	2022-04-08	2022	III	clade3_group3	United States	California	US				wound	SRR24806725		VF125AL	WT
CA-OCPHL-CAU	Clinical	2022-09-28	2022	III	clade3_group3	United States	California	US				Peg site	SRR24806724		VF125AL	WT
CA-OCPHL-CAU	Clinical	2023-01-04	2023	III	clade3_group3	United States	California	US				Tissue	SRR24806723		VF125AL	WT
CA-OCPHL-CAU	Clinical	2021-12-18	2021	III	clade3_group3	United States	California	US				wound	SRR24806722		VF125AL	WT
CA-OCPHL-CAU	Clinical	2022-02-27	2022	III	clade3_group3	United States	California	US				urine	SRR24806721		VF125AL	WT
CA-OCPHL-CAU	Clinical	2022-05-28	2022	III	clade3_group3	United States	California	US				urine	SRR24806720		VF125AL	WT
CA-OCPHL-CAU	Clinical	2022-07-15	2022	III	clade3_group3	United States	California	US				urine	SRR24806718		VF125AL	WT
CA-OCPHL-CAU	Clinical	2022-10-19	2022	III	clade3_group3	United States	California	US				wound	SRR24806717		VF125AL	WT
CA-OCPHL-CAU	Clinical	2022-11-13	2022	III	clade3_group3	United States	California	US				Axilla/Groin	SRR24806716		VF125AL	WT
CA-OCPHL-CAU	Clinical	2022-11-14	2022	III	clade3_group3	United States	California	US				Axilla/Groin	SRR24806714		VF125AL	WT
CA-OCPHL-CAU	Clinical	2023-01-01	2023	III	clade3_group3	United States	California	US				Tissue	SRR24806713		VF125AL	WT
CA-OCPHL-CAU	Clinical	2022-10-23	2022	III	clade3_group3	United States	California	US				urine	SRR24806712		VF125AL	WT
CA-OCPHL-CAU	Clinical	2022-09-20	2022	III	clade3_group3	United States	California	US				urine	SRR24806710		VF125AL	WT
CA-OCPHL-CAU	Clinical	2021-06-26	2021	III	clade3_group3	United States	California	US				tracheal aspirate	SRR24806708		VF125AL	WT
CA-OCPHL-CAU	Clinical	2021-07-19	2021	III	clade3_group3	United States	California	US				Resp Processed	SRR24806707		VF125AL	WT
CA-OCPHL-CAU	Clinical	2021-07-31	2021	III	clade3_group3	United States	California	US				wound	SRR24806706		VF125AL	WT
CA-OCPHL-CAU	Clinical	2021-09-11	2021	III	clade3_group3	United States	California	US				tracheal aspirate	SRR24806705		VF125AL	WT
CA-OCPHL-CAU	Clinical	2021-10-11	2021	III	clade3_group3	United States	California	US				urine	SRR24806703		VF125AL	WT
CA-OCPHL-CAU	Clinical	2021-10-21	2021	III	clade3_group3	United States	California	US				urine	SRR24806702		VF125AL	WT
CA-OCPHL-CAU	Clinical	2021-10-29	2021	III	clade3_group3	United States	California	US				urine	SRR24806701		VF125AL	WT
CA-OCPHL-CAU	Clinical	2021-12-03	2021	III	clade3_group3	United States	California	US				blood	SRR24806700		VF125AL	WT
CA-OCPHL-CAU	Clinical	2021-12-17	2021	III	clade3_group3	United States	California	US				blood	SRR24806699		VF125AL	WT
CA-OCPHL-CAU	Clinical	2021-12-29	2021	III	clade3_group3	United States	California	US				Catheter	SRR24806698		VF125AL	WT
CA-OCPHL-CAU	Clinical	2022-02-24	2022	III	clade3_group3	United States	California	US				urine	SRR24806697		VF125AL	WT
CA-OCPHL-CAU	Clinical	2022-04-15	2022	III	clade3_group3	United States	California	US				blood	SRR24806696		VF125AL	WT
CA-OCPHL-CAU	Clinical	2022-04-27	2022	III	clade3_group3	United States	California	US				blood	SRR24806695		VF125AL	WT
CA-OCPHL-CAU	Clinical	2022-07-11	2022	III	clade3_group3	United States	California	US				Respiratory Process	SRR24806709		VF125AL	WT
CA-OCPHL-CAU	Clinical	2022-09-30	2022	III	clade3_group3	United States	California	US				blood	SRR24806694		VF125AL	WT
CA-OCPHL-CAU	Clinical	2021-08-28	2021	III	clade3_group3	United States	California	US				Axilla/Groin	SRR24806692		VF125AL	WT
CA-OCPHL-CAU	Clinical	2021-09-17	2021	III	clade3_group3	United States	California	US				Axilla/Groin	SRR24806691		VF125AL	WT
CA-OCPHL-CAU	Clinical	2021-10-01	2021	III	clade3_group3	United States	California	US				wound	SRR24806690		VF125AL	WT
CA-OCPHL-CAU	Clinical	2021-11-17	2021	III	clade3_group3	United States	California	US				urine	SRR24806689		VF125AL	WT
CA-OCPHL-CAU	Clinical	2021-11-22	2021	III	clade3_group3	United States	California	US				Axilla/Groin	SRR24806688		VF125AL	WT
CA-OCPHL-CAU	Clinical	2022-02-08	2022	III	clade3_group3	United States	California	US				urine	SRR24806687		VF125AL	WT
CA-OCPHL-CAU	Clinical	2022-02-12	2022	III	clade3_group3	United States	California	US				blood	SRR24806686		VF125AL	WT
CA-OCPHL-CAU	Clinical	2022-03-01	2022	III	clade3_group3	United States	California	US				urine	SRR24806685		VF125AL	WT
CA-OCPHL-CAU	Clinical	2022-05-13	2022	III	clade3_group3	United States	California	US				wound	SRR24806684		VF125AL	WT
CA-OCPHL-CAU	Clinical	2022-05-26	2022	III	clade3_group3	United States	California	US				Axilla/Groin	SRR24806683		VF125AL	WT
CA-OCPHL-CAU	Clinical	2022-05-30	2022	III	clade3_group3	United States	California	US				Tissue	SRR24806681		VF125AL	WT
CA-OCPHL-CAU	Clinical	2022-06-01	2022	III	clade3_group3	United States	California	US				Bronch Wash	SRR24806680		VF125AL	WT
CA-OCPHL-CAU	Clinical	2022-07-01	2022	III	clade3_group3	United States	California	US				Axilla/Groin	SRR24806679		VF125AL	WT
CA-OCPHL-CAU	Clinical	2022-07-08	2022	III	clade3_group3	United States	California	US				urine	SRR24806678		VF125AL	WT
CA-OCPHL-CAU	Clinical	2022-08-27	2022	III	clade3_group3	United States	California	US				Axilla/Groin	SRR24806677		VF125AL	WT
CA-OCPHL-CAU	Clinical	2022-09-07	2022	III	clade3_group3	United States	California	US				sputum	SRR24806676		VF125AL	WT
CA-OCPHL-CAU	Clinical	2022-12-30	2022	III	clade3_group3	United States	California	US				urine	SRR24806675		VF125AL	WT
CA-OCPHL-CAU	Clinical	2022-12-10	2022	III	clade3_group3	United States	California	US				blood	SRR24806674		VF125AL	WT
CA-OCPHL-CAU	Clinical	2021-09-14	2021	III	clade3_group3	United States	California	US				Axilla/Groin	SRR24806673		VF125AL	WT
CA-OCPHL-CAU	Clinical	2023-01-30	2023	III	clade3_group3	United States	California	US				urine	SRR24806672		VF125AL	WT
CA-OCPHL-CAU	Clinical	2022-11-21	2022	III	clade3_group3	United States	California	US				urine	SRR24806781		VF125AL	WT
CA-OCPHL-CAU	Clinical	2023-02-17	2023	III	clade3_group3	United States	California	US				urine	SRR24806780		VF125AL	WT
CA-OCPHL-CAU	Clinical	2023-02-10	2023	III	clade3_group3	United States	California	US				wound	SRR24806779		VF125AL	WT
CA-OCPHL-CAU	Clinical	2023-01-22	2023	III	clade3_group3	United States	California	US				urine	SRR24806778		VF125AL	WT
CA-OCPHL-CAU	Clinical	2022-11-28	2022	III	clade3_group3	United States	California	US				sputum	SRR24806773		VF125AL	WT
CA-OCPHL-CAU	Clinical	2022-12-12	2022	III	clade3_group3	United States	California	US				blood	SRR24806776		VF125AL	WT
CA-OCPHL-CAU	Clinical	2023-03-16	2023	III	clade3_group3	United States	California	US				wound	SRR24806775		VF125AL	WT
CA-OCPHL-CAU	Clinical	2022-11-02	2022	III	clade3_group3	United States	California	US				urine	SRR24806770		VF125AL	WT
CA-OCPHL-CAU	Clinical	2020-05-24	2020	III	clade3_group3	United States	California	US				urine	SRR24806768		VF125AL	WT
CA-OCPHL-CAU	Clinical	2023-02-21	2023	III	clade3_group3	United States	California	US				urine	SRR24806769		VF125AL	WT
CA-S97	Clinical	2015-07-15	2015	III	clade3_group3	Australia	Unknown	NA	Resistant	Susceptible	Susceptible	Bone	SRR7657927	Heath 2019	VF125AL	WT
Cau4	Clinical	2021-10-01	2021	III	clade3_group3	Austria	Unknown	Spain	Resistant	Susceptible	Susceptible	Throat	SRR2308087	Spettel 2023	VF125AL	WT
CT-WCPHL-CAU	Clinical	2020-09-08	2020	III	clade3_group3	United States	Connecticut						SRR24621173		VF125AL	WT
GCF_002775015	Clinical	2012-10-23	2012	III	clade3_group3	South Africa	Unknown	NA	Resistant	Susceptible	Susceptible	Blood		Muñoz 2018	VF125AL	WT
Hoist	Environmental	2017-04-04	2017	III	clade3_group3	United Kingdom	Oxfordshire	UK	Unknown	Unknown	Unknown	Environmental	SRR7976583	Eyre 2018	VF125AL	WT
NICU10-A58	Clinical	2018-02-03	2018	III	clade3_group3	China	Shenyang	China	Resistant	Susceptible	Susceptible	Urine	SRR9316776	Tian 2021	VF125AL	WT
NICU11-A77	Clinical	2018-05-12	2018	III	clade3_group3	China	Shenyang	China	Resistant	Susceptible	Susceptible	Blood	SRR9316803	Tian 2021	VF125AL	WT
NICU12-A79	Clinical	2018-05-15	2018	III	clade3_group3	China	Shenyang	China	Resistant	Susceptible	Susceptible	Urine	SRR9316800	Tian 2021	VF125AL	WT
NICU13-A93	Clinical	2018-08-10	2018	III	clade3_group3	China	Shenyang	China	Resistant	Susceptible	Susceptible	Urine	SRR9316812	Tian 2021	VF125AL	WT
NICU14-A96	Clinical	2018-08-27	2018	III	clade3_group3	China	Shenyang	China	Resistant	Susceptible	Susceptible	Urine	SRR9316734	Tian 2021	VF125AL	WT
NICU15-A100	Clinical	2018-09-04	2018	III	clade3_group3	China	Shenyang	China	Resistant	Susceptible	Susceptible	Urine	SRR9316790	Tian 2021	VF125AL	WT
NICU16-A101	Clinical	2018-09-04	2018	III	clade3_group3	China	Shenyang	China	Resistant	Susceptible	Susceptible	Urine	SRR9316791	Tian 2021	VF125AL	WT
NICU2-A13	Clinical	2016-12-24	2016	III	clade3_group3	China	Shenyang	China	Resistant	Susceptible	Susceptible	Urine	SRR9316739	Tian 2021	VF125AL	WT
NICU3-A15	Clinical	2017-02-04	2017	III	clade3_group3	China	Shenyang	China	Resistant	Susceptible	Susceptible	Urine	SRR9316741	Tian 2021	VF125AL	WT
NICU4-A19	Clinical	2017-03-17	2017	III	clade3_group3	China	Shenyang	China	Resistant	Susceptible	Susceptible	Urine	SRR9316745	Tian 2021	VF125AL	WT
NICU5-A36	Clinical	2017-09-30	2017	III	clade3_group3	China	Shenyang	China	Resistant	Susceptible	Susceptible	Urine	SRR9316756	Tian 2021	VF125AL	WT
NICU6-A41	Clinical	2017-10-17	2017	III	clade3_group3	China	Shenyang	China	Resistant	Susceptible	Susceptible	Fluid	SRR9316762	Tian 2021	VF125AL	WT
NICU7-A42	Clinical	2017-11-22	2017	III	clade3_group3	China	Shenyang	China	Resistant	Susceptible	Susceptible	Urine	SRR9316761	Tian 2021	VF125AL	WT
NICU8-A43	Clinical	2017-11-24	2017	III	clade3_group3	China	Shenyang	China	Resistant	Susceptible	Susceptible	Urine	SRR9316760	Tian 2021	VF125AL	WT
NICU9-A50	Clinical	2017-12-12	2017	III	clade3_group3	China	Shenyang	China	Resistant	Susceptible	Susceptible	Urine	SRR9316770	Tian 2021	VF125AL	WT
NSICU1-A24	Clinical	2017-08-24	2017	III	clade3_group3	China	Shenyang	China	Resistant	Susceptible	Susceptible	Urine	SRR9316751	Tian 2021	VF125AL	WT
NY-WCPHL-CAU	Clinical	2023-01-01	2022	III	clade3_group3	United States	New York	US				Wound finger	SRR24422757		VF125AL	WT
NY-WCPHL-CAU	Clinical	2019-10-11	2019	III	clade3_group3	United States	New York					Peritoneal dialysis cathet	SRR24621174		VF125AL	WT
NY-WCPHL-CAU	Clinical	2022-11-01	2022	III	clade3_group3	United States	New York					Blood (peripheral)	SRR24422751		VF125AL	WT
NY-WCPHL-CAU	Clinical	2020-09-25	2020	III	clade3_group3	United States	New York					wound	SRR24621161		VF125AL	WT
Patient-001	Clinical	2015-02-02	2015	III	clade3_group3	United Kingdom	Oxfordshire	UK	Unknown	Unknown	Unknown	clinical sample	SRR7976549	Eyre 2018	VF125AL	WT
Patient-002	Clinical	2015-07-10	2015	III	clade3_group3	United Kingdom	Oxfordshire	UK	Unknown	Unknown	Unknown	clinical sample	SRR7976579	Eyre 2018	VF125AL	WT

Patient-003	Clinical	2015-07-13	2015	III	clade3_group3	United Kingdom	Oxfordshire	UK	Unknown	Unknown	Unknown	clinical sample	SRR7976616	Eyre 2018		VF125AL	WT
Patient-004	Clinical	2015-11-24	2015	III	clade3_group3	United Kingdom	Oxfordshire	UK	Unknown	Unknown	Unknown	clinical sample	SRR7976582	Eyre 2018		VF125AL	WT
Patient-005	Clinical	2016-01-02	2016	III	clade3_group3	United Kingdom	Oxfordshire	UK	Unknown	Unknown	Unknown	clinical sample	SRR7976614	Eyre 2018		VF125AL	WT
Patient-006-01	Clinical	2016-10-25	2016	III	clade3_group3	United Kingdom	Oxfordshire	UK	Unknown	Unknown	Unknown	clinical sample	SRR7976557	Eyre 2018		VF125AL	WT
Patient-007	Clinical	2016-11-17	2016	III	clade3_group3	United Kingdom	Oxfordshire	UK	Unknown	Unknown	Unknown	clinical sample	SRR7976542	Eyre 2018		VF125AL	WT
Patient-008-01	Clinical	2016-11-28	2016	III	clade3_group3	United Kingdom	Oxfordshire	UK	Unknown	Unknown	Unknown	clinical sample	SRR7976584	Eyre 2018		VF125AL	WT
Patient-009-01	Clinical	2016-12-05	2016	III	clade3_group3	United Kingdom	Oxfordshire	UK	Unknown	Unknown	Unknown	clinical sample	SRR7976597	Eyre 2018		VF125AL	WT
Patient-010-01	Clinical	2016-12-11	2016	III	clade3_group3	United Kingdom	Oxfordshire	UK	Unknown	Unknown	Unknown	clinical sample	SRR7976591	Eyre 2018		VF125AL	WT
Patient-011-01	Clinical	2016-12-19	2016	III	clade3_group3	United Kingdom	Oxfordshire	UK	Unknown	Unknown	Unknown	clinical sample	SRR7976577	Eyre 2018		VF125AL	WT
Patient-012-01	Clinical	2017-01-09	2017	III	clade3_group3	United Kingdom	Oxfordshire	UK	Unknown	Unknown	Unknown	clinical sample	SRR7976604	Eyre 2018		VF125AL	WT
Patient-013-01	Clinical	2017-01-10	2017	III	clade3_group3	United Kingdom	Oxfordshire	UK	Unknown	Unknown	Unknown	clinical sample	SRR7976564	Eyre 2018		VF125AL	WT
Patient-015-01	Clinical	2017-01-25	2017	III	clade3_group3	United Kingdom	Oxfordshire	UK	Unknown	Unknown	Unknown	clinical sample	SRR7976599	Eyre 2018		VF125AL	WT
Patient-016	Clinical	2017-01-27	2017	III	clade3_group3	United Kingdom	Oxfordshire	UK	Unknown	Unknown	Unknown	clinical sample	SRR7976578	Eyre 2018		VF125AL	WT
Patient-017	Clinical	2017-02-10	2017	III	clade3_group3	United Kingdom	Oxfordshire	UK	Unknown	Unknown	Unknown	clinical sample	SRR7976540	Eyre 2018		VF125AL	WT
Patient-019-01	Clinical	2017-02-17	2017	III	clade3_group3	United Kingdom	Oxfordshire	UK	Unknown	Unknown	Unknown	clinical sample	SRR7976552	Eyre 2018		VF125AL	WT
Patient-021-01	Clinical	2017-03-08	2017	III	clade3_group3	United Kingdom	Oxfordshire	UK	Unknown	Unknown	Unknown	clinical sample	SRR7976548	Eyre 2018		VF125AL	WT
Patient-022-01	Clinical	2017-03-10	2017	III	clade3_group3	United Kingdom	Oxfordshire	UK	Unknown	Unknown	Unknown	clinical sample	SRR7976572	Eyre 2018		VF125AL	WT
Patient-023	Clinical	2017-03-18	2017	III	clade3_group3	United Kingdom	Oxfordshire	UK	Unknown	Unknown	Unknown	clinical sample	SRR7976553	Eyre 2018		VF125AL	WT
Patient-024-01	Clinical	2017-03-24	2017	III	clade3_group3	United Kingdom	Oxfordshire	UK	Unknown	Unknown	Unknown	clinical sample	SRR7976592	Eyre 2018		VF125AL	WT
Patient-025-01	Clinical	2017-03-27	2017	III	clade3_group3	United Kingdom	Oxfordshire	UK	Unknown	Unknown	Unknown	clinical sample	SRR7976565	Eyre 2018		VF125AL	WT
Patient-026	Clinical	2017-03-29	2017	III	clade3_group3	United Kingdom	Oxfordshire	UK	Unknown	Unknown	Unknown	clinical sample	SRR7976600	Eyre 2018		VF125AL	WT
Patient-027-01	Clinical	2017-03-31	2017	III	clade3_group3	United Kingdom	Oxfordshire	UK	Unknown	Unknown	Unknown	clinical sample	SRR7976615	Eyre 2018		VF125AL	WT
Patient-028-01	Clinical	2017-04-03	2017	III	clade3_group3	United Kingdom	Oxfordshire	UK	Unknown	Unknown	Unknown	clinical sample	SRR7976585	Eyre 2018		VF125AL	WT
Patient-029-01	Clinical	2017-04-07	2017	III	clade3_group3	United Kingdom	Oxfordshire	UK	Unknown	Unknown	Unknown	clinical sample	SRR7976558	Eyre 2018		VF125AL	WT
Patient-030	Clinical	2017-04-09	2017	III	clade3_group3	United Kingdom	Oxfordshire	UK	Unknown	Unknown	Unknown	clinical sample	SRR7976598	Eyre 2018		VF125AL	WT
Patient-031	Clinical	2017-04-10	2017	III	clade3_group3	United Kingdom	Oxfordshire	UK	Unknown	Unknown	Unknown	clinical sample	SRR7976607	Eyre 2018		VF125AL	WT
Patient-032	Clinical	2017-04-26	2017	III	clade3_group3	United Kingdom	Oxfordshire	UK	Unknown	Unknown	Unknown	clinical sample	SRR7976580	Eyre 2018		VF125AL	WT
Patient-033-01	Clinical	2017-05-05	2017	III	clade3_group3	United Kingdom	Oxfordshire	UK	Unknown	Unknown	Unknown	clinical sample	SRR7976541	Eyre 2018		VF125AL	WT
Patient-034	Clinical	2017-05-22	2017	III	clade3_group3	United Kingdom	Oxfordshire	UK	Unknown	Unknown	Unknown	clinical sample	SRR7976556	Eyre 2018		VF125AL	WT
Patient-037	Clinical	2017-04-24	2017	III	clade3_group3	United Kingdom	Oxfordshire	UK	Unknown	Unknown	Unknown	clinical sample	SRR7976603	Eyre 2018		VF125AL	WT
Patient-038	Clinical	2017-01-25	2017	III	clade3_group3	United Kingdom	Oxfordshire	UK	Unknown	Unknown	Unknown	clinical sample	SRR7976545	Eyre 2018		VF125AL	WT
RI-A112	Clinical	2018-11-07	2018	III	clade3_group3	China	Shenyang	China	Resistant	Susceptible	Susceptible	Urine	SRR9316720	Tian 2021		VF125AL	WT
RICU1-A1	Clinical	2016-04-06	2016	III	clade3_group3	China	Shenyang	China	Resistant	Susceptible	Susceptible	Urine	SRR9316788	Tian 2021		VF125AL	WT
RICU10-A57	Clinical	2018-01-24	2018	III	clade3_group3	China	Shenyang	China	Resistant	Susceptible	Susceptible	Tracheal catheter	SRR9316775	Tian 2021		VF125AL	WT
RICU11-A62	Clinical	2018-03-02	2018	III	clade3_group3	China	Shenyang	China	Resistant	Susceptible	Susceptible	Sputum	SRR9316784	Tian 2021		VF125AL	WT
RICU12-A80	Clinical	2018-05-15	2018	III	clade3_group3	China	Shenyang	China	Resistant	Susceptible	Susceptible	Sputum	SRR9316798	Tian 2021		VF125AL	WT
RICU13-A83	Clinical	2018-05-31	2018	III	clade3_group3	China	Shenyang	China	Resistant	Susceptible	Susceptible	Sputum	SRR9316810	Tian 2021		VF125AL	WT
RICU14-A95	Clinical	2018-08-23	2018	III	clade3_group3	China	Shenyang	China	Resistant	Susceptible	Susceptible	Urinary catheter	SRR9316816	Tian 2021		VF125AL	WT
RICU15-A105	Clinical	2018-09-30	2018	III	clade3_group3	China	Shenyang	China	Resistant	Susceptible	Susceptible	Sputum	SRR9316795	Tian 2021		VF125AL	WT
RICU16-A106	Clinical	2018-10-05	2018	III	clade3_group3	China	Shenyang	China	Resistant	Susceptible	Susceptible	Sputum	SRR9316796	Tian 2021		VF125AL	WT
RICU17-A108	Clinical	2016-12-14	2018	III	clade3_group3	China	Shenyang	China	Resistant	Susceptible	Susceptible	Blood	SRR9316724	Tian 2021		VF125AL	WT
RICU18-A113	Clinical	2018-11-13	2018	III	clade3_group3	China	Shenyang	China	Resistant	Susceptible	Susceptible	Urinary catheter	SRR9316721	Tian 2021		VF125AL	WT
RICU19-A115	Clinical	2018-11-20	2018	III	clade3_group3	China	Shenyang	China	Resistant	Susceptible	Susceptible	Sputum	SRR9316723	Tian 2021		VF125AL	WT
RICU2-A2	Clinical	2016-06-03	2016	III	clade3_group3	China	Shenyang	China	Resistant	Susceptible	Susceptible	Urine	SRR9316744	Tian 2021		VF125AL	WT
RICU3-A3	Clinical	2016-06-17	2016	III	clade3_group3	China	Shenyang	China	Resistant	Susceptible	Susceptible	Sputum	SRR9316754	Tian 2021		VF125AL	WT
RICU4-A7	Clinical	2016-08-05	2016	III	clade3_group3	China	Shenyang	China	Resistant	Susceptible	Susceptible	Sputum	SRR9316779	Tian 2021		VF125AL	WT
RICU5-A12	Clinical	2016-12-07	2016	III	clade3_group3	China	Shenyang	China	Resistant	Susceptible	Susceptible	Urinary catheter	SRR9316729	Tian 2021		VF125AL	WT
RICU6-A14	Clinical	2017-01-26	2017	III	clade3_group3	China	Shenyang	China	Resistant	Susceptible	Susceptible	Urinary catheter	SRR9316738	Tian 2021		VF125AL	WT
RICU7-A22	Clinical	2017-07-29	2017	III	clade3_group3	China	Shenyang	China	Resistant	Susceptible	Susceptible	Urine	SRR9316746	Tian 2021		VF125AL	WT
RICU8-A38	Clinical	2017-10-10	2017	III	clade3_group3	China	Shenyang	China	Resistant	Susceptible	Susceptible	Urine	SRR9316765	Tian 2021		VF125AL	WT
RICU9-A52	Clinical	2017-12-31	2017	III	clade3_group3	China	Shenyang	China	Resistant	Susceptible	Susceptible	Blood	SRR9316772	Tian 2021		VF125AL	WT
S1	Clinical	2017-09-03	2017	III	clade3_group3	Spain	Valencia	Spain	Resistant	Unknown	Susceptible	Urinary catheter	ERR12321083	This study		VF125AL	WT
S103	Clinical	2020-02-04	2020	III	clade3_group3	Spain	Valencia	Spain	Resistant	Unknown	Susceptible	Urinary catheter	ERR12321104	This study		VF125AL	WT
S117	Clinical	2020-03-24	2020	III	clade3_group3	Spain	Valencia	Spain	Resistant	Unknown	Susceptible	Blood (venipuncture)	ERR12321086	This study		VF125AL	WT
S127	Clinical	2020-04-14	2020	III	clade3_group3	Spain	Valencia	Spain	Resistant	Unknown	Susceptible	Axillary-rectal exudate	ERR12321098	This study		VF125AL	WT
S129	Clinical	2020-05-04	2020	III	clade3_group3	Spain	Valencia	Spain	Resistant	Unknown	Susceptible	Blood (catheter)	ERR12321077	This study		VF125AL	WT
S131	Clinical	2020-06-09	2020	III	clade3_group3	Spain	Valencia	Spain	Resistant	Unknown	Susceptible	Axillary-rectal exudate	ERR12321100	This study		VF125AL	WT
S132	Clinical	2020-07-07	2020	III	clade3_group3	Spain	Valencia	Spain	Resistant	Unknown	Susceptible	Blood (catheter)	ERR12321108	This study		VF125AL	WT
S134	Clinical	2020-08-17	2020	III	clade3_group3	Spain	Valencia	Spain	Resistant	Unknown	Susceptible	Axillary-rectal exudate	ERR12321088	This study		VF125AL	WT
S135	Clinical	2020-09-09	2020	III	clade3_group3	Spain	Valencia	Spain	Resistant	Unknown	Susceptible	Blood (catheter)	ERR12321091	This study		VF125AL	WT
S138	Clinical	2020-11-30	2020	III	clade3_group3	Spain	Valencia	Spain	Resistant	Unknown	Susceptible	Blood (catheter)	ERR12321105	This study		VF125AL	WT
S14	Clinical	2018-02-06	2018	III	clade3_group3	Spain	Valencia	Spain	Resistant	Unknown	Susceptible	Blood (venipuncture)	ERR12321082	This study		VF125AL	WT
S140	Clinical	2020-12-18	2020	III	clade3_group3	Spain	Valencia	Spain	Resistant	Unknown	Susceptible	Blood (catheter)	ERR12321101	This study		VF125AL	WT
S141	Clinical	2021-01-12	2021	III	clade3_group3	Spain	Valencia	Spain	Resistant	Unknown	Susceptible	Axillary-rectal exudate	ERR12321094	This study		VF125AL	WT
S145	Clinical	2021-02-11	2021	III	clade3_group3	Spain	Valencia	Spain	Resistant	Unknown	Susceptible	Blood (catheter)	ERR12321107	This study		VF125AL	WT
S147	Clinical	2021-04-17	2021	III	clade3_group3	Spain	Valencia	Spain	Resistant	Unknown	Susceptible	Blood (catheter)	ERR12321089	This study		VF125AL	WT
S150	Clinical	2021-05-12	2021	III	clade3_group3	Spain	Valencia	Spain	Resistant	Unknown	Susceptible	Blood (venipuncture)	ERR12321093	This study		VF125AL	WT
S151	Environmental	2021-06-14	2021	III	clade3_group3	Spain	Valencia	Spain	Resistant	Unknown	Susceptible	Environmental	ERR12321079	This study		VF125AL	WT
S2	Clinical	2017-10-14	2017	III	clade3_group3	Spain	Valencia	Spain	Resistant	Unknown	Susceptible	Blood (catheter)	ERR12321099	This study		VF125AL	WT
S28	Clinical	2018-04-30	2018	III	clade3_group3	Spain	Valencia	Spain	Resistant	Unknown	Susceptible	Blood (catheter)	ERR12321090	This study		VF125AL	WT
S33	Clinical	2018-05-27	2018	III	clade3_group3	Spain	Valencia	Spain	Resistant	Unknown	Susceptible	Blood (catheter)	ERR12321092	This study		VF125AL	WT
S35	Clinical	2018-06-28	2018	III	clade3_group3	Spain	Valencia	Spain	Resistant	Unknown	Susceptible	Blood (venipuncture)	ERR12321075	This study		VF125AL	WT
S4	Clinical	2017-10-18	2017	III	clade3_group3	Spain	Valencia	Spain	Resistant	Unknown	Susceptible	Blood (venipuncture)	ERR12321102	This study		VF125AL	WT
S44	Clinical	2018-08-31	2018	III	clade3_group3	Spain	Valencia	Spain	Resistant	Unknown	Susceptible	Catheter	ERR12321096	This study		VF125AL	WT
S56	Clinical	2018-11-13	2018	III	clade3_group3	Spain	Valencia	Spain	Resistant	Unknown	Susceptible	Lumbar puncture	ERR12321097	This study		VF125AL	WT
S62	Clinical	2019-01-23	2019	III	clade3_group3	Spain	Valencia	Spain	Resistant	Unknown	Susceptible	Catheter	ERR12321081	This study		VF125AL	WT
S67	Clinical	2019-02-22	2019	III	clade3_group3	Spain	Valencia	Spain	Resistant	Unknown	Resistant	Blood (catheter)	ERR12321078	This study		VF125AL	S639Y
S72	Clinical	2019-04-02	2019	III	clade3_group3	Spain	Valencia	Spain	Resistant	Unknown	Susceptible	Urinary catheter	ERR12321076	This study		VF125AL	WT
S75	Clinical	2019-05-21	2019	III	clade3_group3	Spain	Valencia	Spain	Resistant	Unknown	Susceptible	Blood (catheter)	ERR12321103	This study		VF125AL	WT
S77	Clinical	2019-06-02	2019	III	clade3_group3	Spain	Valencia	Spain	Resistant	Unknown	Susceptible	Urine	ERR12321087	This study		VF125AL	WT
S8	Clinical	2017-12-12	2017	III	clade3_group3	Spain	Valencia	Spain	Resistant	Unknown	Susceptible	Blood (catheter)	ERR12321106	This study		VF125AL	WT
S81	Clinical	2019-08-16	2019	III	clade3_group3	Spain	Valencia	Spain	Resistant	Unknown	Susceptible	Blood (catheter)	ERR12321085	This study		VF125AL	WT
S82	Clinical	2019-09-05	2019	III	clade3_group3	Spain	Valencia	Spain	Resistant	Unknown	Susceptible	Catheter tip	ERR12321095	This study		VF125AL	WT
S86	Clinical	2019-10-18	2019	III	clade3_group3	Spain	Valencia	Spain	Resistant	Unknown	Susceptible	Blood (catheter)	ERR12321084	This study		VF125AL	WT
S94	Clinical	2019-12-05	2019	III	clade3_group3	Spain	Valencia	Spain	Resistant	Unknown	Susceptible	Blood (catheter)	ERR12321080	This study		VF125AL	WT
S99	Clinical	2020-01-29	2020	III	clade3_group3	Spain	Valencia	Spain	Resistant	Unknown	Susceptible	Blood (venipuncture)	ERR12321074	This study		VF125AL	WT
SICU1-A66	Clinical	2018-04-06	2018	III	clade3_group3	China	Shenyang	China	Resistant	Susceptible	Susceptible	Urine	SRR9316780	Tian 2021		VF125AL	WT
SICU2-A89	Clinical	2018-07-25	2018	III	clade3_group3	China	Shenyang	China	Resistant	Susceptible	Susceptible	Sputum	SRR9316815	Tian 2021		VF125AL	WT
TempProbe1	Environmental	2017-04-04	2017	III	clade3_group3	United Kingdom	Oxfordshire	UK	Unknown	Unknown	Unknown	Environmental	SRR7976593	Eyre 2018		VF125AL	WT
TempProbe2	Environmental	2017-05-16	2017	III	clade3_group3	United Kingdom	Oxfordshire	UK	Unknown	Unknown	Unknown	Environmental	SRR7976601	Eyre 2018		VF125AL	WT
TempProbe3	Environmental	2017-05-16	2017	III	clade3_group3	United Kingdom	Oxfordshire	UK	Unknown	Unknown	Unknown	Environmental	SRR7976602	Eyre 2018		VF125AL	WT
TempProbe4	Environmental	2017-05-16	2017	III	clade3_group3	United Kingdom	Oxfordshire	UK	Unknown	Unknown	Unknown	Environmental	SRR7976571	Eyre 2018		VF125AL	WT

TempProbe5	Environmental	2017-05-16	2017	III	clade3_group3	United Kingdon	Oxfordshire	UK	Unknown	Unknown	Unknown	Environmental	SRR7976560	Eyre 2018		VF125AL	WT
UCLA-A1	Clinical	2019-10-08	2019	III	clade3_group3	United States	California	US	Resistant	Resistant	Susceptible	inguinal/axillary swab	SRR12916694	Price 2021		VF125AL	WT
UCLA-A2	Clinical	2019-10-08	2019	III	clade3_group3	United States	California	US	Resistant	Resistant	Susceptible	Trachea	SRR12916693	Price 2021		VF125AL	WT
UCLA-C1	Clinical	2020-08-12	2020	III	clade3_group3	United States	California	US	Resistant	Resistant	Susceptible	inguinal/axillary swab	SRR12916692	Price 2021		VF125AL	WT
UCLA-D1	Clinical	2020-08-19	2020	III	clade3_group3	United States	California	US	Resistant	Resistant	Susceptible	inguinal/axillary swab	SRR12916691	Price 2021		VF125AL	WT
WM-18-177	Clinical	2018-07-01	2018	III	clade3_group3	South Africa	Unknown	NA	Resistant	Susceptible	Susceptible	Blood culture	SRR11485317	Biswas 2020		VF125AL	WT
WM-18-180	Clinical	2018-09-20	2018	III	clade3_group3	Australia	Sydney	NA	Resistant	Susceptible	Susceptible	nose swab	SRR11485320	Biswas 2020		VF125AL	WT
WM-18-181	Clinical	2018-09-05	2018	III	clade3_group3	Australia	Sydney	NA	Resistant	Susceptible	Susceptible	Wound swab PICC site	SRR11485334	Biswas 2020		VF125AL	WT
WM-18-182	Clinical	2018-09-05	2018	III	clade3_group3	Australia	Sydney	NA	Resistant	Susceptible	Susceptible	sputum	SRR11485333	Biswas 2020		VF125AL	WT
WM-18-187	Clinical	2018-09-06	2018	III	clade3_group3	Australia	Sydney	NA	Resistant	Susceptible	Susceptible	Axilla swab	SRR11485324	Biswas 2020		VF125AL	WT
WM-18-188	Clinical	2018-09-07	2018	III	clade3_group3	Australia	Sydney	NA	Resistant	Susceptible	Susceptible	sputum	SRR11485323	Biswas 2020		VF125AL	WT
WM-18-189	Clinical	2018-09-11	2018	III	clade3_group3	Australia	Sydney	NA	Resistant	Susceptible	Susceptible	Axilla swab	SRR11485322	Biswas 2020		VF125AL	WT
WM-18-190	Clinical	2018-09-11	2018	III	clade3_group3	Australia	Sydney	NA	Resistant	Susceptible	Susceptible	Groin	SRR11485321	Biswas 2020		VF125AL	WT
WM-18-197	Clinical	2018-11-08	2018	III	clade3_group3	Australia	Sydney	NA	Resistant	Susceptible	Susceptible	throat	SRR11485319	Biswas 2020		VF125AL	WT
